# Molecular Investigation of Canine Parvovirus-2 (CPV-2) Outbreak in Nevis Island: Analysis of the Nearly Complete Genomes of CPV-2 Strains from the Caribbean Region

**DOI:** 10.3390/v13061083

**Published:** 2021-06-06

**Authors:** Kerry Gainor, April Bowen, Pompei Bolfa, Andrea Peda, Yashpal S. Malik, Souvik Ghosh

**Affiliations:** 1 Department of Biomedical Sciences, Ross University School of Veterinary Medicine, Basseterre P.O. Box 334, Saint Kitts and Nevis; KerryGainor@students.rossu.edu (K.G.); pbolfa@rossvet.edu.kn (P.B.); 2 Nevis Animal Speak, Cades Bay Nevis, Basserrete, Saint Kitts and Nevis; aprilbowenlvt@outlook.com; 3 Department of Clinical Sciences, Ross University School of Veterinary Medicine, Basseterre P.O. Box 334, Saint Kitts and Nevis; apeda@rossvet.edu.kn; 4 College of Animal Biotechnology, Guru Angad Dev Veterinary and Animal Science University, Ludhiana, Punjab 141001, India; malikyps@gmail.com

**Keywords:** canine parvovirus, Caribbean region, new CPV-2a, outbreak, endemic, nearly complete genomes, virus evolution

## Abstract

To date, there is a dearth of information on canine parvovirus-2 (CPV-2) from the Caribbean region. During August–October 2020, the veterinary clinic on the Caribbean island of Nevis reported 64 household dogs with CPV-2-like clinical signs (hemorrhagic/non-hemorrhagic diarrhea and vomiting), of which 27 animals died. Rectal swabs/fecal samples were obtained from 43 dogs. A total of 39 of the 43 dogs tested positive for CPV-2 antigen and/or DNA, while 4 samples, negative for CPV-2 antigen, were not available for PCR. Among the 21 untested dogs, 15 had CPV-2 positive littermates. Analysis of the complete VP2 sequences of 32 strains identified new CPV-2a (CPV-2a with Ser297Ala in VP2) as the predominant CPV-2 on Nevis Island. Two nonsynonymous mutations, one rare (Asp373Asn) and the other uncommon (Ala262Thr), were observed in a few VP2 sequences. It was intriguing that new CPV-2a was associated with an outbreak of gastroenteritis on Nevis while found at low frequencies in sporadic cases of diarrhea on the neighboring island of St. Kitts. The nearly complete CPV-2 genomes (4 CPV-2 strains from St. Kitts and Nevis (SKN)) were reported for the first time from the Caribbean region. Eleven substitutions were found among the SKN genomes, which included nine synonymous substitutions, five of which have been rarely reported, and the two nonsynonymous substitutions. Phylogenetically, the SKN CPV-2 sequences formed a distinct cluster, with CPV-2b/USA/1998 strains constituting the nearest cluster. Our findings suggested that new CPV-2a is endemic in the region, with the potential to cause severe outbreaks, warranting further studies across the Caribbean Islands. Analysis of the SKN CPV-2 genomes corroborated the hypothesis that recurrent parallel evolution and reversion might play important roles in the evolution of CPV-2.

## 1. Introduction

Canine parvovirus-2 (CPV-2), members of the genus *Protoparvovirus* within the family *Parvoviridae*, are highly contagious enteric pathogens of household dogs, often causing fatal hemorrhagic gastroenteritis in puppies [1,2,3,4]. Morphologically, CPV-2 are small, nonenveloped viruses containing a single-stranded, negative-sense DNA genome (~5200 bp in size) [2,5]. The CPV-2 genome possesses at least two major open reading frames (ORFs), designated as ORF1 and ORF2. The ORF1 encodes two nonstructural (NS1 and NS2) proteins, while ORF2 codes for two structural (VP1 and VP2) proteins, translated through alternative splicing of the same viral mRNAs [2,5].

The CPV-2 nonstructural proteins have been shown to be crucial for viral replication, DNA packaging, cytotoxicity, and pathogenesis [6,7,8]. On the other hand, the structural proteins form the viral capsid, of which VP2 is the major component [9,10]. The VP2 protein plays important roles in determining host range, tissue tropisms, and virus-host interactions, and is highly antigenic, forming the basis of the currently licensed CPV-2 vaccines [2,3,11,12]. To date, the majority of the studies on CPV-2 are based on the VP2 encoding gene [2,13,14], while limited information is available on the genetic variations in the nonstructural genes [15,16].

Based on amino acid (aa) differences in the VP2 protein, CPV-2 strains have been classified into four major antigenic variants: CPV-2, CPV-2a, CPV-2b, and CPV-2c [2,13,14]. The earliest CPV-2 strains that emerged in dogs during the late 1970s are referred to as the CPV-2 variant [2,16]. By the end of 1980, CPV-2 was rapidly replaced by a new antigenic variant, designated as the CPV-2a variant [17,18]. The other CPV-2 variants, CPV-2b and CPV-2c, were first reported in 1984 and 2000, respectively [19,20].

The antigenic differences between CPV-2a, CPV-2b, and CPV-2c have been attributed to a single aa mismatch (Asn in CPV-2a, Asp in CPV-2b, and Glu in CPV-2c) at residue 426 of the VP2 protein [2]. However, by phylogenetic analysis of complete/nearly full-length CPV-2 sequences, the CPV-2a, CPV-2b, and CPV-2c variants lacked clear monophyletic segregation, and were grouped together into a single ‘CPV-2a clade’, which was distinct from the cluster of the earliest CPV-2 strains, referred to as the CPV-2 clade [16]. Other nonsynonymous mutations have also been observed in the CPV-2 variants [2,13,14,16,21]. Notable among these aa changes is the presence of Ser297Ala in VP2 of several CPV-2a and CPV-2b strains, sometimes referred to as new CPV-2a and new CPV-2b, respectively [13]. Currently, the CPV-2a, CPV-2b, and CPV-2c variants and their mutants, such as new CPV-2a and new CPV-2b, are circulating worldwide, with different relative frequencies between countries and between sampling periods [2,13,14,16,21,22,23].

To date, there is only a single report on the detection and molecular characterization of CPV-2 from the Caribbean region [24]. During February 2015–August 2016, new CPV-2a was detected (25/104 dogs tested CPV-2 positive, 20 samples were sequenced) in sporadic cases of diarrhea in household dogs on St. Kitts Island [24]. In the present study, we report a molecular investigation of a new CPV-2a associated severe outbreak of canine gastroenteritis in the Caribbean island of Nevis that resulted in the death of 27 animals.

Since there are no reports on complete CPV-2 genomes from the Caribbean region, the nearly full-length genomes of 3 CPV-2 strains (representing each of the new CPV-2a mutants with a nonsynonymous substitution in the VP2 gene) detected during the outbreak on Nevis and that of a previously reported CPV-2 strain from St. Kitts were analyzed in the present study.

## 2. Materials and Methods

### 2.1. Ethics Statement

The present study was submitted to the Institutional Animal Care and Use Committee (IACUC) of the Ross University School of Veterinary Medicine (RUSVM), St. Kitts Island. Ethical review and approval were waived for this study by the RUSVM IACUC as the research study was based on leftover samples that were collected for diagnostic purposes at the veterinary clinic on Nevis Island (RUSVM IACUC sample/tissue notification letter number TSU 1.23.21).

### 2.2. Sampling

**Figure 1:**
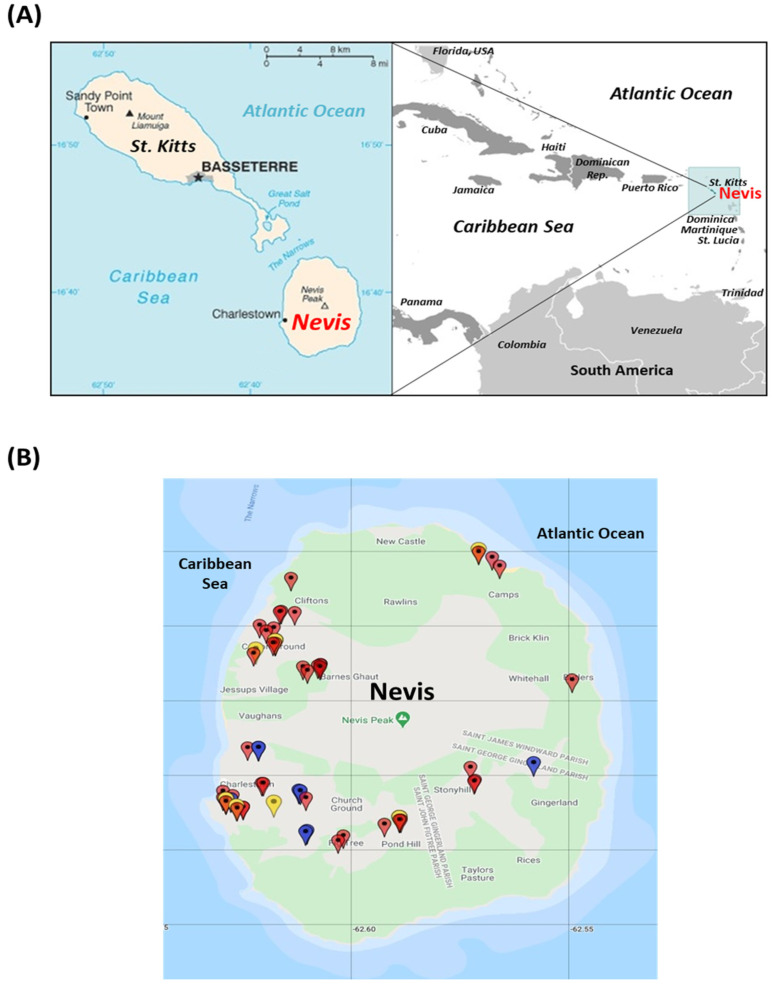
(**A**) Geographical location of Nevis Island. (**B**) Map of Nevis Island showing the locations of the 64 dogs (highlighted with pins) with canine parvovirus-2-like (CPV-2-like) clinical signs. Red pin, dogs that tested positive for CPV-2 by the SNAP^®^ Parvo Test (IDEXX, Westbrook, ME, USA) and/or PCR; Yellow pin, untested dogs with CPV-2-like clinical signs that had a CPV-2 positive littermate (by the SNAP^®^ Parvo Test and/or PCR); Blue, untested dogs with CPV-2-like clinical signs. The pins were inserted into the map using the Map Maker software (Maps.co, Mountain View, CA, USA). The maps for Figure 1A and Figure 1B were obtained from https://www.cia.gov/library/publications/the-world-factbook (accessed on 1 April 2021) and https://www.google.com/maps (accessed on 30 March 2021), respectively.

Nevis is a small Caribbean island (total area of 93 km², human population ~12,000) located in the lesser Antilles, and together with the neighboring island of St. Kitts, constitutes the twin Federation of St. Kitts and Nevis (https://www.paho.org/, accessed on 19 April 2021) (Figure 1A). Although there are no official records on the canine population in Nevis Island, many of the houses keep dogs as pets, with the island mix breed (a cross between a breed native to the Caribbean Islands and another canine breed) representing the majority of the domestic dogs.

From 1 August 2020 to 17 October 2020, 64 household dogs with CPV-2-like clinical signs were presented at the veterinary clinic on Nevis Island. A total of 44 rectal swabs/fecal samples were obtained from 43 of the dogs. One dog was sampled twice (5 September 2020 and 5 November 2020).

### 2.3. Screening

The samples were screened for the presence of CPV-2 antigen using the SNAP^®^ Parvo Test (IDEXX, Westbrook, ME, USA) following the manufacturer’s instructions.

### 2.4. Amplification of CPV-2 Genome

Viral DNA was extracted using the QIAamp Fast DNA Stool Mini Kit (Qiagen Sciences, Germantown, MD, USA) according to the manufacturer’s instructions. Primers used for amplification of the CPV-2 genome and/or obtaining CPV-2 genome sequences were designed in the present study (Supplementary Material S1). The complete VP2 ORF of CPV-2 was amplified by two overlapping PCRs, while three additional overlapping PCRs were employed to amplify the nearly full-length CPV-2 genomes (spanning all the coding regions, corresponding to nucleotide (nt) 272-nt 4585 of reference CPV-2 strain CPV-N (GenBank accession number M19296)) (Supplementary Material S1). PCRs were performed using the Platinum™ Taq DNA Polymerase (Invitrogen™, Thermo Fisher Scientific Corporation, Waltham, MA, USA) following the instructions provided by the manufacturer. Sterile water was used as a negative control during the PCR reactions.

### 2.5. Nucleotide Sequencing

The PCR amplicons were purified using the Wizard^®^ SV Gel and PCR Clean-Up kit (Promega, Madison, WI, USA) according to the manufacturer’s instructions and sequenced in both directions using forward and reverse primers (Supplementary Material S1). Nucleotide sequences were obtained using the ABI Prism Big Dye Terminator Cycle Sequencing Ready Reaction Kit (Applied Biosystems, Foster City, CA, USA) on an ABI 3730XL Genetic Analyzer (Applied Biosystems, Foster City, CA, USA).

### 2.6. Sequence Analysis

Putative ORFs encoding the CPV-2 NS1 and VP2 proteins were identified using the ORF finder (https://www.ncbi.nlm.nih.gov/orffinder/, accessed on 5 April 2021), while those coding for NS2 and VP1 proteins were determined by alignment of the obtained CPV-2 nt sequences with published CPV-2 coding sequences. The standard BLASTN and BLASTP program (Basic Local Alignment Search Tool, www.ncbi.nlm.nih.gov/blast, accessed on 2 April 2021) was used to perform a homology search for related cognate nt and deduced aa sequences, respectively. Multiple alignments of nt and deduced aa sequences were carried out using the CLUSTALW program (version ddbj, http://clustalw.ddbj.nig.ac.jp/, accessed on 2 April 2021) with default parameters. The nearly complete CPV-2 genome sequences were examined for recombination events using the RDP4 program with default parameters, as described previously [16,25]. Briefly, a CPV-2 sequence was identified as a recombinant if it was supported by two or more detection methods (3Seq, BOOTSCAN, CHIMERA, GENECONV, MAXCHI, RDP, and SISCAN) with a highest acceptable *p*-value of *p* < 0.01 with Bonferroni’s correction.

A data set (excluding recombinant sequences) of 210 nearly complete CPV-2 sequences from domestic and wild canids and 6 FPV sequences were created for phylogenetic analysis, based on those reported in previous studies [16,21]. Phylogenetic analysis was performed by the maximum likelihood (ML) method using the MEGA7 software [26], with gamma-distributed rate variation among sites and 1000 bootstrap replicates. Phylogenetic trees were constructed using both the Hasegawa-Kishino-Yano (HKY) and general time-reversible (GTR) substitution models.

### 2.7. GenBank Accession Numbers

The GenBank accession numbers for the CPV-2 genome sequences determined in this study are MW595661-MW595693 (complete VP2 ORF sequences) and MW616469-MW616472 (nearly full-length CPV-2 genome sequences).

## 3. Results and Discussion

### 3.1. Molecular Investigation of CPV-2 Outbreak in Nevis Island

From 1 August 2020 to 17 October 2020, the veterinary clinic on Nevis Island reported 64 household dogs with anorexia, gastroenteritis (with or without blood in feces), lethargy, and vomiting (CPV-2-like clinical signs). A total of 27 (42.2%) of the dogs died. Based on case histories, the outbreak of gastroenteritis appeared to have started at Charlestown, the capital of Nevis, and eventually spread to other parts of the island, with most cases mapped to the more densely populated western coastal region of the island (Figure 1B).

A total of 43 dogs were tested for CPV-2 antigen using the SNAP^®^ Parvo Test (IDEXX, Westbrook, ME, USA), of which 32 samples were available for PCR. A total of 39 (90.6%) of the 43 dogs tested CPV-2 positive by SNAP^®^ Parvo Test (24/43 dogs, 55.8%) and/or PCR (32/32 dogs, 100%) (Table 1), while 4 samples that were negative for CPV-2 antigen could not be tested by PCR. Fifteen of the PCR positive dogs tested negative by the SNAP^®^ Parvo Test (Table 1), which might be attributed to the lower sensitivities of the CPV-2 antigen tests compared to PCR/qPCR assays, even in dogs with CPV-2-like clinical signs, as reported in previous studies [2,27]. We did not observe any differences in clinical severity between dogs that tested negative and positive to the SNAP^®^ Parvo Test. Although samples could not be obtained from 21 dogs with CPV-2-like clinical signs, 15 of the animals had littermates that were positive for CPV-2 antigen and/or DNA. Most of the sick dogs were <6 months of age (89%, 57/64 dogs with CPV-2-like clinical signs) and were either not vaccinated or received incomplete immunization (did not receive all doses of the vaccine) against CPV-2 (95.3%, 61/64 dogs with CPV-2-like clinical signs), corroborating previous observations on the increased risk of CPV-2 infection in unvaccinated puppies [2,4]. Since not all the dogs were tested for CPV-2, and none of the obtained samples were screened for other enteric pathogens, we could not establish whether CPV-2 was the sole etiological agent in the outbreak of gastroenteritis on Nevis Island.

In order to determine the CPV-2 variant/s circulating during the outbreak in Nevis, complete VP2 ORF sequences were obtained from the 32 PCR positive samples. Based on the presence of 297Ala and 426Asn in the putative VP2 proteins [13], all the CPV-2 strains from Nevis were classified as new CPV-2a (Table 2; Supplementary Material S2). In a previous study (2015–2016), new CPV-2a was only identified in sporadic cases of diarrhea on the neighboring island of St. Kitts [24]. These observations suggested that new CPV-2a is endemic and might be predominant in this part of the world. In South America, new CPV-2a, or other mutants of CPV-2a (VP2 Ser297Asn, Phe267Tyr, Tyr324Ile, and Thr440Ala) have been found to coexist with CPV-2b and/or CPV-2c variants at various frequencies, emerging as the major strain in a few studies [13,21,28,29,30,31,32,33]. In studies from North (Canada and USA) and Central (Mexico) Americas, CPV-2b, or CPV-2c was most prevalent, while a single report from Alaska detected both new CPV-2a and new CPV-2b in an outbreak of canine gastroenteritis [13,34,35,36,37]. Taken together, the molecular epidemiology of CPV-2 in the Caribbean region appears to differ from those reported in nearby North, Central, and South American countries, although further studies on the other islands are required to validate this observation.

The new CPV-2a strains from Nevis shared absolute deduced VP2 aa identities between themselves and those of new CPV-2a strains reported previously from St. Kitts [24], except for Ala262Thr in 4 CPV-2 strains and Asp373Asn in 3 other CPV-2 strains (Table 2; Supplementary Material S2). Although the significance of the aa at residue 262 of VP2 is not yet known, VP2 Ala262Thr has been described as a novel mutation [38], reported in new CPV-2a and new CPV-2b strains from Western Australia [38], two new CPV-2a strains from India (GenBank accession numbers DQ182624 and KU866399), and a single new CPV-2a strain from China (MH177301). The other nonsynonymous mutation, VP2 Asp373Asn, has been rarely reported in CPV-2 sequences, found in two new CPV-2a strains (one each from Australia (MN259033) and Thailand (GQ379047)), a single CPV-2 strain from a cat in Taiwan (KY010491), and two feline panleukopenia virus strains (FPV) (MH559110 and MK570710). The aa residue at 373 of VP2 is located within the VP2 flexible loop (a surface loop between VP2 residues 359 and 375), which is a pH-sensitive structure that governs binding to divalent ions in FPV and CPV-2 [39,40]. Structural studies on FPV at pH 7.5 have shown that the ion density, adjacent to the flexible loop, is coordinated by Asp 373 and Asp 375, and carbonyl oxygen atoms of Arg 361 and Gly 362 [39]. However, the implication/s of VP2 Asp373Asn remains to be determined. In addition to the two aa mismatches, five synonymous mutations were observed among the VP2 sequences of the new CPV-2a strains from Nevis (Supplementary Material S3).

Following natural CPV-2 infection or immunization with the commercially available modified live virus (MLV) vaccines, dogs have been shown to shed viral DNA for as long as 50 days post-infection [41]. In the present study, one of the dogs was sampled twice (5 September 2020 and 5 November 2020), during hemorrhagic gastroenteritis and after two months, when it was apparently healthy and presented at the clinic for vaccination (Table 1). Both the samples (CN27 and CN40) tested positive for CPV-2 by PCR. Surprisingly, the new CPV-2a strain with VP2 Ala262Thr was identified in the first sample, while the new CPV-2a with VP2 262Ala, detected in the majority of the samples from Nevis and during 2015-2016 in St. Kitts, was detected in the second sample (Table 2). Vaccinated dogs with protective antibody titers have been shown to shed low amounts of CPV-2 field strains in the feces [41,42]. Although the viral DNA was not quantified by qPCR, we observed weak amplification following PCR of the second sample, indicating a low viral load. Therefore, it might be possible that the dog developed immunity following initial infection with the new CPV-2a VP2 Ala262Thr, and eventually was asymptomatically infected with the new CPV-2a VP2 262Ala strain. Alternatively, reversion of VP2 Ala262Thr to the more prevalent new CPV-2a VP2 262Ala might be possible, as reverse mutations have been described in the CPV-2 genome in previous studies [13,16,38,43].

Most of the animals in the present study were not vaccinated or received incomplete vaccination. On the other hand, 3 CPV-2 positive dogs that were completely immunized (vaccine NOBIVAC^®^ CANINE 1-DAPPv, Merck Animal Health, Elkhorn, NE, USA) against the virus suffered from severe clinical disease (Table 1). The vaccine NOBIVAC^®^ CANINE 1-DAPPv contains a CPV-2b variant, while the dogs were infected with new CPV-2a. Although the efficacy of the commercially available CPV-2 MLV vaccines against the different CPV-2 antigenic variants has been debated, they have been shown to be effective in significantly reducing the clinical severity of CPV-2 disease caused by the non-vaccine field variants [3]. Since the dogs were aged ≥ 6 months, it is unlikely that maternal antibodies interfered with the efficacy of the vaccine. Other factors, such as vaccine-related errors (issues with vaccine storage, transport and/or administration) and/or host-related factors (impaired immune status, non-responders, and/or malnutrition), might have contributed to vaccine failure [3].

In a previous study on St. Kitts, new CPV-2a was sporadically detected in 20 diarrheic dogs (25/104 dogs were CPV-2 positive; 5 samples could not be sequenced) during a period of 1 year and 7 months [24]. During and around the duration of the outbreak in Nevis, the veterinary clinic on St. Kitts reported only five sporadic cases of CPV-2 infection (three and two dogs tested CPV-2 positive by the SNAP^®^ Parvo Test and PCR, respectively), of which two dogs died (Supplementary Material S4). Analysis of the complete CPV-2 VP2 sequences from the two dead dogs (strain CK81, GenBank accession number MW616470, and strain CK84, MW616472) revealed 100% deduced aa identities with those of the new CPV-2a reported in our study (Supplementary Material S2). Since the canine breeds, environmental conditions, vaccination trends, husbandry practices, and veterinary care are similar between the two islands, we found it intriguing that new CPV-2a was associated with an outbreak of gastroenteritis on Nevis, while found at low frequencies in sporadic cases of diarrhea on the neighboring island of St. Kitts.

There is limited movement of animals between St. Kitts and Nevis, as the twin islands are connected by ferry service. It might be possible that a new CPV-2a was recently introduced into Nevis from St. Kitts and that the canine population on Nevis Island was naive to infection with the virus, resulting in an outbreak situation. However, sporadic cases of CPV-2 have been previously reported in Nevis (based on old case records at the veterinary clinic in Nevis), although none of the samples were molecularly characterized to identify the CPV-2 variant. Therefore, we could not determine whether a new CPV-2a was circulating in Nevis before the outbreak. Furthermore, based on the available information, we could not ascertain if the two nonsynonymous mutations (Ala262Thr and Asp373Asn) in the VP2 genes of a few new CPV-2a strains appeared during the outbreak or were already circulating at low frequencies in the island canine population. Nevis has a sizable population of stray dogs, which could have facilitated the spread of the virus across the island.

### 3.2. Analysis of the Nearly Complete Genomes of CPV-2 Strains from St. Kitts and Nevis Islands

Considering the lack of information on CPV-2 genomes from the Caribbean region, we decided to determine the nearly full-length CPV-2 genome sequences (4269 nt, possessing the entire NS and VP coding regions) of 2 strains representing the new CPV-2a circulating in St. Kitts and Nevis (strain CN10 from the outbreak in Nevis, and strain RVC50 from our previous study in St. Kitts [24]), and one of each of the new CPV-2a with a nonsynonymous mutation in the VP2 gene (strain CN20 with VP2 Ala262Thr and strain CN14 with VP2 Asp373Asn).

The nearly complete genomes of CPV-2 strains from St. Kitts and Nevis (henceforth, collectively referred to as SKN strains) shared nt sequence identities of 99.77–99.93% between themselves (Supplementary Material S5). Absolute deduced NS1 and NS2 aa identities were observed between the SKN strains, while the putative VP1/VP2 proteins of strains CN14 and CN20 differed in an aa residue with those of the other SKN strains (Table 2 and Table 3; Supplementary Material S6). By BLASTN analysis, the SKN strains shared ≥ 99% nt sequence identities with several CPV-2a, CPV-2b, and CPV-2c variants, corroborating previous observations that the complete/nearly complete genomes of all CPV-2 variants are ~99% identical in nt sequence [16]. Although the SKN strains were assigned to new CPV-2a, they shared higher nt sequence identities with the nearly complete genomes of CPV-2c (99.46–99.63% with GenBank accession number KX434458) and CPV-2b (99.39–99.58% with EU659121) strains than those of CPV-2a variants (identities of ≤99.32–99.48% with EU659118).

A total of 11 substitutions (5 and 6 within the NS and VP coding region, respectively) were found among the nearly complete SKN CPV-2 genome sequences that included the two nonsynonymous substitutions in the VP coding region (Table 3, Supplementary Materials S5 and S6). The evolution of CPV-2 has been characterized by only a limited number of substitutions that became fixed or widespread during the last 40 years since the emergence of the virus [16]. A previous study identified 38 mutations (15 synonymous and 23 nonsynonymous mutations) that differed in an nt from the earliest CPV-2 strains (the CPV-2 antigenic variant) and were present in >10% of other CPV-2 strains (CPV-2a, CPV-2b, and CPV-2c variants) [16]. In the present study, 35 substitutions (24 synonymous and 11 nonsynonymous substitutions) were observed between the SKN CPV-2 sequences and that of strain CPV12 (representing the earliest CPV-2 strains from the late 1970s, GenBank accession number MN451655), of which 5 synonymous and 1 nonsynonymous substitution have been rarely reported in other CPV-2 strains (<10 CPV-2 sequences) (Table 3, Supplementary Materials S5 and S6). All the four SKN strains retained the five nonsynonymous substitutions in the VP coding region that was characteristic of the global sweep from CPV-2 to CPV-2a during the late 1980s (Table 3, Supplementary Material S5) [16,18].

**Figure 2:**
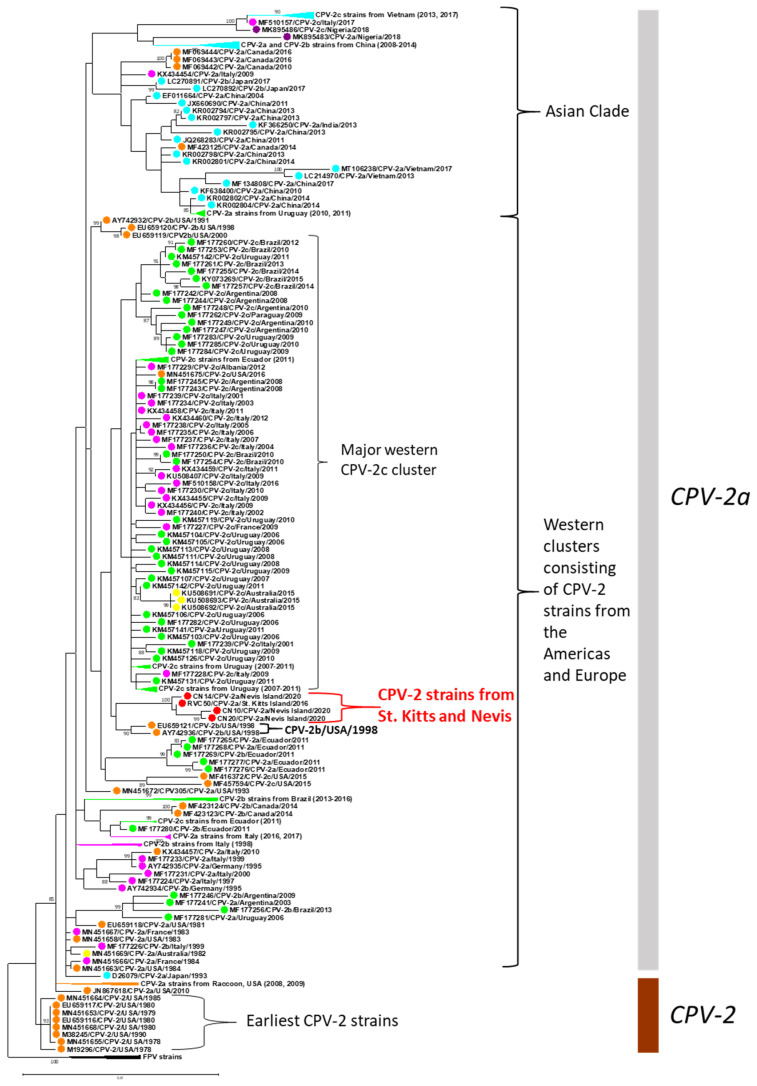
Phylogenetic analysis of the nearly complete genomes of canine parvovirus-2 (CPV-2) strains from St. Kitts and Nevis with those of other CPV-2 strains. The tree was created using the Hasegawa-Kishino-Yano model with gamma-distributed rate variation among sites and 1000 bootstrap replicates. The name of the strain/CPV-2 antigenic variant/place/year of detection is shown for the CPV-2 strains from St. Kitts and Nevis, while the GenBank accession number/CPV-2 antigenic variant/place/year of detection have been mentioned for the other CPV-2 strains. The two major phylogenetic clades (CPV-2 and CPV-2a) are demarcated with a brown and a light gray bar, respectively. The CPV-2 clade consists of the earliest CPV-2 strains from the late 1970s (the CPV-2 antigenic variants), while the ‘CPV-2a clade’ is composed of the CPV-2a, CPV-2b, and CPV-2c antigenic variants [16]. Feline panleukopenia virus (FPV) strains were included in the analysis. Sky blue, purple, yellow, pink, orange, green, and red circles indicate that the CPV-2 strain was detected in Asia, Africa, Australia, Europe, North America, South America, and St. Kitts and Nevis, respectively. Scale bar, 0.01 substitutions per nucleotide. Bootstrap values of <70 are not shown. Some of the clusters have been compressed to accommodate the entire tree. The complete phylogenetic tree is shown in Supplementary Material S7. Phylogenetic analysis using the General Time Reversible model is shown in Supplementary Material S8.

Since recombinant sequences might influence the outcomes of phylogenetic analysis [16,21], the nearly complete SKN CPV-2 genome sequences were screened for recombination events, as described previously [16,25]. However, no recombination breakpoints were detected in any of the SKN CPV-2 sequences. To rule out biases in clustering patterns, phylogenetic trees were created using both the HKY and GTR substitution models, as the HKY model was determined as the best-fit model using the ‘find best model’ function of MEGA7, while the GTR model was employed in a recent, important study on the evolution of CPV-2 [16]. Similar clustering patterns were observed with both the models (Figure 2; Supplementary Materials S7 and S8). Findings from previous studies, such as (i) phylogenetic classification of CPV-2 strains into two major clades: CPV-2 (comprising the earliest CPV-2 strains from the late 1970s) and CPV-2a (consisting of CPV-2a, CPV-2b, and CPV-2c variants) [16], (ii) a lack of phylogenetic resolution within the ‘CPV-2a clade’, characterized by low bootstrap values for most clusters [15,16], (iii) a lack of monophyletic segregation based on CPV-2 variants (CPV-2a, CPV-2b, and CPV-2c) [15,16], and (iv) presence of Asian and Western (CPV-2 sequences from the Americas and Europe) clades [21,44] were retained in the phylogenetic analysis (Figure 2; Supplementary Materials S7 and S8). Phylogenetically, the new CPV-2a SKN strains were placed with the Western CPV-2 strains and retained the signature aa residues that have been previously described as evolutionarily significant and characteristic to the Western clades (Figure 2; Table 4; Supplementary Materials S7 and S8) [21,44]. Within the Western clade, the SKN strains formed a distinct cluster. The nearest cluster to the SKN strains was that of CPV-2b strains detected in the USA during 1998, followed by the major Western CPV-2c cluster (consisting of primarily South American and Italian strains, designated as Western clade-III in a previous study [44]). However, the clade consisting of the SKN strains and the CPV-2b/USA/1998 strains was supported by a low bootstrap value (bootstrap value of 45 and 47 by the GTR and HKY models, respectively). The clustering patterns were corroborated by nt sequence identities and the geographical proximity of St. Kitts and Nevis to the USA and the South American countries. Except for VP2 Ala262Thr (strain CN20) and VP2 Asp373Asn (strain CN14), only a single nonsynonymous mutation (VP2 residue 426) that constitutes the basis of differentiation of CPV-2 into the CPV-2a, CPV-2b, and CPV-2c antigenic variants was observed between the new CPV-2a SKN strains and CPV-2 strains belonging to the CPV-2b/USA/1998 cluster, or the major Western CPV-2c cluster.

Corroborating previous observations [15,16,21,44], phylogenetically, the clustering of the nearly complete SKN CPV-2 genomes with those of other CPV-2 strains did not correspond to the clustering patterns based on VP2 coding sequences (Figure 2; Supplementary Materials S7–S9). The complete VP2 nt sequences of the SKN strains clustered near that of CPV-2a strain CPV-435/USA/2003 (GenBank accession number AY742953), although the clade was supported by a low bootstrap value of 45 (Supplementary Material S9). Since the complete/nearly complete genome sequence of CPV-435/USA/2003 was not available in the GenBank database, we could not include the strain in the analysis of nearly complete CPV-2 genomes.

Taken together, these findings supported previous observations that recurrent parallel evolution and reversion might play important roles in the evolution of CPV-2 and that the recently circulating CPV-2 strains are minor variants of a common ‘pan genome’ template that emerged after the global sweep from CPV-2 to CPV-2a [15,16].

It is likely that CPV-2 was imported into St. Kitts and Nevis Islands from another country. Since more humans (and pet dogs) travel to St. Kitts from the USA than from any other country, it might be possible the virus was introduced into the twin islands from the USA. On the other hand, St. Kitts and Nevis have a large population of the small Indian mongoose (*Urva auropunctata*) that thrive in wild, rural, and urban habitats [45]. A previous study had reported high rates of detection of parvovirus DNA (58%, n = 99) and antibodies (90%, n = 20) in the Egyptian mongoose (*Herpestes ichneumon*) [46]. Considering the role of wild canids as a potential source of CPV-2 to pet dogs [2,13,17,46,47], it would be interesting to investigate whether the new CPV-2a and/or new CPV-2a mutants (VP2 Ala262Thr and VP2 Asp373Asn) circulating in dogs on St. Kitts and Nevis were derived from the local mongoose population.

### 3.3. Conclusions

Although the currently licensed CPV-2 vaccines have been extensively used in veterinary practice and shown to confer protection against the different CPV-2 antigenic variants, CPV-2 continues to remain one of the leading causes of mortality and morbidity in domestic dogs [2,3,4]. To date, there is a dearth of data on CPV-2 from the Caribbean region. Our findings suggested that new CPV-2a might be endemic in this part of the world, with the potential to cause severe outbreaks facilitated by low vaccination rates among the canine population in the region. Recently, the neighboring islands of St. Eustatius and St. Lucia experienced large outbreaks of CPV-2 [48,49]. However, no information was available on the nature of CPV-2 variant/s circulating during the outbreaks. These observations underscore the importance of continuous molecular epidemiological studies on CPV-2 in all the Caribbean Islands alongside creating public awareness on the disease and vaccination.

The nearly complete CPV-2 genomes were reported for the first time from the Caribbean region, providing important insights into the overall genetic makeup of the CPV-2 strains circulating in St. Kitts and Nevis, especially the identification of six substitutions (five synonymous and one nonsynonymous substitution) that have been rarely reported in other CPV-2 sequences. Overall, analysis of the SKN CPV-2 genomes corroborated the hypothesis that recurrent parallel evolution and reversion might play important roles in the evolution of CPV [15,16]. Once again, it was revealed that the analysis of CPV-2 VP2 aa sequences do not reflect the true evolutionary patterns of CPV-2 strains.

## Figures and Tables

**Table 1:** Details of the dogs that tested positive for canine parvovirus-2 (CPV-2) by the SNAP^®^ Parvo Test (IDEXX, Westbrook, ME, USA) and/or PCR on Nevis Island. The CPV-2 strains from Nevis are denoted with the prefix CN (Canine Nevis).

Sample/Strain	Date of Sampling	Age	Sex	Breed	Vaccination against CPV-2 ^1^	SNAP^®^ Parvo Test ^2^	PCR ^3^	GenBank Accession Number (VP2 Gene, Complete ORF)	CPV-2 Variant
CN1	1 August 2020	3 months	Female	Shih Tzu	None	Positive	Not performed	Not sequenced	Not determined
CN2	11 August 2020	4 months	Male	Bulldog	Incomplete ^4^	Positive	Not performed	Not sequenced	Not determined
CN3	14 August 2020	2 months	Male	Pit bull	None	Positive	Not performed	Not sequenced	Not determined
CN4	15 August 2020	3 months 15 days	Male	Pit bull	None	Positive	Not performed	Not sequenced	Not determined
CN5	15 August 2020	4 months	Female	Pit bull	None	Positive	Not performed	Not sequenced	Not determined
CN6	21 August 2020	3 months	Female	Pit bull	Not available	Positive	Not performed	Not sequenced	Not determined
CN7	22 August 2020	2 months	Male	Island mix ^5^	None	Positive	Positive	MW595661	New CPV-2a ^6^
CN8	24 August 2020	2 months 15 days	Male	Pit bull	None	Negative	Positive	MW595662	New CPV-2a
CN9	25 August 2020	2 months	Female	Pit bull	None	Positive	Not performed	Not sequenced	Not determined
CN10	26 August 2020	4 months	Male	Island mix	None	Positive	Positive	MW595663	New CPV-2a
CN11	28 August 2020	4 months 15 days	Male	Island mix	Incomplete ^4^	Positive	Positive	MW595664	New CPV-2a
CN12	28 August 2020	7 months	Male	Pit bull	None	Positive	Positive	MW595665	New CPV-2a
CN13	28 August 2020	2 year	Male	Rottweiler mix	None	Negative	Positive	MW595666	New CPV-2a
CN14	28 August 2020	7 months	Female	Island mix	Not available	Positive	Positive	MW595667	New CPV-2a
CN15	29 August 2020	2 months 15 days	Female	Island mix	None	Negative	Positive	MW595668	New CPV-2a
CN16	1 September 2020	10 months	Male	Island mix	Complete	Positive	Positive	MW595669	New CPV-2a
CN17	1 September 2020	2 months	Male	Bulldog x Mastiff	None	Positive	Positive	MW595670	New CPV-2a
CN18	1 September 2020	4 months	Male	Island mix	None	Negative	Positive	MW595671	New CPV-2a
CN19	1 September 2020	4 months	Female	Pit bull	Not available	Negative	Positive	MW595672	New CPV-2a
CN20	1 September 2020	4 months	Male	Pit bull	Not available	Positive	Positive	MW595673	New CPV-2a
CN21	2 September 2020	1 year	Female	Island mix	None	Negative	Positive	MW595674	New CPV-2a
CN22	3 September 2020	6 months	Male	Island mix	Complete	Positive	Positive	MW595675	New CPV-2a
CN23	3 September 2020	2 months	Female	Pit bull	None	Negative	Positive	MW595676	New CPV-2a
CN24	3 September 2020	4 months	Female	Not available	None	Negative	Positive	MW595677	New CPV-2a
CN25	4 September 2020	2 months	Female	Island mix	None	Negative	Positive	MW595678	New CPV-2a
CN26	4 September 2020	2 months	Female	Island mix	None	Negative	Positive	MW595679	New CPV-2a
CN27 ^7^	5 September 2020	1 month 15 days	Male	Great Dane	None	Negative	Positive	MW595680	New CPV-2a
CN28	8 September 2020	6 months	Male	Pit bull	None	Positive	Positive	MW595681	New CPV-2a
CN29	11 September 2020	2 months	Male	Pit bull	None	Positive	Positive	MW595682	New CPV-2a
CN30	13 September 2020	2 months	Male	Pit bull	None	Positive	Positive	MW595683	New CPV-2a
CN31	14 September 2020	7 months	Female	Pit bull mix	Complete	Positive	Positive	MW595684	New CPV-2a
CN32	25 September 2020	2 months	Female	Island mix	None	Negative	Positive	MW595685	New CPV-2a
CN33	29 September 2020	2 months 15 days	Female	Island mix	None	Positive	Positive	MW595686	New CPV-2a
CN34	7 October 2020	7 months	Male	Island mix	None	Positive	Positive	MW595687	New CPV-2a
CN35	8 October 2020	4 months	Female	Island mix	Incomplete ^4^	Positive	Positive	MW595688	New CPV-2a
CN36	8 October 2020	4 months	Male	Island mix	None	Negative	Positive	MW595689	New CPV-2a
CN37	9 October 2020	4 months	Male	Island mix	None	Negative	Positive	MW595690	New CPV-2a
CN38	9 October 2020	2 months	Male	Island mix	Incomplete ^4^	Negative	Positive	MW595691	New CPV-2a
CN39	17 October 2020	1 month 15 days	Male	Island mix	None	Positive	Positive	MW595692	New CPV-2a
CN40 ^7^	5 November 2020	3 months 15 days	Male	Great Dane	None	Negative	Positive	MW595693	New CPV-2a

^1^ Indicates vaccination status at the time of sampling; ^2^ The SNAP^®^ Parvo Test (IDEXX, Westbrook, ME, USA) detects CPV-2 antigen in feces; ^3^ Based on amplification of the CPV-2 VP2 gene (Supplementary Material S1); ^4^ Did not receive all doses of the vaccine, as recommended by the American Animal Hospital Association (https://www.aaha.org/globalassets/02-guidelines/canine-vaccination, accessed on 2 March 2021); ^5^ Indicates a cross between a local canine breed and another breed; ^6^ CPV-2a with Ser297Ala in VP2; ^7^ Samples CN27 (collected during hemorrhagic gastroenteritis) and CN40 (collected from an asymptomatic animal) were from the same dog.

**Table 2:** Comparison of key amino acid (aa) residues of putative VP2 proteins of canine parvovirus-2 (CPV-2) strains detected on Nevis Island with those of other CPV-2 strains. Strain RVC50 represents the new CPV-2a (CPV-2a with Ser297Ala in VP2) strains detected during a previous study (February 2015–August 2016) in the neighboring island of St. Kitts [24]. The CPV-2 strains from Nevis are shown with italic font, while strain RVC50 from St. Kitts is underlined. Identical aa residues are shown with the same color. Amino acid mismatches between the CPV-2 strains from Nevis and strain RVC50 are shown in red font. Positions of aa residues correspond to those of strain CPV-b/USA/1978. Alignment of the deduced VP2 aa and ORF sequences of the CPV-2 strains from Nevis is shown in Supplementary Materials S2 and S3, respectively.

Amino Acid Position	87	101	262	267	297	300	305	321	324	373	375	426	440	555	570	Variant
Strain/Place/Year																
CPV-b/USA/1978	Met	Ile	Ala	Phe	Ser	Ala	Asp	Asn	Tyr	Asp	Asn	Asn	Thr	Val	Lys	CPV-2
CPV-15/USA/1984	Leu	Thr	Ala	Phe	Ser	Gly	Tyr	Asn	Tyr	Asp	Asp	Asn	Thr	Ile	Lys	CPV-2a
CPV-39/USA/1984	Leu	Thr	Ala	Phe	Ser	Gly	Tyr	Asn	Tyr	Asp	Asp	Asp	Thr	Val	Lys	CPV-2b
219/08-13/ITA/2008	Leu	Thr	Ala	Phe	Ala	Gly	Tyr	Asn	Tyr	Asp	Asp	Glu	Thr	Val	Lys	CPV-2c
CPV-435/USA/2003	Leu	Thr	Ala	Phe	Ala	Gly	Tyr	Asn	Tyr	Asp	Asp	Asn	Thr	Val	Lys	New CPV-2a
RVC50/St. Kitts/2016	Leu	Thr	Ala	Phe	Ala	Gly	Tyr	Asn	Tyr	Asp	Asp	Asn	Thr	Val	Lys	New CPV-2a
GX304/CHN/2017	Leu	Thr	Thr	Tyr	Ala	Gly	Tyr	Asn	Ile	Asp	Asp	Asn	Ala	Val	Lys	New CPV-2a
Beaumaris/AUS/2017	Leu	Thr	Ala	Phe	Ala	Gly	Tyr	Asn	Ile	Asn	Asp	Asn	Thr	Val	Lys	New CPV-2a
CPV-436/USA/2003	Leu	Thr	Ala	Phe	Ala	Gly	Tyr	Asn	Tyr	Asp	Asp	Asp	Thr	Val	Lys	New CPV-2b
*CN7/Nevis/2020*	Leu	Thr	Ala	Phe	Ala	Gly	Tyr	Asn	Tyr	Asp	Asp	Asn	Thr	Val	Lys	New CPV-2a
*CN8/Nevis/2020*	Leu	Thr	Ala	Phe	Ala	Gly	Tyr	Asn	Tyr	Asp	Asp	Asn	Thr	Val	Lys	New CPV-2a
*CN10/Nevis/2020*	Leu	Thr	Ala	Phe	Ala	Gly	Tyr	Asn	Tyr	Asp	Asp	Asn	Thr	Val	Lys	New CPV-2a
*CN11/Nevis/2020*	Leu	Thr	Ala	Phe	Ala	Gly	Tyr	Asn	Tyr	Asp	Asp	Asn	Thr	Val	Lys	New CPV-2a
*CN12/Nevis/2020*	Leu	Thr	Ala	Phe	Ala	Gly	Tyr	Asn	Tyr	Asp	Asp	Asn	Thr	Val	Lys	New CPV-2a
*CN13/Nevis/2020*	Leu	Thr	Ala	Phe	Ala	Gly	Tyr	Asn	Tyr	Asp	Asp	Asn	Thr	Val	Lys	New CPV-2a
*CN14/Nevis/2020*	Leu	Thr	Ala	Phe	Ala	Gly	Tyr	Asn	Tyr	Asn	Asp	Asn	Thr	Val	Lys	New CPV-2a
*CN15/Nevis/2020*	Leu	Thr	Ala	Phe	Ala	Gly	Tyr	Asn	Tyr	Asp	Asp	Asn	Thr	Val	Lys	New CPV-2a
*CN16/Nevis/2020*	Leu	Thr	Ala	Phe	Ala	Gly	Tyr	Asn	Tyr	Asp	Asp	Asn	Thr	Val	Lys	New CPV-2a
*CN17/Nevis/2020*	Leu	Thr	Ala	Phe	Ala	Gly	Tyr	Asn	Tyr	Asp	Asp	Asn	Thr	Val	Lys	New CPV-2a
*CN18/Nevis/2020*	Leu	Thr	Ala	Phe	Ala	Gly	Tyr	Asn	Tyr	Asp	Asp	Asn	Thr	Val	Lys	New CPV-2a
*CN19/Nevis/2020*	Leu	Thr	Thr	Phe	Ala	Gly	Tyr	Asn	Tyr	Asp	Asp	Asn	Thr	Val	Lys	New CPV-2a
*CN20/Nevis/2020*	Leu	Thr	Thr	Phe	Ala	Gly	Tyr	Asn	Tyr	Asp	Asp	Asn	Thr	Val	Lys	New CPV-2a
*CN21/Nevis/2020*	Leu	Thr	Ala	Phe	Ala	Gly	Tyr	Asn	Tyr	Asp	Asp	Asn	Thr	Val	Lys	New CPV-2a
*CN22/Nevis/2020*	Leu	Thr	Ala	Phe	Ala	Gly	Tyr	Asn	Tyr	Asp	Asp	Asn	Thr	Val	Lys	New CPV-2a
*CN23/Nevis/2020*	Leu	Thr	Ala	Phe	Ala	Gly	Tyr	Asn	Tyr	Asn	Asp	Asn	Thr	Val	Lys	New CPV-2a
*CN24/Nevis/2020*	Leu	Thr	Thr	Phe	Ala	Gly	Tyr	Asn	Tyr	Asp	Asp	Asn	Thr	Val	Lys	New CPV-2a
*CN25/Nevis/2020*	Leu	Thr	Ala	Phe	Ala	Gly	Tyr	Asn	Tyr	Asn	Asp	Asn	Thr	Val	Lys	New CPV-2a
*CN26/Nevis/2020*	Leu	Thr	Ala	Phe	Ala	Gly	Tyr	Asn	Tyr	Asp	Asp	Asn	Thr	Val	Lys	New CPV-2a
*CN27/Nevis/2020* ^1^	Leu	Thr	Thr	Phe	Ala	Gly	Tyr	Asn	Tyr	Asp	Asp	Asn	Thr	Val	Lys	New CPV-2a
*CN28/Nevis/2020*	Leu	Thr	Ala	Phe	Ala	Gly	Tyr	Asn	Tyr	Asp	Asp	Asn	Thr	Val	Lys	New CPV-2a
*CN29/Nevis/2020*	Leu	Thr	Ala	Phe	Ala	Gly	Tyr	Asn	Tyr	Asp	Asp	Asn	Thr	Val	Lys	New CPV-2a
*CN30/Nevis/2020*	Leu	Thr	Ala	Phe	Ala	Gly	Tyr	Asn	Tyr	Asp	Asp	Asn	Thr	Val	Lys	New CPV-2a
*CN31/Nevis/2020*	Leu	Thr	Ala	Phe	Ala	Gly	Tyr	Asn	Tyr	Asp	Asp	Asn	Thr	Val	Lys	New CPV-2a
*CN32/Nevis/2020*	Leu	Thr	Ala	Phe	Ala	Gly	Tyr	Asn	Tyr	Asp	Asp	Asn	Thr	Val	Lys	New CPV-2a
*CN33/Nevis/2020*	Leu	Thr	Ala	Phe	Ala	Gly	Tyr	Asn	Tyr	Asp	Asp	Asn	Thr	Val	Lys	New CPV-2a
*CN34/Nevis/2020*	Leu	Thr	Ala	Phe	Ala	Gly	Tyr	Asn	Tyr	Asp	Asp	Asn	Thr	Val	Lys	New CPV-2a
*CN35/Nevis/2020*	Leu	Thr	Ala	Phe	Ala	Gly	Tyr	Asn	Tyr	Asp	Asp	Asn	Thr	Val	Lys	New CPV-2a
*CN36/Nevis/2020*	Leu	Thr	Ala	Phe	Ala	Gly	Tyr	Asn	Tyr	Asp	Asp	Asn	Thr	Val	Lys	New CPV-2a
*CN37/Nevis/2020*	Leu	Thr	Ala	Phe	Ala	Gly	Tyr	Asn	Tyr	Asp	Asp	Asn	Thr	Val	Lys	New CPV-2a
*CN38/Nevis/2020*	Leu	Thr	Ala	Phe	Ala	Gly	Tyr	Asn	Tyr	Asp	Asp	Asn	Thr	Val	Lys	New CPV-2a
*CN39/Nevis/2020*	Leu	Thr	Ala	Phe	Ala	Gly	Tyr	Asn	Tyr	Asp	Asp	Asn	Thr	Val	Lys	New CPV-2a
*CN40/Nevis/2020* ^1^	Leu	Thr	Ala	Phe	Ala	Gly	Tyr	Asn	Tyr	Asp	Asp	Asn	Thr	Val	Lys	New CPV-2a
VANGUARD/vaccine	Arg	Ile	Ala	Phe	Ser	Ala	Asp	Asn	Tyr	Asp	Glu	Asn	Thr	Val	Lys	CPV-2
Duramune/vaccine	Leu	Thr	Ala	Phe	Ala	Gly	Tyr	Lys	Tyr	Asp	Asp	Asp	Thr	Val	Glu	New CPV-2b

^1^ Strain CN27 and strain CN40 were detected in the same dog on 5 September 2020 and 5 November 2020, respectively.

**Table 3:** Nucleotide (nt) mismatches (highlighted with yellow) between the nearly complete genome sequences (4269 nt, spanning all the coding regions) of canine parvovirus-2 (CPV-2) strain CPV12 (representing the earliest CPV-2 strains from the late 1970s) and CPV-2 strains detected on St. Kitts (strain RVC50) and Nevis (strains CN10, CN14, and CN20) islands. Nonsynonymous mutations that differed in an nt from the CPV12 sequence are shown in red. Alignment of the nearly full-length nt and complete deduced amino acid sequences of the CPV-2 strains from St. Kitts and Nevis with those of strain CPV12 are shown in Supplementary Materials S5 and S6, respectively.

Nt Position ^1^	CPV-2 Strain (Year/Place of Detection)					Nt Change → Translational Effect	Coding Region
	CPV-12 (1978/USA)	RVC50 (2016/St. Kitts)	CN10 (2020/Nevis)	CN14 (2020/Nevis)	CN20 (2020/Nevis)		
516	G	A	A	A	A	G516A → Synonymous	Within the NS coding region
562	T	C	C	C	C	T562C → Synonymous	
726	G	G	A	G	A	G726A → Synonymous	
753	A	A	G	A	G	A753G ^2^ → Synonymous	
1104	T	T	C	T	C	T1104C ^2^ → Synonymous	
1164	A	A	G	A	G	A1164G → Synonymous	
1209	T	C	C	C	C	T1209C → Synonymous	
1305	T	C	C	C	C	T1305C ^2^ → Synonymous	
1623	A	G	G	A	G	A1623G → Synonymous	
1752	A	G	G	G	G	A1752G → NS2 Thr94Ala	
1923	G	A	A	A	A	G1923A → NS2 Asp151Asn	
1926	A	G	G	G	G	A1926G → NS2 Met152Val	
1975	T	C	C	C	C	T1975C → Synonymous	
2086	A	G	G	G	G	A2086G → VP1 Intron	
2154	G	A	A	A	A	G2154A → Synonymous	Within the VP coding region
2436	A	A	G	A	G	A2436G ^2^ → Synonymous	
2574	T	A	A	A	A	T2754A → Synonymous	
2773	A	T	T	T	T	A2773T → VP2 Met87Leu ^3^	
2816	T	C	C	C	C	T2816C → VP2 Ile101Thr ^3^	
2923	G	A	A	A	A	G2923A → Synonymous	
2940	A	G	G	G	G	A2940G → Synonymous	
3006	C	T	T	T	T	C3006T → Synonymous	
3039	T	T	G	T	T	T3039G ^2^ → Synonymous	
3117	A	A	G	A	A	A3117G → Synonymous	
3297	G	G	G	G	A	G3297A → VP2 Ala262Thr	
3403	T	G	G	G	G	T3403G → VP2 Ser297Ala	
3413	C	G	G	G	G	C3413G → VP2 Ala300Gly ^3^	
3427	G	T	T	T	T	G3427T → VP2 Asp305Tyr ^3^	
3582	T	C	C	C	C	T3582C → Synonymous	
3631	G	G	G	A	G	G3631A ^2^ → VP2 Asp373Asn	
3637	A	G	G	G	G	A3637G → VP2 Asn375Asp ^3^	
3702	G	G	G	A	G	G3702A → Synonymous	
3894	A	G	G	G	G	A3894G → Synonymous	
4017	A	G	G	G	G	A4017G → Synonymous	
4030	T	C	C	C	C	T4030C → Synonymous	

^1^ Nucleotide positions are those of the nearly complete genome sequence of strain CPV12 (GenBank accession number MN451655); ^2^ Present in <10 published CPV-2 sequences, as revealed by BLASTN analysis (https://blast.ncbi.nlm.nih.gov/, accessed on 9 April 2021) and multiple alignments of the dataset of 210 nearly complete CPV-2 sequences; ^3^ Nucleotide substitutions (and corresponding amino acid changes) that emerged during the global sweep from CPV-2 to CPV-2a [16].

**Table 4:** Evolutionarily relevant amino acid residues in canine parvovirus-2 (CPV-2) protein sequences, as described in previous studies [21,44]. The two nonsynonymous substitutions (VP2 Ala262Thr and VP2 Asp373Asn) observed among the CPV-2 strains from St. Kitts and Nevis are also shown.

*Amino Acid Residue*	NS1						NS2		VP2				
	60	544	545	572	630		152		262	267	324	373	426
Clade ^1^													
CPV-2 origin	Ile	Tyr	Glu	Glu	Leu		Met		Ala	Phe	Tyr	Asp	Asn
Asian	Ile/Val	Phe/Tyr	Glu/Val	Lys	Leu/Pro		Val		Ala	Phe/Tyr	Ile/Tyr	Asp	Asn/Asp/Glu
Western	Ile	Phe/Tyr	Glu	Glu	Leu		Met/Val		Ala	Phe	Leu/Tyr	Asp	Asn/Asp/Glu
Strain/Place/Year													
CN10/Nevis/2020	Ile	Tyr	Glu	Glu	Leu		Val		Ala	Phe	Tyr	Asp	Asn
CN14/Nevis/2020	Ile	Tyr	Glu	Glu	Leu		Val		Ala	Phe	Tyr	Asn ^3^	Asn
CN20/Nevis/2020	Ile	Tyr	Glu	Glu	Leu		Val		Thr ^2^	Phe	Tyr	Asp	Asn
RVC50/St. Kitts/2016	Ile	Tyr	Glu	Glu	Leu		Val		Ala	Phe	Tyr	Asp	Asn

^1^ As described in previous studies [21,44]. Western: CPV-2 strains from Europe and the Americas; ^2^ Reported in new CPV-2a and new CPV-2b strains from Western Australia [38], 2 new CPV-2a strains from India (GenBank accession numbers DQ182624 and KU866399), and a single new CPV-2a strain from China (MH177301); ^3^ Found in 2 new CPV-2a strains (one each from Australia (MN259033) and Thailand (GQ379047)), a single CPV-2 strain from a cat in Taiwan (KY010491), and 2 feline panleukopenia virus strains (MH559110 and MK570710).

## Supplementary Materials

The following are available online at https://www.mdpi.com/article/10.3390/v13061083/s1, Supplementary Material S1: Primers used in the present study, Supplementary Material S2: Multiple alignments of the putative VP2 proteins of canine parvovirus-2 (CPV-2) strains detected in Nevis (designated with prefix CN) with those reported from St. Kitts in 2016 (strain RVC50) and in 2020 (strains CK81 and CK84), Supplementary Material S3: Multiple alignments of the VP2 coding sequences of canine parvovirus-2 (CPV-2) strains detected in Nevis (designated with prefix CN) during 2020 with those reported from the neighboring island of St. Kitts in 2016 (strain RVC50) and in 2020 (strains CK81 and CK84), Supplementary Material S4: Gross (A) and microscopic (B) lesions observed in the small intestine of a dog with hemorrhagic gastroenteritis that eventually died on the island of St. Kitts. Small intestinal scrapings collected during necropsy tested positive for canine parvovirus-2 (CPV-2) by PCR. Analysis of the complete deduced VP2 amino acid sequence identified the CPV-2 strain as new CPV-2a, Supplementary Material S5: Nucleotide (nt) mismatches between the nearly complete genome sequences (4269 nt, spanning all the coding regions) of canine parvovirus-2 (CPV-2) strain CPV12 (representing the earliest CPV-2 strains from the late 1970s) and CPV-2 strains detected on St. Kitts in 2016 (strain RVC50) and Nevis in 2020 (strains CN10, CN14, and CN20), Supplementary Material S6: Multiple alignments of the putative NS1 (A), NS2 (B), VP1 (C) and VP2 (D) proteins of canine parvovirus-2 (CPV-2) strain CPV12 (GenBank accession number MN451655, representing the earliest CPV-2 strains from the late 1970s) and CPV-2 strains detected on St. Kitts in 2016 (strain RVC50) and Nevis in 2020 (strains CN10, CN14, and CN20), Supplementary Material S7: Phylogenetic analysis of the nearly complete genomes of canine parvovirus-2 (CPV-2) strains from St. Kitts and Nevis with those of other CPV-2 strains. The tree was created using the Hasegawa-Kishino-Yano model with gamma-distributed rate variation among sites and 1000 bootstrap replicates, Supplementary Material S8: Phylogenetic analysis of the nearly complete genomes of canine parvovirus-2 (CPV-2) strains from St. Kitts and Nevis with those of other CPV-2 strains. The tree was created using the General Time Reversible model with gamma-distributed rate variation among sites and 1000 bootstrap replicates, Supplementary Material S9: Phylogenetic analysis of the complete VP2 coding sequences of canine parvovirus-2 (CPV-2) strains from St. Kitts and Nevis with those of other CPV-2 strains.
