# Supplementary material for: Molecular Investigation of Canine Parvovirus-2 (CPV-2) Outbreak in Nevis Island: Analysis of the Nearly Complete Genomes of CPV-2 Strains from the Caribbean Region
Source: Viruses. 2021 Jun 6;13(6):1083. doi: 10.3390/v13061083 (PMC8227521; doi:10.3390/v13061083)
Supplement: Supplementary file 1 [file viruses-13-01083-s001.zip › Supplementary materials_R1/Supplementary material S1.pdf]

Supplementary material S1. Primers used in the present study. Five primer pairs (highlighted with green) were employed in overlapping PCRs to amplify the nearly complete genomes of the canine parvovirus-2 (CPV-2) strains from St. Kitts and Nevis. PCR primers as well as forward and reverse internal primers were used for obtaining the CPV-2 sequences. The internal primers are also shown in the table. Overlapping PCR reactions -4 and -5 amplified the complete VP2 ORF of CPV-2 strains. PCR reaction-4 was used as the screening assay.

PCR reaction	Primer name	Primer sequence (5'-3') [1]	Primer position [2]
**1**	CPV2-1F	ATG TCT GGC AAC CAG TAT ACT GAG	nt 272-nt 295
	CPV2-1160R	CTC TTT TAC TAA CCA AGT CCC GC	nt 1160-nt 1138
	CPV2-324F	AGA AAC ATG CAG AAA ATG AAG C	nt 324-nt 345
	CPV2-768R	TTT ACC GAA CAA AGA GTC ACC	nt 768-nt 748
	CPV2-807F	CAG AAG ATA GTG AAT GGG TGA C	nt 807-nt 828
	CPV2-1136R	AAG TAC ATT TGA TTG ACA CTT CC	nt 1136-nt 1114
**2**	CPV2-929F	GTC CAC ATG ACA AAA GAA AGT GGC	nt 929-nt 952
	CPV2-1970R	CCG CCC AGT TTT CAT CCC ATT C	nt 1970-nt 1949
	CPV2-961F	AAG TAC TGA TTC TGG TTG GAA A	nt 961-nt 981
	CPV2-1424R	CAC ATG CTA TAG CGT GAC AAA C	nt 1424-nt 1403
	CPV2-1465F	TCA TGG ACC AGC AAG TAC AGG	nt 1465-nt 1485
	CPV2-1933R	ATG ATG TGT ATA GTT AGC CAT GG	nt 1933-nt 1911
**3**	CPV2-1772F	CCT GAA CAT ACA CAA CCA ATA AGA G	nt 1772-nt 1796
	CPV2-2921R	TGA AAG TAC CCG TAG AAA TCC CC	nt 2921-nt 2899
	CPV2-1820F	GTA TGT AAG CTT CCA GGA GAC	nt 1820-nt 1840
	CPV2-2304R	CTC TTT GCC GGA GGT GCC ATC	nt 2304-nt 2284
	CPV2-2399F	CCA GGT TAT AAA TAT CTT GGG CC	nt 2399-nt 2421
	CPV2-2870R	CTC CAG ACC CGT TCC CAG ATC	nt 2870-nt 2850
**4**	CPV2-V2-2786F	ATG AGT GAT GGA GCA GTT CAA CC	nt 2786-nt 2808
	CPV2-V2-3726R	CGT CTT TTA TCT TGT TGA ACT CC	nt 3726-nt 3704
	CPV2-V2-2816F	GGT CAA CCT GCT GTC AGA AAT G	nt 2816-nt 2837
	CPV2-V2-3093R	GCA TGA GTA TCA TCT AAA GCC ATG	nt 3093-nt 3070
**5**	CPV2-V2-3465F	GCA CAC CAA CAA ATA TAT ACC ATG G	nt 3465-nt 3489
	CPV2-V2-4585R	GGT GCT AGT TGA TAT GTA ATA AAC A	nt 4585-nt 4561
	CPV2-V2-4151F	GCA TTA AAT AAT GTG CCA CCA G	nt 4151-nt 4172
	CPV2-V2-4378R	ACC TTT CCA CCA AAA ATC TGA G	nt 4378-nt 4357

[1] The primer sequences were designed from conserved regions following multiple alignment of the complete/nearly complete genomes of several CPV-2 strains representing the different CPV-2 variants (CPV-2, CPV-2a, CPV-2b, CPV-2c, new CPV-2a and new CPV-2b strains).

[2] Nucleotide (nt) position corresponds to that of reference CPV-2 strain CPV-N, GenBank accession number M19296.
