# Supplementary material for: Molecular Investigation of Canine Parvovirus-2 (CPV-2) Outbreak in Nevis Island: Analysis of the Nearly Complete Genomes of CPV-2 Strains from the Caribbean Region
Source: Viruses. 2021 Jun 6;13(6):1083. doi: 10.3390/v13061083 (PMC8227521; doi:10.3390/v13061083)
Supplement: Supplementary file 1 [file viruses-13-01083-s001.zip › Supplementary materials_R1/Supplementary material S2.pdf]

Supplementary material S2. Multiple alignment of the putative VP2 proteins of canine parvovirus-2 (CPV-2) strains detected in Nevis (designated with prefix CN) with those reported from St. Kitts in 2016 (strain RVC50) and in 2020 (strains CK81 and CK84). Amino acid (aa) mismatches are shown with red. A '*' denotes an identical aa residue. Numbers to the right indicate the positions of the aa for respective CPV-2 strains.

```
CN7_VP2         MSDGAVQPDGGQPAVRNERATGSGNGSGGGGGGGSGGVGISTGTFNNQTEFKFLENGWVE 60
CN8_VP2         MSDGAVQPDGGQPAVRNERATGSGNGSGGGGGGGSGGVGISTGTFNNQTEFKFLENGWVE 60
CN10_VP2        MSDGAVQPDGGQPAVRNERATGSGNGSGGGGGGGSGGVGISTGTFNNQTEFKFLENGWVE 60
CN11_VP2        MSDGAVQPDGGQPAVRNERATGSGNGSGGGGGGGSGGVGISTGTFNNQTEFKFLENGWVE 60
CN12_VP2        MSDGAVQPDGGQPAVRNERATGSGNGSGGGGGGGSGGVGISTGTFNNQTEFKFLENGWVE 60
CN13_VP2        MSDGAVQPDGGQPAVRNERATGSGNGSGGGGGGGSGGVGISTGTFNNQTEFKFLENGWVE 60
CN14_VP2        MSDGAVQPDGGQPAVRNERATGSGNGSGGGGGGGSGGVGISTGTFNNQTEFKFLENGWVE 60
CN15_VP2        MSDGAVQPDGGQPAVRNERATGSGNGSGGGGGGGSGGVGISTGTFNNQTEFKFLENGWVE 60
CN16_VP2        MSDGAVQPDGGQPAVRNERATGSGNGSGGGGGGGSGGVGISTGTFNNQTEFKFLENGWVE 60
CN17_VP2        MSDGAVQPDGGQPAVRNERATGSGNGSGGGGGGGSGGVGISTGTFNNQTEFKFLENGWVE 60
CN18_VP2        MSDGAVQPDGGQPAVRNERATGSGNGSGGGGGGGSGGVGISTGTFNNQTEFKFLENGWVE 60
CN19_VP2        MSDGAVQPDGGQPAVRNERATGSGNGSGGGGGGGSGGVGISTGTFNNQTEFKFLENGWVE 60
CN20_VP2        MSDGAVQPDGGQPAVRNERATGSGNGSGGGGGGGSGGVGISTGTFNNQTEFKFLENGWVE 60
CN21_VP2        MSDGAVQPDGGQPAVRNERATGSGNGSGGGGGGGSGGVGISTGTFNNQTEFKFLENGWVE 60
CN22_VP2        MSDGAVQPDGGQPAVRNERATGSGNGSGGGGGGGSGGVGISTGTFNNQTEFKFLENGWVE 60
CN23_VP2        MSDGAVQPDGGQPAVRNERATGSGNGSGGGGGGGSGGVGISTGTFNNQTEFKFLENGWVE 60
CN24_VP2        MSDGAVQPDGGQPAVRNERATGSGNGSGGGGGGGSGGVGISTGTFNNQTEFKFLENGWVE 60
CN25_VP2        MSDGAVQPDGGQPAVRNERATGSGNGSGGGGGGGSGGVGISTGTFNNQTEFKFLENGWVE 60
CN26_VP2        MSDGAVQPDGGQPAVRNERATGSGNGSGGGGGGGSGGVGISTGTFNNQTEFKFLENGWVE 60
CN27_VP2        MSDGAVQPDGGQPAVRNERATGSGNGSGGGGGGGSGGVGISTGTFNNQTEFKFLENGWVE 60
CN28_VP2        MSDGAVQPDGGQPAVRNERATGSGNGSGGGGGGGSGGVGISTGTFNNQTEFKFLENGWVE 60
CN29_VP2        MSDGAVQPDGGQPAVRNERATGSGNGSGGGGGGGSGGVGISTGTFNNQTEFKFLENGWVE 60
CN30_VP2        MSDGAVQPDGGQPAVRNERATGSGNGSGGGGGGGSGGVGISTGTFNNQTEFKFLENGWVE 60
CN31_VP2        MSDGAVQPDGGQPAVRNERATGSGNGSGGGGGGGSGGVGISTGTFNNQTEFKFLENGWVE 60
CN32_VP2        MSDGAVQPDGGQPAVRNERATGSGNGSGGGGGGGSGGVGISTGTFNNQTEFKFLENGWVE 60
CN33_VP2        MSDGAVQPDGGQPAVRNERATGSGNGSGGGGGGGSGGVGISTGTFNNQTEFKFLENGWVE 60
CN34_VP2        MSDGAVQPDGGQPAVRNERATGSGNGSGGGGGGGSGGVGISTGTFNNQTEFKFLENGWVE 60
CN35_VP2        MSDGAVQPDGGQPAVRNERATGSGNGSGGGGGGGSGGVGISTGTFNNQTEFKFLENGWVE 60
CN36_VP2        MSDGAVQPDGGQPAVRNERATGSGNGSGGGGGGGSGGVGISTGTFNNQTEFKFLENGWVE 60
CN37_VP2        MSDGAVQPDGGQPAVRNERATGSGNGSGGGGGGGSGGVGISTGTFNNQTEFKFLENGWVE 60
CN38_VP2        MSDGAVQPDGGQPAVRNERATGSGNGSGGGGGGGSGGVGISTGTFNNQTEFKFLENGWVE 60
CN39_VP2        MSDGAVQPDGGQPAVRNERATGSGNGSGGGGGGGSGGVGISTGTFNNQTEFKFLENGWVE 60
CN40_VP2        MSDGAVQPDGGQPAVRNERATGSGNGSGGGGGGGSGGVGISTGTFNNQTEFKFLENGWVE 60
CK81_VP2        MSDGAVQPDGGQPAVRNERATGSGNGSGGGGGGGSGGVGISTGTFNNQTEFKFLENGWVE 60
CK84_VP2        MSDGAVQPDGGQPAVRNERATGSGNGSGGGGGGGSGGVGISTGTFNNQTEFKFLENGWVE 60
RVC50_VP2       MSDGAVQPDGGQPAVRNERATGSGNGSGGGGGGGSGGVGISTGTFNNQTEFKFLENGWVE 60
                ************************************************************

CN7_VP2         ITANSSRLVHLNMPESENYRRVVVNNLDKTAVNGNMALDDTHAQIVTPWSLVDANAWGVW 120
CN8_VP2         ITANSSRLVHLNMPESENYRRVVVNNLDKTAVNGNMALDDTHAQIVTPWSLVDANAWGVW 120
CN10_VP2        ITANSSRLVHLNMPESENYRRVVVNNLDKTAVNGNMALDDTHAQIVTPWSLVDANAWGVW 120
CN11_VP2        ITANSSRLVHLNMPESENYRRVVVNNLDKTAVNGNMALDDTHAQIVTPWSLVDANAWGVW 120
CN12_VP2        ITANSSRLVHLNMPESENYRRVVVNNLDKTAVNGNMALDDTHAQIVTPWSLVDANAWGVW 120
CN13_VP2        ITANSSRLVHLNMPESENYRRVVVNNLDKTAVNGNMALDDTHAQIVTPWSLVDANAWGVW 120
CN14_VP2        ITANSSRLVHLNMPESENYRRVVVNNLDKTAVNGNMALDDTHAQIVTPWSLVDANAWGVW 120
CN15_VP2        ITANSSRLVHLNMPESENYRRVVVNNLDKTAVNGNMALDDTHAQIVTPWSLVDANAWGVW 120
CN16_VP2        ITANSSRLVHLNMPESENYRRVVVNNLDKTAVNGNMALDDTHAQIVTPWSLVDANAWGVW 120
CN17_VP2        ITANSSRLVHLNMPESENYRRVVVNNLDKTAVNGNMALDDTHAQIVTPWSLVDANAWGVW 120
CN18_VP2        ITANSSRLVHLNMPESENYRRVVVNNLDKTAVNGNMALDDTHAQIVTPWSLVDANAWGVW 120
CN19_VP2        ITANSSRLVHLNMPESENYRRVVVNNLDKTAVNGNMALDDTHAQIVTPWSLVDANAWGVW 120
CN20_VP2        ITANSSRLVHLNMPESENYRRVVVNNLDKTAVNGNMALDDTHAQIVTPWSLVDANAWGVW 120
CN21_VP2        ITANSSRLVHLNMPESENYRRVVVNNLDKTAVNGNMALDDTHAQIVTPWSLVDANAWGVW 120
CN22_VP2        ITANSSRLVHLNMPESENYRRVVVNNLDKTAVNGNMALDDTHAQIVTPWSLVDANAWGVW 120
CN23_VP2        ITANSSRLVHLNMPESENYRRVVVNNLDKTAVNGNMALDDTHAQIVTPWSLVDANAWGVW 120
CN24_VP2        ITANSSRLVHLNMPESENYRRVVVNNLDKTAVNGNMALDDTHAQIVTPWSLVDANAWGVW 120
CN25_VP2        ITANSSRLVHLNMPESENYRRVVVNNLDKTAVNGNMALDDTHAQIVTPWSLVDANAWGVW 120
CN26_VP2        ITANSSRLVHLNMPESENYRRVVVNNLDKTAVNGNMALDDTHAQIVTPWSLVDANAWGVW 120
CN27_VP2        ITANSSRLVHLNMPESENYRRVVVNNLDKTAVNGNMALDDTHAQIVTPWSLVDANAWGVW 120
CN28_VP2        ITANSSRLVHLNMPESENYRRVVVNNLDKTAVNGNMALDDTHAQIVTPWSLVDANAWGVW 120
CN29_VP2        ITANSSRLVHLNMPESENYRRVVVNNLDKTAVNGNMALDDTHAQIVTPWSLVDANAWGVW 120
CN30_VP2        ITANSSRLVHLNMPESENYRRVVVNNLDKTAVNGNMALDDTHAQIVTPWSLVDANAWGVW 120
CN31_VP2        ITANSSRLVHLNMPESENYRRVVVNNLDKTAVNGNMALDDTHAQIVTPWSLVDANAWGVW 120
CN32_VP2        ITANSSRLVHLNMPESENYRRVVVNNLDKTAVNGNMALDDTHAQIVTPWSLVDANAWGVW 120
CN33_VP2        ITANSSRLVHLNMPESENYRRVVVNNLDKTAVNGNMALDDTHAQIVTPWSLVDANAWGVW 120
CN34_VP2        ITANSSRLVHLNMPESENYRRVVVNNLDKTAVNGNMALDDTHAQIVTPWSLVDANAWGVW 120
CN35_VP2        ITANSSRLVHLNMPESENYRRVVVNNLDKTAVNGNMALDDTHAQIVTPWSLVDANAWGVW 120
CN36_VP2        ITANSSRLVHLNMPESENYRRVVVNNLDKTAVNGNMALDDTHAQIVTPWSLVDANAWGVW 120
CN37_VP2        ITANSSRLVHLNMPESENYRRVVVNNLDKTAVNGNMALDDTHAQIVTPWSLVDANAWGVW 120
CN38_VP2        ITANSSRLVHLNMPESENYRRVVVNNLDKTAVNGNMALDDTHAQIVTPWSLVDANAWGVW 120
CN39_VP2        ITANSSRLVHLNMPESENYRRVVVNNLDKTAVNGNMALDDTHAQIVTPWSLVDANAWGVW 120
CN40_VP2        ITANSSRLVHLNMPESENYRRVVVNNLDKTAVNGNMALDDTHAQIVTPWSLVDANAWGVW 120
CK81_VP2        ITANSSRLVHLNMPESENYRRVVVNNLDKTAVNGNMALDDTHAQIVTPWSLVDANAWGVW 120
CK84_VP2        ITANSSRLVHLNMPESENYRRVVVNNLDKTAVNGNMALDDTHAQIVTPWSLVDANAWGVW 120
RVC50_VP2       ITANSSRLVHLNMPESENYRRVVVNNLDKTAVNGNMALDDTHAQIVTPWSLVDANAWGVW 120
                ************************************************************
```

```
CN7_VP2         FNPGDWQLIVNTMSELHLVSFEQEIFNVVLKTVSESATQPPTKVYNNDLTASLMVALDSN 180
CN8_VP2         FNPGDWQLIVNTMSELHLVSFEQEIFNVVLKTVSESATQPPTKVYNNDLTASLMVALDSN 180
CN10_VP2        FNPGDWQLIVNTMSELHLVSFEQEIFNVVLKTVSESATQPPTKVYNNDLTASLMVALDSN 180
CN11_VP2        FNPGDWQLIVNTMSELHLVSFEQEIFNVVLKTVSESATQPPTKVYNNDLTASLMVALDSN 180
CN12_VP2        FNPGDWQLIVNTMSELHLVSFEQEIFNVVLKTVSESATQPPTKVYNNDLTASLMVALDSN 180
CN13_VP2        FNPGDWQLIVNTMSELHLVSFEQEIFNVVLKTVSESATQPPTKVYNNDLTASLMVALDSN 180
CN14_VP2        FNPGDWQLIVNTMSELHLVSFEQEIFNVVLKTVSESATQPPTKVYNNDLTASLMVALDSN 180
CN15_VP2        FNPGDWQLIVNTMSELHLVSFEQEIFNVVLKTVSESATQPPTKVYNNDLTASLMVALDSN 180
CN16_VP2        FNPGDWQLIVNTMSELHLVSFEQEIFNVVLKTVSESATQPPTKVYNNDLTASLMVALDSN 180
CN17_VP2        FNPGDWQLIVNTMSELHLVSFEQEIFNVVLKTVSESATQPPTKVYNNDLTASLMVALDSN 180
CN18_VP2        FNPGDWQLIVNTMSELHLVSFEQEIFNVVLKTVSESATQPPTKVYNNDLTASLMVALDSN 180
CN19_VP2        FNPGDWQLIVNTMSELHLVSFEQEIFNVVLKTVSESATQPPTKVYNNDLTASLMVALDSN 180
CN20_VP2        FNPGDWQLIVNTMSELHLVSFEQEIFNVVLKTVSESATQPPTKVYNNDLTASLMVALDSN 180
CN21_VP2        FNPGDWQLIVNTMSELHLVSFEQEIFNVVLKTVSESATQPPTKVYNNDLTASLMVALDSN 180
CN22_VP2        FNPGDWQLIVNTMSELHLVSFEQEIFNVVLKTVSESATQPPTKVYNNDLTASLMVALDSN 180
CN23_VP2        FNPGDWQLIVNTMSELHLVSFEQEIFNVVLKTVSESATQPPTKVYNNDLTASLMVALDSN 180
CN24_VP2        FNPGDWQLIVNTMSELHLVSFEQEIFNVVLKTVSESATQPPTKVYNNDLTASLMVALDSN 180
CN25_VP2        FNPGDWQLIVNTMSELHLVSFEQEIFNVVLKTVSESATQPPTKVYNNDLTASLMVALDSN 180
CN26_VP2        FNPGDWQLIVNTMSELHLVSFEQEIFNVVLKTVSESATQPPTKVYNNDLTASLMVALDSN 180
CN27_VP2        FNPGDWQLIVNTMSELHLVSFEQEIFNVVLKTVSESATQPPTKVYNNDLTASLMVALDSN 180
CN28_VP2        FNPGDWQLIVNTMSELHLVSFEQEIFNVVLKTVSESATQPPTKVYNNDLTASLMVALDSN 180
CN29_VP2        FNPGDWQLIVNTMSELHLVSFEQEIFNVVLKTVSESATQPPTKVYNNDLTASLMVALDSN 180
CN30_VP2        FNPGDWQLIVNTMSELHLVSFEQEIFNVVLKTVSESATQPPTKVYNNDLTASLMVALDSN 180
CN31_VP2        FNPGDWQLIVNTMSELHLVSFEQEIFNVVLKTVSESATQPPTKVYNNDLTASLMVALDSN 180
CN32_VP2        FNPGDWQLIVNTMSELHLVSFEQEIFNVVLKTVSESATQPPTKVYNNDLTASLMVALDSN 180
CN33_VP2        FNPGDWQLIVNTMSELHLVSFEQEIFNVVLKTVSESATQPPTKVYNNDLTASLMVALDSN 180
CN34_VP2        FNPGDWQLIVNTMSELHLVSFEQEIFNVVLKTVSESATQPPTKVYNNDLTASLMVALDSN 180
CN35_VP2        FNPGDWQLIVNTMSELHLVSFEQEIFNVVLKTVSESATQPPTKVYNNDLTASLMVALDSN 180
CN36_VP2        FNPGDWQLIVNTMSELHLVSFEQEIFNVVLKTVSESATQPPTKVYNNDLTASLMVALDSN 180
CN37_VP2        FNPGDWQLIVNTMSELHLVSFEQEIFNVVLKTVSESATQPPTKVYNNDLTASLMVALDSN 180
CN38_VP2        FNPGDWQLIVNTMSELHLVSFEQEIFNVVLKTVSESATQPPTKVYNNDLTASLMVALDSN 180
CN39_VP2        FNPGDWQLIVNTMSELHLVSFEQEIFNVVLKTVSESATQPPTKVYNNDLTASLMVALDSN 180
CN40_VP2        FNPGDWQLIVNTMSELHLVSFEQEIFNVVLKTVSESATQPPTKVYNNDLTASLMVALDSN 180
CK81_VP2        FNPGDWQLIVNTMSELHLVSFEQEIFNVVLKTVSESATQPPTKVYNNDLTASLMVALDSN 180
CK84_VP2        FNPGDWQLIVNTMSELHLVSFEQEIFNVVLKTVSESATQPPTKVYNNDLTASLMVALDSN 180
RVC50_VP2       FNPGDWQLIVNTMSELHLVSFEQEIFNVVLKTVSESATQPPTKVYNNDLTASLMVALDSN 180
                ************************************************************
```

```
CN7_VP2         NTMPFTPAAMRSETLGFYPWKPTIPTPWRYYFQWDRTLIPSHTGTSGTPTNIYHGTDPDD 240
CN8_VP2         NTMPFTPAAMRSETLGFYPWKPTIPTPWRYYFQWDRTLIPSHTGTSGTPTNIYHGTDPDD 240
CN10_VP2        NTMPFTPAAMRSETLGFYPWKPTIPTPWRYYFQWDRTLIPSHTGTSGTPTNIYHGTDPDD 240
CN11_VP2        NTMPFTPAAMRSETLGFYPWKPTIPTPWRYYFQWDRTLIPSHTGTSGTPTNIYHGTDPDD 240
CN12_VP2        NTMPFTPAAMRSETLGFYPWKPTIPTPWRYYFQWDRTLIPSHTGTSGTPTNIYHGTDPDD 240
CN13_VP2        NTMPFTPAAMRSETLGFYPWKPTIPTPWRYYFQWDRTLIPSHTGTSGTPTNIYHGTDPDD 240
CN14_VP2        NTMPFTPAAMRSETLGFYPWKPTIPTPWRYYFQWDRTLIPSHTGTSGTPTNIYHGTDPDD 240
CN15_VP2        NTMPFTPAAMRSETLGFYPWKPTIPTPWRYYFQWDRTLIPSHTGTSGTPTNIYHGTDPDD 240
CN16_VP2        NTMPFTPAAMRSETLGFYPWKPTIPTPWRYYFQWDRTLIPSHTGTSGTPTNIYHGTDPDD 240
CN17_VP2        NTMPFTPAAMRSETLGFYPWKPTIPTPWRYYFQWDRTLIPSHTGTSGTPTNIYHGTDPDD 240
CN18_VP2        NTMPFTPAAMRSETLGFYPWKPTIPTPWRYYFQWDRTLIPSHTGTSGTPTNIYHGTDPDD 240
CN19_VP2        NTMPFTPAAMRSETLGFYPWKPTIPTPWRYYFQWDRTLIPSHTGTSGTPTNIYHGTDPDD 240
CN20_VP2        NTMPFTPAAMRSETLGFYPWKPTIPTPWRYYFQWDRTLIPSHTGTSGTPTNIYHGTDPDD 240
CN21_VP2        NTMPFTPAAMRSETLGFYPWKPTIPTPWRYYFQWDRTLIPSHTGTSGTPTNIYHGTDPDD 240
CN22_VP2        NTMPFTPAAMRSETLGFYPWKPTIPTPWRYYFQWDRTLIPSHTGTSGTPTNIYHGTDPDD 240
CN23_VP2        NTMPFTPAAMRSETLGFYPWKPTIPTPWRYYFQWDRTLIPSHTGTSGTPTNIYHGTDPDD 240
CN24_VP2        NTMPFTPAAMRSETLGFYPWKPTIPTPWRYYFQWDRTLIPSHTGTSGTPTNIYHGTDPDD 240
CN25_VP2        NTMPFTPAAMRSETLGFYPWKPTIPTPWRYYFQWDRTLIPSHTGTSGTPTNIYHGTDPDD 240
CN26_VP2        NTMPFTPAAMRSETLGFYPWKPTIPTPWRYYFQWDRTLIPSHTGTSGTPTNIYHGTDPDD 240
CN27_VP2        NTMPFTPAAMRSETLGFYPWKPTIPTPWRYYFQWDRTLIPSHTGTSGTPTNIYHGTDPDD 240
CN28_VP2        NTMPFTPAAMRSETLGFYPWKPTIPTPWRYYFQWDRTLIPSHTGTSGTPTNIYHGTDPDD 240
CN29_VP2        NTMPFTPAAMRSETLGFYPWKPTIPTPWRYYFQWDRTLIPSHTGTSGTPTNIYHGTDPDD 240
CN30_VP2        NTMPFTPAAMRSETLGFYPWKPTIPTPWRYYFQWDRTLIPSHTGTSGTPTNIYHGTDPDD 240
CN31_VP2        NTMPFTPAAMRSETLGFYPWKPTIPTPWRYYFQWDRTLIPSHTGTSGTPTNIYHGTDPDD 240
CN32_VP2        NTMPFTPAAMRSETLGFYPWKPTIPTPWRYYFQWDRTLIPSHTGTSGTPTNIYHGTDPDD 240
CN33_VP2        NTMPFTPAAMRSETLGFYPWKPTIPTPWRYYFQWDRTLIPSHTGTSGTPTNIYHGTDPDD 240
CN34_VP2        NTMPFTPAAMRSETLGFYPWKPTIPTPWRYYFQWDRTLIPSHTGTSGTPTNIYHGTDPDD 240
CN35_VP2        NTMPFTPAAMRSETLGFYPWKPTIPTPWRYYFQWDRTLIPSHTGTSGTPTNIYHGTDPDD 240
CN36_VP2        NTMPFTPAAMRSETLGFYPWKPTIPTPWRYYFQWDRTLIPSHTGTSGTPTNIYHGTDPDD 240
CN37_VP2        NTMPFTPAAMRSETLGFYPWKPTIPTPWRYYFQWDRTLIPSHTGTSGTPTNIYHGTDPDD 240
CN38_VP2        NTMPFTPAAMRSETLGFYPWKPTIPTPWRYYFQWDRTLIPSHTGTSGTPTNIYHGTDPDD 240
CN39_VP2        NTMPFTPAAMRSETLGFYPWKPTIPTPWRYYFQWDRTLIPSHTGTSGTPTNIYHGTDPDD 240
CN40_VP2        NTMPFTPAAMRSETLGFYPWKPTIPTPWRYYFQWDRTLIPSHTGTSGTPTNIYHGTDPDD 240
CK81_VP2        NTMPFTPAAMRSETLGFYPWKPTIPTPWRYYFQWDRTLIPSHTGTSGTPTNIYHGTDPDD 240
CK84_VP2        NTMPFTPAAMRSETLGFYPWKPTIPTPWRYYFQWDRTLIPSHTGTSGTPTNIYHGTDPDD 240
RVC50_VP2       NTMPFTPAAMRSETLGFYPWKPTIPTPWRYYFQWDRTLIPSHTGTSGTPTNIYHGTDPDD 240
                ************************************************************
```

```
CN7_VP2         VQFYTIENSVPVHLLRTGDEFATGTFFFDCKPCRLTHTWQTNRALGLPPFLNSLPQAEGG 300
CN8_VP2         VQFYTIENSVPVHLLRTGDEFATGTFFFDCKPCRLTHTWQTNRALGLPPFLNSLPQAEGG 300
CN10_VP2        VQFYTIENSVPVHLLRTGDEFATGTFFFDCKPCRLTHTWQTNRALGLPPFLNSLPQAEGG 300
CN11_VP2        VQFYTIENSVPVHLLRTGDEFATGTFFFDCKPCRLTHTWQTNRALGLPPFLNSLPQAEGG 300
CN12_VP2        VQFYTIENSVPVHLLRTGDEFATGTFFFDCKPCRLTHTWQTNRALGLPPFLNSLPQAEGG 300
CN13_VP2        VQFYTIENSVPVHLLRTGDEFATGTFFFDCKPCRLTHTWQTNRALGLPPFLNSLPQAEGG 300
CN14_VP2        VQFYTIENSVPVHLLRTGDEFATGTFFFDCKPCRLTHTWQTNRALGLPPFLNSLPQAEGG 300
CN15_VP2        VQFYTIENSVPVHLLRTGDEFATGTFFFDCKPCRLTHTWQTNRALGLPPFLNSLPQAEGG 300
CN16_VP2        VQFYTIENSVPVHLLRTGDEFATGTFFFDCKPCRLTHTWQTNRALGLPPFLNSLPQAEGG 300
CN17_VP2        VQFYTIENSVPVHLLRTGDEFATGTFFFDCKPCRLTHTWQTNRALGLPPFLNSLPQAEGG 300
CN18_VP2        VQFYTIENSVPVHLLRTGDEFATGTFFFDCKPCRLTHTWQTNRALGLPPFLNSLPQAEGG 300
CN19_VP2        VQFYTIENSVPVHLLRTGDEFTTGTFFFDCKPCRLTHTWQTNRALGLPPFLNSLPQAEGG 300
CN20_VP2        VQFYTIENSVPVHLLRTGDEFTTGTFFFDCKPCRLTHTWQTNRALGLPPFLNSLPQAEGG 300
CN21_VP2        VQFYTIENSVPVHLLRTGDEFATGTFFFDCKPCRLTHTWQTNRALGLPPFLNSLPQAEGG 300
CN22_VP2        VQFYTIENSVPVHLLRTGDEFATGTFFFDCKPCRLTHTWQTNRALGLPPFLNSLPQAEGG 300
CN23_VP2        VQFYTIENSVPVHLLRTGDEFATGTFFFDCKPCRLTHTWQTNRALGLPPFLNSLPQAEGG 300
CN24_VP2        VQFYTIENSVPVHLLRTGDEFTTGTFFFDCKPCRLTHTWQTNRALGLPPFLNSLPQAEGG 300
CN25_VP2        VQFYTIENSVPVHLLRTGDEFATGTFFFDCKPCRLTHTWQTNRALGLPPFLNSLPQAEGG 300
CN26_VP2        VQFYTIENSVPVHLLRTGDEFATGTFFFDCKPCRLTHTWQTNRALGLPPFLNSLPQAEGG 300
CN27_VP2        VQFYTIENSVPVHLLRTGDEFTTGTFFFDCKPCRLTHTWQTNRALGLPPFLNSLPQAEGG 300
CN28_VP2        VQFYTIENSVPVHLLRTGDEFATGTFFFDCKPCRLTHTWQTNRALGLPPFLNSLPQAEGG 300
CN29_VP2        VQFYTIENSVPVHLLRTGDEFATGTFFFDCKPCRLTHTWQTNRALGLPPFLNSLPQAEGG 300
CN30_VP2        VQFYTIENSVPVHLLRTGDEFATGTFFFDCKPCRLTHTWQTNRALGLPPFLNSLPQAEGG 300
CN31_VP2        VQFYTIENSVPVHLLRTGDEFATGTFFFDCKPCRLTHTWQTNRALGLPPFLNSLPQAEGG 300
CN32_VP2        VQFYTIENSVPVHLLRTGDEFATGTFFFDCKPCRLTHTWQTNRALGLPPFLNSLPQAEGG 300
CN33_VP2        VQFYTIENSVPVHLLRTGDEFATGTFFFDCKPCRLTHTWQTNRALGLPPFLNSLPQAEGG 300
CN34_VP2        VQFYTIENSVPVHLLRTGDEFATGTFFFDCKPCRLTHTWQTNRALGLPPFLNSLPQAEGG 300
CN35_VP2        VQFYTIENSVPVHLLRTGDEFATGTFFFDCKPCRLTHTWQTNRALGLPPFLNSLPQAEGG 300
CN36_VP2        VQFYTIENSVPVHLLRTGDEFATGTFFFDCKPCRLTHTWQTNRALGLPPFLNSLPQAEGG 300
CN37_VP2        VQFYTIENSVPVHLLRTGDEFATGTFFFDCKPCRLTHTWQTNRALGLPPFLNSLPQAEGG 300
CN38_VP2        VQFYTIENSVPVHLLRTGDEFATGTFFFDCKPCRLTHTWQTNRALGLPPFLNSLPQAEGG 300
CN39_VP2        VQFYTIENSVPVHLLRTGDEFATGTFFFDCKPCRLTHTWQTNRALGLPPFLNSLPQAEGG 300
CN40_VP2        VQFYTIENSVPVHLLRTGDEFATGTFFFDCKPCRLTHTWQTNRALGLPPFLNSLPQAEGG 300
CK81_VP2        VQFYTIENSVPVHLLRTGDEFATGTFFFDCKPCRLTHTWQTNRALGLPPFLNSLPQAEGG 300
CK84_VP2        VQFYTIENSVPVHLLRTGDEFATGTFFFDCKPCRLTHTWQTNRALGLPPFLNSLPQAEGG 300
RVC50_VP2       VQFYTIENSVPVHLLRTGDEFATGTFFFDCKPCRLTHTWQTNRALGLPPFLNSLPQAEGG 300
                *********************:**************************************

CN7_VP2         TNFGYIGVQQDKRRGVTQMGNTNYITEATIMRPAEVGYSAPYYSFEASTQGPFKTPIAAG 360
CN8_VP2         TNFGYIGVQQDKRRGVTQMGNTNYITEATIMRPAEVGYSAPYYSFEASTQGPFKTPIAAG 360
CN10_VP2        TNFGYIGVQQDKRRGVTQMGNTNYITEATIMRPAEVGYSAPYYSFEASTQGPFKTPIAAG 360
CN11_VP2        TNFGYIGVQQDKRRGVTQMGNTNYITEATIMRPAEVGYSAPYYSFEASTQGPFKTPIAAG 360
CN12_VP2        TNFGYIGVQQDKRRGVTQMGNTNYITEATIMRPAEVGYSAPYYSFEASTQGPFKTPIAAG 360
CN13_VP2        TNFGYIGVQQDKRRGVTQMGNTNYITEATIMRPAEVGYSAPYYSFEASTQGPFKTPIAAG 360
CN14_VP2        TNFGYIGVQQDKRRGVTQMGNTNYITEATIMRPAEVGYSAPYYSFEASTQGPFKTPIAAG 360
CN15_VP2        TNFGYIGVQQDKRRGVTQMGNTNYITEATIMRPAEVGYSAPYYSFEASTQGPFKTPIAAG 360
CN16_VP2        TNFGYIGVQQDKRRGVTQMGNTNYITEATIMRPAEVGYSAPYYSFEASTQGPFKTPIAAG 360
CN17_VP2        TNFGYIGVQQDKRRGVTQMGNTNYITEATIMRPAEVGYSAPYYSFEASTQGPFKTPIAAG 360
CN18_VP2        TNFGYIGVQQDKRRGVTQMGNTNYITEATIMRPAEVGYSAPYYSFEASTQGPFKTPIAAG 360
CN19_VP2        TNFGYIGVQQDKRRGVTQMGNTNYITEATIMRPAEVGYSAPYYSFEASTQGPFKTPIAAG 360
CN20_VP2        TNFGYIGVQQDKRRGVTQMGNTNYITEATIMRPAEVGYSAPYYSFEASTQGPFKTPIAAG 360
CN21_VP2        TNFGYIGVQQDKRRGVTQMGNTNYITEATIMRPAEVGYSAPYYSFEASTQGPFKTPIAAG 360
CN22_VP2        TNFGYIGVQQDKRRGVTQMGNTNYITEATIMRPAEVGYSAPYYSFEASTQGPFKTPIAAG 360
CN23_VP2        TNFGYIGVQQDKRRGVTQMGNTNYITEATIMRPAEVGYSAPYYSFEASTQGPFKTPIAAG 360
CN24_VP2        TNFGYIGVQQDKRRGVTQMGNTNYITEATIMRPAEVGYSAPYYSFEASTQGPFKTPIAAG 360
CN25_VP2        TNFGYIGVQQDKRRGVTQMGNTNYITEATIMRPAEVGYSAPYYSFEASTQGPFKTPIAAG 360
CN26_VP2        TNFGYIGVQQDKRRGVTQMGNTNYITEATIMRPAEVGYSAPYYSFEASTQGPFKTPIAAG 360
CN27_VP2        TNFGYIGVQQDKRRGVTQMGNTNYITEATIMRPAEVGYSAPYYSFEASTQGPFKTPIAAG 360
CN28_VP2        TNFGYIGVQQDKRRGVTQMGNTNYITEATIMRPAEVGYSAPYYSFEASTQGPFKTPIAAG 360
CN29_VP2        TNFGYIGVQQDKRRGVTQMGNTNYITEATIMRPAEVGYSAPYYSFEASTQGPFKTPIAAG 360
CN30_VP2        TNFGYIGVQQDKRRGVTQMGNTNYITEATIMRPAEVGYSAPYYSFEASTQGPFKTPIAAG 360
CN31_VP2        TNFGYIGVQQDKRRGVTQMGNTNYITEATIMRPAEVGYSAPYYSFEASTQGPFKTPIAAG 360
CN32_VP2        TNFGYIGVQQDKRRGVTQMGNTNYITEATIMRPAEVGYSAPYYSFEASTQGPFKTPIAAG 360
CN33_VP2        TNFGYIGVQQDKRRGVTQMGNTNYITEATIMRPAEVGYSAPYYSFEASTQGPFKTPIAAG 360
CN34_VP2        TNFGYIGVQQDKRRGVTQMGNTNYITEATIMRPAEVGYSAPYYSFEASTQGPFKTPIAAG 360
CN35_VP2        TNFGYIGVQQDKRRGVTQMGNTNYITEATIMRPAEVGYSAPYYSFEASTQGPFKTPIAAG 360
CN36_VP2        TNFGYIGVQQDKRRGVTQMGNTNYITEATIMRPAEVGYSAPYYSFEASTQGPFKTPIAAG 360
CN37_VP2        TNFGYIGVQQDKRRGVTQMGNTNYITEATIMRPAEVGYSAPYYSFEASTQGPFKTPIAAG 360
CN38_VP2        TNFGYIGVQQDKRRGVTQMGNTNYITEATIMRPAEVGYSAPYYSFEASTQGPFKTPIAAG 360
CN39_VP2        TNFGYIGVQQDKRRGVTQMGNTNYITEATIMRPAEVGYSAPYYSFEASTQGPFKTPIAAG 360
CN40_VP2        TNFGYIGVQQDKRRGVTQMGNTNYITEATIMRPAEVGYSAPYYSFEASTQGPFKTPIAAG 360
CK81_VP2        TNFGYIGVQQDKRRGVTQMGNTNYITEATIMRPAEVGYSAPYYSFEASTQGPFKTPIAAG 360
CK84_VP2        TNFGYIGVQQDKRRGVTQMGNTNYITEATIMRPAEVGYSAPYYSFEASTQGPFKTPIAAG 360
RVC50_VP2       TNFGYIGVQQDKRRGVTQMGNTNYITEATIMRPAEVGYSAPYYSFEASTQGPFKTPIAAG 360
                ************************************************************
```

```
CN7_VP2         RGGAQTDENQAADGDPRYAFGRQHGQKTTTTGETPERFTYIAHQDTGRYPEGDWIQNINF 420
CN8_VP2         RGGAQTDENQAADGDPRYAFGRQHGQKTTTTGETPERFTYIAHQDTGRYPEGDWIQNINF 420
CN10_VP2        RGGAQTDENQAADGDPRYAFGRQHGQKTTTTGETPERFTYIAHQDTGRYPEGDWIQNINF 420
CN11_VP2        RGGAQTDENQAADGDPRYAFGRQHGQKTTTTGETPERFTYIAHQDTGRYPEGDWIQNINF 420
CN12_VP2        RGGAQTDENQAADGDPRYAFGRQHGQKTTTTGETPERFTYIAHQDTGRYPEGDWIQNINF 420
CN13_VP2        RGGAQTDENQAADGDPRYAFGRQHGQKTTTTGETPERFTYIAHQDTGRYPEGDWIQNINF 420
CN14_VP2        RGGAQTDENQAANGDPRYAFGRQHGQKTTTTGETPERFTYIAHQDTGRYPEGDWIQNINF 420
CN15_VP2        RGGAQTDENQAADGDPRYAFGRQHGQKTTTTGETPERFTYIAHQDTGRYPEGDWIQNINF 420
CN16_VP2        RGGAQTDENQAADGDPRYAFGRQHGQKTTTTGETPERFTYIAHQDTGRYPEGDWIQNINF 420
CN17_VP2        RGGAQTDENQAADGDPRYAFGRQHGQKTTTTGETPERFTYIAHQDTGRYPEGDWIQNINF 420
CN18_VP2        RGGAQTDENQAADGDPRYAFGRQHGQKTTTTGETPERFTYIAHQDTGRYPEGDWIQNINF 420
CN19_VP2        RGGAQTDENQAADGDPRYAFGRQHGQKTTTTGETPERFTYIAHQDTGRYPEGDWIQNINF 420
CN20_VP2        RGGAQTDENQAADGDPRYAFGRQHGQKTTTTGETPERFTYIAHQDTGRYPEGDWIQNINF 420
CN21_VP2        RGGAQTDENQAADGDPRYAFGRQHGQKTTTTGETPERFTYIAHQDTGRYPEGDWIQNINF 420
CN22_VP2        RGGAQTDENQAADGDPRYAFGRQHGQKTTTTGETPERFTYIAHQDTGRYPEGDWIQNINF 420
CN23_VP2        RGGAQTDENQAANGDPRYAFGRQHGQKTTTTGETPERFTYIAHQDTGRYPEGDWIQNINF 420
CN24_VP2        RGGAQTDENQAADGDPRYAFGRQHGQKTTTTGETPERFTYIAHQDTGRYPEGDWIQNINF 420
CN25_VP2        RGGAQTDENQAANGDPRYAFGRQHGQKTTTTGETPERFTYIAHQDTGRYPEGDWIQNINF 420
CN26_VP2        RGGAQTDENQAADGDPRYAFGRQHGQKTTTTGETPERFTYIAHQDTGRYPEGDWIQNINF 420
CN27_VP2        RGGAQTDENQAADGDPRYAFGRQHGQKTTTTGETPERFTYIAHQDTGRYPEGDWIQNINF 420
CN28_VP2        RGGAQTDENQAADGDPRYAFGRQHGQKTTTTGETPERFTYIAHQDTGRYPEGDWIQNINF 420
CN29_VP2        RGGAQTDENQAADGDPRYAFGRQHGQKTTTTGETPERFTYIAHQDTGRYPEGDWIQNINF 420
CN30_VP2        RGGAQTDENQAADGDPRYAFGRQHGQKTTTTGETPERFTYIAHQDTGRYPEGDWIQNINF 420
CN31_VP2        RGGAQTDENQAADGDPRYAFGRQHGQKTTTTGETPERFTYIAHQDTGRYPEGDWIQNINF 420
CN32_VP2        RGGAQTDENQAADGDPRYAFGRQHGQKTTTTGETPERFTYIAHQDTGRYPEGDWIQNINF 420
CN33_VP2        RGGAQTDENQAADGDPRYAFGRQHGQKTTTTGETPERFTYIAHQDTGRYPEGDWIQNINF 420
CN34_VP2        RGGAQTDENQAADGDPRYAFGRQHGQKTTTTGETPERFTYIAHQDTGRYPEGDWIQNINF 420
CN35_VP2        RGGAQTDENQAADGDPRYAFGRQHGQKTTTTGETPERFTYIAHQDTGRYPEGDWIQNINF 420
CN36_VP2        RGGAQTDENQAADGDPRYAFGRQHGQKTTTTGETPERFTYIAHQDTGRYPEGDWIQNINF 420
CN37_VP2        RGGAQTDENQAADGDPRYAFGRQHGQKTTTTGETPERFTYIAHQDTGRYPEGDWIQNINF 420
CN38_VP2        RGGAQTDENQAADGDPRYAFGRQHGQKTTTTGETPERFTYIAHQDTGRYPEGDWIQNINF 420
CN39_VP2        RGGAQTDENQAADGDPRYAFGRQHGQKTTTTGETPERFTYIAHQDTGRYPEGDWIQNINF 420
CN40_VP2        RGGAQTDENQAADGDPRYAFGRQHGQKTTTTGETPERFTYIAHQDTGRYPEGDWIQNINF 420
CK81_VP2        RGGAQTDENQAADGDPRYAFGRQHGQKTTTTGETPERFTYIAHQDTGRYPEGDWIQNINF 420
CK84_VP2        RGGAQTDENQAADGDPRYAFGRQHGQKTTTTGETPERFTYIAHQDTGRYPEGDWIQNINF 420
RVC50_VP2       RGGAQTDENQAADGDPRYAFGRQHGQKTTTTGETPERFTYIAHQDTGRYPEGDWIQNINF 420
                ************:***********************************************

CN7_VP2         NLPVTNDNVLLPTDPIGGKTGINYTNIFNTYGPLTALNNVPPVYPNGQIWDKEFDTDLKP 480
CN8_VP2         NLPVTNDNVLLPTDPIGGKTGINYTNIFNTYGPLTALNNVPPVYPNGQIWDKEFDTDLKP 480
CN10_VP2        NLPVTNDNVLLPTDPIGGKTGINYTNIFNTYGPLTALNNVPPVYPNGQIWDKEFDTDLKP 480
CN11_VP2        NLPVTNDNVLLPTDPIGGKTGINYTNIFNTYGPLTALNNVPPVYPNGQIWDKEFDTDLKP 480
CN12_VP2        NLPVTNDNVLLPTDPIGGKTGINYTNIFNTYGPLTALNNVPPVYPNGQIWDKEFDTDLKP 480
CN13_VP2        NLPVTNDNVLLPTDPIGGKTGINYTNIFNTYGPLTALNNVPPVYPNGQIWDKEFDTDLKP 480
CN14_VP2        NLPVTNDNVLLPTDPIGGKTGINYTNIFNTYGPLTALNNVPPVYPNGQIWDKEFDTDLKP 480
CN15_VP2        NLPVTNDNVLLPTDPIGGKTGINYTNIFNTYGPLTALNNVPPVYPNGQIWDKEFDTDLKP 480
CN16_VP2        NLPVTNDNVLLPTDPIGGKTGINYTNIFNTYGPLTALNNVPPVYPNGQIWDKEFDTDLKP 480
CN17_VP2        NLPVTNDNVLLPTDPIGGKTGINYTNIFNTYGPLTALNNVPPVYPNGQIWDKEFDTDLKP 480
CN18_VP2        NLPVTNDNVLLPTDPIGGKTGINYTNIFNTYGPLTALNNVPPVYPNGQIWDKEFDTDLKP 480
CN19_VP2        NLPVTNDNVLLPTDPIGGKTGINYTNIFNTYGPLTALNNVPPVYPNGQIWDKEFDTDLKP 480
CN20_VP2        NLPVTNDNVLLPTDPIGGKTGINYTNIFNTYGPLTALNNVPPVYPNGQIWDKEFDTDLKP 480
CN21_VP2        NLPVTNDNVLLPTDPIGGKTGINYTNIFNTYGPLTALNNVPPVYPNGQIWDKEFDTDLKP 480
CN22_VP2        NLPVTNDNVLLPTDPIGGKTGINYTNIFNTYGPLTALNNVPPVYPNGQIWDKEFDTDLKP 480
CN23_VP2        NLPVTNDNVLLPTDPIGGKTGINYTNIFNTYGPLTALNNVPPVYPNGQIWDKEFDTDLKP 480
CN24_VP2        NLPVTNDNVLLPTDPIGGKTGINYTNIFNTYGPLTALNNVPPVYPNGQIWDKEFDTDLKP 480
CN25_VP2        NLPVTNDNVLLPTDPIGGKTGINYTNIFNTYGPLTALNNVPPVYPNGQIWDKEFDTDLKP 480
CN26_VP2        NLPVTNDNVLLPTDPIGGKTGINYTNIFNTYGPLTALNNVPPVYPNGQIWDKEFDTDLKP 480
CN27_VP2        NLPVTNDNVLLPTDPIGGKTGINYTNIFNTYGPLTALNNVPPVYPNGQIWDKEFDTDLKP 480
CN28_VP2        NLPVTNDNVLLPTDPIGGKTGINYTNIFNTYGPLTALNNVPPVYPNGQIWDKEFDTDLKP 480
CN29_VP2        NLPVTNDNVLLPTDPIGGKTGINYTNIFNTYGPLTALNNVPPVYPNGQIWDKEFDTDLKP 480
CN30_VP2        NLPVTNDNVLLPTDPIGGKTGINYTNIFNTYGPLTALNNVPPVYPNGQIWDKEFDTDLKP 480
CN31_VP2        NLPVTNDNVLLPTDPIGGKTGINYTNIFNTYGPLTALNNVPPVYPNGQIWDKEFDTDLKP 480
CN32_VP2        NLPVTNDNVLLPTDPIGGKTGINYTNIFNTYGPLTALNNVPPVYPNGQIWDKEFDTDLKP 480
CN33_VP2        NLPVTNDNVLLPTDPIGGKTGINYTNIFNTYGPLTALNNVPPVYPNGQIWDKEFDTDLKP 480
CN34_VP2        NLPVTNDNVLLPTDPIGGKTGINYTNIFNTYGPLTALNNVPPVYPNGQIWDKEFDTDLKP 480
CN35_VP2        NLPVTNDNVLLPTDPIGGKTGINYTNIFNTYGPLTALNNVPPVYPNGQIWDKEFDTDLKP 480
CN36_VP2        NLPVTNDNVLLPTDPIGGKTGINYTNIFNTYGPLTALNNVPPVYPNGQIWDKEFDTDLKP 480
CN37_VP2        NLPVTNDNVLLPTDPIGGKTGINYTNIFNTYGPLTALNNVPPVYPNGQIWDKEFDTDLKP 480
CN38_VP2        NLPVTNDNVLLPTDPIGGKTGINYTNIFNTYGPLTALNNVPPVYPNGQIWDKEFDTDLKP 480
CN39_VP2        NLPVTNDNVLLPTDPIGGKTGINYTNIFNTYGPLTALNNVPPVYPNGQIWDKEFDTDLKP 480
CN40_VP2        NLPVTNDNVLLPTDPIGGKTGINYTNIFNTYGPLTALNNVPPVYPNGQIWDKEFDTDLKP 480
CK81_VP2        NLPVTNDNVLLPTDPIGGKTGINYTNIFNTYGPLTALNNVPPVYPNGQIWDKEFDTDLKP 480
CK84_VP2        NLPVTNDNVLLPTDPIGGKTGINYTNIFNTYGPLTALNNVPPVYPNGQIWDKEFDTDLKP 480
RVC50_VP2       NLPVTNDNVLLPTDPIGGKTGINYTNIFNTYGPLTALNNVPPVYPNGQIWDKEFDTDLKP 480
                ************************************************************
```

```
CN7_VP2         RLHVNAPFVCQNNCPGQLFVKVAPNLTNEYDPDASANMSRIVTYSDFWWKGKLVFKAKLR 540
CN8_VP2         RLHVNAPFVCQNNCPGQLFVKVAPNLTNEYDPDASANMSRIVTYSDFWWKGKLVFKAKLR 540
CN10_VP2        RLHVNAPFVCQNNCPGQLFVKVAPNLTNEYDPDASANMSRIVTYSDFWWKGKLVFKAKLR 540
CN11_VP2        RLHVNAPFVCQNNCPGQLFVKVAPNLTNEYDPDASANMSRIVTYSDFWWKGKLVFKAKLR 540
CN12_VP2        RLHVNAPFVCQNNCPGQLFVKVAPNLTNEYDPDASANMSRIVTYSDFWWKGKLVFKAKLR 540
CN13_VP2        RLHVNAPFVCQNNCPGQLFVKVAPNLTNEYDPDASANMSRIVTYSDFWWKGKLVFKAKLR 540
CN14_VP2        RLHVNAPFVCQNNCPGQLFVKVAPNLTNEYDPDASANMSRIVTYSDFWWKGKLVFKAKLR 540
CN15_VP2        RLHVNAPFVCQNNCPGQLFVKVAPNLTNEYDPDASANMSRIVTYSDFWWKGKLVFKAKLR 540
CN16_VP2        RLHVNAPFVCQNNCPGQLFVKVAPNLTNEYDPDASANMSRIVTYSDFWWKGKLVFKAKLR 540
CN17_VP2        RLHVNAPFVCQNNCPGQLFVKVAPNLTNEYDPDASANMSRIVTYSDFWWKGKLVFKAKLR 540
CN18_VP2        RLHVNAPFVCQNNCPGQLFVKVAPNLTNEYDPDASANMSRIVTYSDFWWKGKLVFKAKLR 540
CN19_VP2        RLHVNAPFVCQNNCPGQLFVKVAPNLTNEYDPDASANMSRIVTYSDFWWKGKLVFKAKLR 540
CN20_VP2        RLHVNAPFVCQNNCPGQLFVKVAPNLTNEYDPDASANMSRIVTYSDFWWKGKLVFKAKLR 540
CN21_VP2        RLHVNAPFVCQNNCPGQLFVKVAPNLTNEYDPDASANMSRIVTYSDFWWKGKLVFKAKLR 540
CN22_VP2        RLHVNAPFVCQNNCPGQLFVKVAPNLTNEYDPDASANMSRIVTYSDFWWKGKLVFKAKLR 540
CN23_VP2        RLHVNAPFVCQNNCPGQLFVKVAPNLTNEYDPDASANMSRIVTYSDFWWKGKLVFKAKLR 540
CN24_VP2        RLHVNAPFVCQNNCPGQLFVKVAPNLTNEYDPDASANMSRIVTYSDFWWKGKLVFKAKLR 540
CN25_VP2        RLHVNAPFVCQNNCPGQLFVKVAPNLTNEYDPDASANMSRIVTYSDFWWKGKLVFKAKLR 540
CN26_VP2        RLHVNAPFVCQNNCPGQLFVKVAPNLTNEYDPDASANMSRIVTYSDFWWKGKLVFKAKLR 540
CN27_VP2        RLHVNAPFVCQNNCPGQLFVKVAPNLTNEYDPDASANMSRIVTYSDFWWKGKLVFKAKLR 540
CN28_VP2        RLHVNAPFVCQNNCPGQLFVKVAPNLTNEYDPDASANMSRIVTYSDFWWKGKLVFKAKLR 540
CN29_VP2        RLHVNAPFVCQNNCPGQLFVKVAPNLTNEYDPDASANMSRIVTYSDFWWKGKLVFKAKLR 540
CN30_VP2        RLHVNAPFVCQNNCPGQLFVKVAPNLTNEYDPDASANMSRIVTYSDFWWKGKLVFKAKLR 540
CN31_VP2        RLHVNAPFVCQNNCPGQLFVKVAPNLTNEYDPDASANMSRIVTYSDFWWKGKLVFKAKLR 540
CN32_VP2        RLHVNAPFVCQNNCPGQLFVKVAPNLTNEYDPDASANMSRIVTYSDFWWKGKLVFKAKLR 540
CN33_VP2        RLHVNAPFVCQNNCPGQLFVKVAPNLTNEYDPDASANMSRIVTYSDFWWKGKLVFKAKLR 540
CN34_VP2        RLHVNAPFVCQNNCPGQLFVKVAPNLTNEYDPDASANMSRIVTYSDFWWKGKLVFKAKLR 540
CN35_VP2        RLHVNAPFVCQNNCPGQLFVKVAPNLTNEYDPDASANMSRIVTYSDFWWKGKLVFKAKLR 540
CN36_VP2        RLHVNAPFVCQNNCPGQLFVKVAPNLTNEYDPDASANMSRIVTYSDFWWKGKLVFKAKLR 540
CN37_VP2        RLHVNAPFVCQNNCPGQLFVKVAPNLTNEYDPDASANMSRIVTYSDFWWKGKLVFKAKLR 540
CN38_VP2        RLHVNAPFVCQNNCPGQLFVKVAPNLTNEYDPDASANMSRIVTYSDFWWKGKLVFKAKLR 540
CN39_VP2        RLHVNAPFVCQNNCPGQLFVKVAPNLTNEYDPDASANMSRIVTYSDFWWKGKLVFKAKLR 540
CN40_VP2        RLHVNAPFVCQNNCPGQLFVKVAPNLTNEYDPDASANMSRIVTYSDFWWKGKLVFKAKLR 540
CK81_VP2        RLHVNAPFVCQNNCPGQLFVKVAPNLTNEYDPDASANMSRIVTYSDFWWKGKLVFKAKLR 540
CK84_VP2        RLHVNAPFVCQNNCPGQLFVKVAPNLTNEYDPDASANMSRIVTYSDFWWKGKLVFKAKLR 540
RVC50_VP2       RLHVNAPFVCQNNCPGQLFVKVAPNLTNEYDPDASANMSRIVTYSDFWWKGKLVFKAKLR 540
                ************************************************************

CN7_VP2         ASHTWNPIQQMSINVDNQFNYVPSNIGGMKIVYEKSQLAPRKLY 584
CN8_VP2         ASHTWNPIQQMSINVDNQFNYVPSNIGGMKIVYEKSQLAPRKLY 584
CN10_VP2        ASHTWNPIQQMSINVDNQFNYVPSNIGGMKIVYEKSQLAPRKLY 584
CN11_VP2        ASHTWNPIQQMSINVDNQFNYVPSNIGGMKIVYEKSQLAPRKLY 584
CN12_VP2        ASHTWNPIQQMSINVDNQFNYVPSNIGGMKIVYEKSQLAPRKLY 584
CN13_VP2        ASHTWNPIQQMSINVDNQFNYVPSNIGGMKIVYEKSQLAPRKLY 584
CN14_VP2        ASHTWNPIQQMSINVDNQFNYVPSNIGGMKIVYEKSQLAPRKLY 584
CN15_VP2        ASHTWNPIQQMSINVDNQFNYVPSNIGGMKIVYEKSQLAPRKLY 584
CN16_VP2        ASHTWNPIQQMSINVDNQFNYVPSNIGGMKIVYEKSQLAPRKLY 584
CN17_VP2        ASHTWNPIQQMSINVDNQFNYVPSNIGGMKIVYEKSQLAPRKLY 584
CN18_VP2        ASHTWNPIQQMSINVDNQFNYVPSNIGGMKIVYEKSQLAPRKLY 584
CN19_VP2        ASHTWNPIQQMSINVDNQFNYVPSNIGGMKIVYEKSQLAPRKLY 584
CN20_VP2        ASHTWNPIQQMSINVDNQFNYVPSNIGGMKIVYEKSQLAPRKLY 584
CN21_VP2        ASHTWNPIQQMSINVDNQFNYVPSNIGGMKIVYEKSQLAPRKLY 584
CN22_VP2        ASHTWNPIQQMSINVDNQFNYVPSNIGGMKIVYEKSQLAPRKLY 584
CN23_VP2        ASHTWNPIQQMSINVDNQFNYVPSNIGGMKIVYEKSQLAPRKLY 584
CN24_VP2        ASHTWNPIQQMSINVDNQFNYVPSNIGGMKIVYEKSQLAPRKLY 584
CN25_VP2        ASHTWNPIQQMSINVDNQFNYVPSNIGGMKIVYEKSQLAPRKLY 584
CN26_VP2        ASHTWNPIQQMSINVDNQFNYVPSNIGGMKIVYEKSQLAPRKLY 584
CN27_VP2        ASHTWNPIQQMSINVDNQFNYVPSNIGGMKIVYEKSQLAPRKLY 584
CN28_VP2        ASHTWNPIQQMSINVDNQFNYVPSNIGGMKIVYEKSQLAPRKLY 584
CN29_VP2        ASHTWNPIQQMSINVDNQFNYVPSNIGGMKIVYEKSQLAPRKLY 584
CN30_VP2        ASHTWNPIQQMSINVDNQFNYVPSNIGGMKIVYEKSQLAPRKLY 584
CN31_VP2        ASHTWNPIQQMSINVDNQFNYVPSNIGGMKIVYEKSQLAPRKLY 584
CN32_VP2        ASHTWNPIQQMSINVDNQFNYVPSNIGGMKIVYEKSQLAPRKLY 584
CN33_VP2        ASHTWNPIQQMSINVDNQFNYVPSNIGGMKIVYEKSQLAPRKLY 584
CN34_VP2        ASHTWNPIQQMSINVDNQFNYVPSNIGGMKIVYEKSQLAPRKLY 584
CN35_VP2        ASHTWNPIQQMSINVDNQFNYVPSNIGGMKIVYEKSQLAPRKLY 584
CN36_VP2        ASHTWNPIQQMSINVDNQFNYVPSNIGGMKIVYEKSQLAPRKLY 584
CN37_VP2        ASHTWNPIQQMSINVDNQFNYVPSNIGGMKIVYEKSQLAPRKLY 584
CN38_VP2        ASHTWNPIQQMSINVDNQFNYVPSNIGGMKIVYEKSQLAPRKLY 584
CN39_VP2        ASHTWNPIQQMSINVDNQFNYVPSNIGGMKIVYEKSQLAPRKLY 584
CN40_VP2        ASHTWNPIQQMSINVDNQFNYVPSNIGGMKIVYEKSQLAPRKLY 584
CK81_VP2        ASHTWNPIQQMSINVDNQFNYVPSNIGGMKIVYEKSQLAPRKLY 584
CK84_VP2        ASHTWNPIQQMSINVDNQFNYVPSNIGGMKIVYEKSQLAPRKLY 584
RVC50_VP2       ASHTWNPIQQMSINVDNQFNYVPSNIGGMKIVYEKSQLAPRKLY 584
                ********************************************
```
