# Supplementary material for: Molecular Investigation of Canine Parvovirus-2 (CPV-2) Outbreak in Nevis Island: Analysis of the Nearly Complete Genomes of CPV-2 Strains from the Caribbean Region
Source: Viruses. 2021 Jun 6;13(6):1083. doi: 10.3390/v13061083 (PMC8227521; doi:10.3390/v13061083)
Supplement: Supplementary file 1 [file viruses-13-01083-s001.zip › Supplementary materials_R1/Supplementary material S3.pdf]

Supplementary material S3. Multiple alignment of the VP2 coding sequences of canine parvovirus-2 (CPV-2) strains detected in Nevis (designated with prefix CN) during 2020 with those reported from the neighboring island of St. Kitts in 2016 (strain RVC50) and in 2020 (strains CK81 and CK84). Synonymous mutations are indicated with green, whilst nonsynonymous mutations are shown with red. A '*' denotes an identical nucleotide (nt) residue, whilst '-' indicates absence of a nt residue. Numbers to the right indicate the positions of the nt for respective CPV-2 strains.

```
CN7_VP2         ATGAGTGATGGAGCAGTTCAACCAGACGGTGGTCAACCTGCTGTCAGAAATGAAAGAGCA 60
CN8_VP2         ATGAGTGATGGAGCAGTTCAACCAGACGGTGGTCAACCTGCTGTCAGAAATGAAAGAGCA 60
CN10_VP2        ATGAGTGATGGAGCAGTTCAACCAGACGGTGGTCAACCTGCTGTCAGAAATGAAAGAGCA 60
CN11_VP2        ATGAGTGATGGAGCAGTTCAACCAGACGGTGGTCAACCTGCTGTCAGAAATGAAAGAGCA 60
CN12_VP2        ATGAGTGATGGAGCAGTTCAACCAGACGGTGGTCAACCTGCTGTCAGAAATGAAAGAGCA 60
CN13_VP2        ATGAGTGATGGAGCAGTTCAACCAGACGGTGGTCAACCTGCTGTCAGAAATGAAAGAGCA 60
CN14_VP2        ATGAGTGATGGAGCAGTTCAACCAGACGGTGGTCAACCTGCTGTCAGAAATGAAAGAGCA 60
CN15_VP2        ATGAGTGATGGAGCAGTTCAACCAGACGGTGGTCAACCTGCTGTCAGAAATGAAAGAGCA 60
CN16_VP2        ATGAGTGATGGAGCAGTTCAACCAGACGGTGGTCAACCTGCTGTCAGAAATGAAAGAGCA 60
CN17_VP2        ATGAGTGATGGAGCAGTTCAACCAGACGGTGGTCAACCTGCTGTCAGAAATGAAAGAGCA 60
CN18_VP2        ATGAGTGATGGAGCAGTTCAACCAGACGGTGGTCAACCTGCTGTCAGAAATGAAAGAGCA 60
CN19_VP2        ATGAGTGATGGAGCAGTTCAACCAGACGGTGGTCAACCTGCTGTCAGAAATGAAAGAGCA 60
CN20_VP2        ATGAGTGATGGAGCAGTTCAACCAGACGGTGGTCAACCTGCTGTCAGAAATGAAAGAGCA 60
CN21_VP2        ATGAGTGATGGAGCAGTTCAACCAGACGGTGGTCAACCTGCTGTCAGAAATGAAAGAGCA 60
CN22_VP2        ATGAGTGATGGAGCAGTTCAACCAGACGGTGGTCAACCTGCTGTCAGAAATGAAAGAGCA 60
CN23_VP2        ATGAGTGATGGAGCAGTTCAACCAGACGGTGGTCAACCTGCTGTCAGAAATGAAAGAGCA 60
CN24_VP2        ATGAGTGATGGAGCAGTTCAACCAGACGGTGGTCAACCTGCTGTCAGAAATGAAAGAGCA 60
CN25_VP2        ATGAGTGATGGAGCAGTTCAACCAGACGGTGGTCAACCTGCTGTCAGAAATGAAAGAGCA 60
CN26_VP2        ATGAGTGATGGAGCAGTTCAACCAGACGGTGGTCAACCTGCTGTCAGAAATGAAAGAGCA 60
CN27_VP2        ATGAGTGATGGAGCAGTTCAACCAGACGGTGGTCAACCTGCTGTCAGAAATGAAAGAGCA 60
CN28_VP2        ATGAGTGATGGAGCAGTTCAACCAGACGGTGGTCAACCTGCTGTCAGAAATGAAAGAGCA 60
CN29_VP2        ATGAGTGATGGAGCAGTTCAACCAGACGGTGGTCAACCTGCTGTCAGAAATGAAAGAGCA 60
CN30_VP2        ATGAGTGATGGAGCAGTTCAACCAGACGGTGGTCAACCTGCTGTCAGAAATGAAAGAGCA 60
CN31_VP2        ATGAGTGATGGAGCAGTTCAACCAGACGGTGGTCAACCTGCTGTCAGAAATGAAAGAGCA 60
CN32_VP2        ATGAGTGATGGAGCAGTTCAACCAGACGGTGGTCAACCTGCTGTCAGAAATGAAAGAGCA 60
CN33_VP2        ATGAGTGATGGAGCAGTTCAACCAGACGGTGGTCAACCTGCTGTCAGAAATGAAAGAGCA 60
CN34_VP2        ATGAGTGATGGAGCAGTTCAACCAGACGGTGGTCAACCTGCTGTCAGAAATGAAAGAGCA 60
CN35_VP2        ATGAGTGATGGAGCAGTTCAACCAGACGGTGGTCAACCTGCTGTCAGAAATGAAAGAGCA 60
CN36_VP2        ATGAGTGATGGAGCAGTTCAACCAGACGGTGGTCAACCTGCTGTCAGAAATGAAAGAGCA 60
CN37_VP2        ATGAGTGATGGAGCAGTTCAACCAGACGGTGGTCAACCTGCTGTCAGAAATGAAAGAGCA 60
CN38_VP2        ATGAGTGATGGAGCAGTTCAACCAGACGGTGGTCAACCTGCTGTCAGAAATGAAAGAGCA 60
CN39_VP2        ATGAGTGATGGAGCAGTTCAACCAGACGGTGGTCAACCTGCTGTCAGAAATGAAAGAGCA 60
CN40_VP2        ATGAGTGATGGAGCAGTTCAACCAGACGGTGGTCAACCTGCTGTCAGAAATGAAAGAGCA 60
CK81_VP2        ATGAGTGATGGAGCAGTTCAACCAGACGGTGGTCAACCTGCTGTCAGAAATGAAAGAGCA 60
CK84_VP2        ATGAGTGATGGAGCAGTTCAACCAGACGGTGGTCAACCTGCTGTCAGAAATGAAAGAGCA 60
RVC50_VP2       ATGAGTGATGGAGCAGTTCAACCAGACGGTGGTCAACCTGCTGTCAGAAATGAAAGAGCA 60
                ************************************************************

CN7_VP2         ACAGGATCTGGGAACGGGTCTGGAGGCGGGGGTGGTGGTGGTTCTGGGGGTGTGGGGATT 120
CN8_VP2         ACAGGATCTGGGAACGGGTCTGGAGGCGGGGGTGGTGGTGGTTCTGGGGGTGTGGGGATT 120
CN10_VP2        ACAGGATCTGGGAACGGGTCTGGAGGCGGGGGTGGTGGTGGTTCTGGGGGTGTGGGGATT 120
CN11_VP2        ACAGGATCTGGGAACGGGTCTGGAGGCGGGGGTGGTGGTGGTTCTGGGGGTGTGGGGATT 120
CN12_VP2        ACAGGATCTGGGAACGGGTCTGGAGGCGGGGGTGGTGGTGGTTCTGGGGGTGTGGGGATT 120
CN13_VP2        ACAGGATCTGGGAACGGGTCTGGAGGCGGGGGTGGTGGTGGTTCTGGGGGTGTGGGGATT 120
CN14_VP2        ACAGGATCTGGGAACGGGTCTGGAGGCGGGGGTGGTGGTGGTTCTGGGGGTGTGGGGATT 120
CN15_VP2        ACAGGATCTGGGAACGGGTCTGGAGGCGGGGGTGGTGGTGGTTCTGGGGGTGTGGGGATT 120
CN16_VP2        ACAGGATCTGGGAACGGGTCTGGAGGCGGGGGTGGTGGTGGTTCTGGGGGTGTGGGGATT 120
CN17_VP2        ACAGGATCTGGGAACGGGTCTGGAGGCGGGGGTGGTGGTGGTTCTGGGGGTGTGGGGATT 120
CN18_VP2        ACAGGATCTGGGAACGGGTCTGGAGGCGGGGGTGGTGGTGGTTCTGGGGGTGTGGGGATT 120
CN19_VP2        ACAGGATCTGGGAACGGGTCTGGAGGCGGGGGTGGTGGTGGTTCTGGGGGTGTGGGGATT 120
CN20_VP2        ACAGGATCTGGGAACGGGTCTGGAGGCGGGGGTGGTGGTGGTTCTGGGGGTGTGGGGATT 120
CN21_VP2        ACAGGATCTGGGAACGGGTCTGGAGGCGGGGGTGGTGGTGGTTCTGGGGGTGTGGGGATT 120
CN22_VP2        ACAGGATCTGGGAACGGGTCTGGAGGCGGGGGTGGTGGTGGTTCTGGGGGTGTGGGGATT 120
CN23_VP2        ACAGGATCTGGGAACGGGTCTGGAGGCGGGGGTGGTGGTGGTTCTGGGGGTGTGGGGATT 120
CN24_VP2        ACAGGATCTGGGAACGGGTCTGGAGGCGGGGGTGGTGGTGGTTCTGGGGGTGTGGGGATT 120
CN25_VP2        ACAGGATCTGGGAACGGGTCTGGAGGCGGGGGTGGTGGTGGTTCTGGGGGTGTGGGGATT 120
CN26_VP2        ACAGGATCTGGGAACGGGTCTGGAGGCGGGGGTGGTGGTGGTTCTGGGGGTGTGGGGATT 120
CN27_VP2        ACAGGATCTGGGAACGGGTCTGGAGGCGGGGGTGGTGGTGGTTCTGGGGGTGTGGGGATT 120
CN28_VP2        ACAGGATCTGGGAACGGGTCTGGAGGCGGGGGTGGTGGTGGTTCTGGGGGTGTGGGGATT 120
CN29_VP2        ACAGGATCTGGGAACGGGTCTGGAGGCGGGGGTGGTGGTGGTTCTGGGGGTGTGGGGATT 120
CN30_VP2        ACAGGATCTGGGAACGGGTCTGGAGGCGGGGGTGGTGGTGGTTCTGGGGGTGTGGGGATT 120
CN31_VP2        ACAGGATCTGGGAACGGGTCTGGAGGCGGGGGTGGTGGTGGTTCTGGGGGTGTGGGGATT 120
CN32_VP2        ACAGGATCTGGGAACGGGTCTGGAGGCGGGGGTGGTGGTGGTTCTGGGGGTGTGGGGATT 120
CN33_VP2        ACAGGATCTGGGAACGGGTCTGGAGGCGGGGGTGGTGGTGGTTCTGGGGGTGTGGGGATT 120
CN34_VP2        ACAGGATCTGGGAACGGGTCTGGAGGCGGGGGTGGTGGTGGTTCTGGGGGTGTGGGGATT 120
CN35_VP2        ACAGGATCTGGGAACGGGTCTGGAGGCGGGGGTGGTGGTGGTTCTGGGGGTGTGGGGATT 120
CN36_VP2        ACAGGATCTGGGAACGGGTCTGGAGGCGGGGGTGGTGGTGGTTCTGGGGGTGTGGGGATT 120
CN37_VP2        ACAGGATCTGGGAACGGGTCTGGAGGCGGGGGTGGTGGTGGTTCTGGGGGTGTGGGGATT 120
CN38_VP2        ACAGGATCTGGGAACGGGTCTGGAGGCGGGGGTGGTGGTGGTTCTGGGGGTGTGGGGATT 120
CN39_VP2        ACAGGATCTGGGAACGGGTCTGGAGGCGGGGGTGGTGGTGGTTCTGGGGGTGTGGGGATT 120
CN40_VP2        ACAGGATCTGGGAACGGGTCTGGAGGCGGGGGTGGTGGTGGTTCTGGGGGTGTGGGGATT 120
CK81_VP2        ACAGGATCTGGGAACGGGTCTGGAGGCGGGGGTGGTGGTGGTTCTGGGGGTGTGGGGATT 120
CK84_VP2        ACAGGATCTGGGAACGGGTCTGGAGGCGGGGGTGGTGGTGGTTCTGGGGGTGTGGGGATT 120
RVC50_VP2       ACAGGATCTGGGAACGGGTCTGGAGGCGGGGGTGGTGGTGGTTCTGGGGGTGTGGGGATT 120
                ************************************************************
```

```
CN7_VP2         TCTACGGGTACTTTCAATAATCAGACGGAATTTAAATTTTTGGAAAACGGATGGGTGGAA 180
CN8_VP2         TCTACGGGTACTTTCAATAATCAGACGGAATTTAAATTTTTGGAAAACGGATGGGTGGAA 180
CN10_VP2        TCTACGGGTACTTTCAATAATCAGACGGAATTTAAATTTTTGGAAAACGGATGGGTGGAA 180
CN11_VP2        TCTACGGGTACTTTCAATAATCAGACGGAATTTAAATTTTTGGAAAACGGATGGGTGGAA 180
CN12_VP2        TCTACGGGTACTTTCAATAATCAGACGGAATTTAAATTTTTGGAAAACGGATGGGTGGAA 180
CN13_VP2        TCTACGGGTACTTTCAATAATCAGACGGAATTTAAATTTTTGGAAAACGGATGGGTGGAA 180
CN14_VP2        TCTACGGGTACTTTCAATAATCAGACGGAATTTAAATTTTTGGAAAACGGATGGGTGGAA 180
CN15_VP2        TCTACGGGTACTTTCAATAATCAGACGGAATTTAAATTTTTGGAAAACGGATGGGTGGAA 180
CN16_VP2        TCTACGGGTACTTTCAATAATCAGACGGAATTTAAATTTTTGGAAAACGGATGGGTGGAA 180
CN17_VP2        TCTACGGGTACTTTCAATAATCAGACGGAATTTAAATTTTTGGAAAACGGATGGGTGGAA 180
CN18_VP2        TCTACGGGTACTTTCAATAATCAGACGGAATTTAAATTTTTGGAAAACGGATGGGTGGAA 180
CN19_VP2        TCTACGGGTACTTTCAATAATCAGACGGAATTTAAATTTTTGGAAAACGGATGGGTGGAA 180
CN20_VP2        TCTACGGGTACTTTCAATAATCAGACGGAATTTAAATTTTTGGAAAACGGATGGGTGGAA 180
CN21_VP2        TCTACGGGTACTTTCAATAATCAGACGGAATTTAAATTTTTGGAAAACGGATGGGTGGAA 180
CN22_VP2        TCTACGGGTACTTTCAATAATCAGACGGAATTTAAATTTTTGGAAAACGGATGGGTGGAA 180
CN23_VP2        TCTACGGGTACTTTCAATAATCAGACGGAATTTAAATTTTTGGAAAACGGATGGGTGGAA 180
CN24_VP2        TCTACGGGTACTTTCAATAATCAGACGGAATTTAAATTTTTGGAAAACGGATGGGTGGAA 180
CN25_VP2        TCTACGGGTACTTTCAATAATCAGACGGAATTTAAATTTTTGGAAAACGGATGGGTGGAA 180
CN26_VP2        TCTACGGGTACTTTCAATAATCAGACGGAATTTAAATTTTTGGAAAACGGATGGGTGGAA 180
CN27_VP2        TCTACGGGTACTTTCAATAATCAGACGGAATTTAAATTTTTGGAAAACGGATGGGTGGAA 180
CN28_VP2        TCTACGGGTACTTTCAATAATCAGACGGAATTTAAATTTTTGGAAAACGGATGGGTGGAA 180
CN29_VP2        TCTACGGGTACTTTCAATAATCAGACGGAATTTAAATTTTTGGAAAACGGATGGGTGGAA 180
CN30_VP2        TCTACGGGTACTTTCAATAATCAGACGGAATTTAAATTTTTGGAAAACGGATGGGTGGAA 180
CN31_VP2        TCTACGGGTACTTTCAATAATCAGACGGAATTTAAATTTTTGGAAAACGGATGGGTGGAA 180
CN32_VP2        TCTACGGGTACTTTCAATAATCAGACGGAATTTAAATTTTTGGAAAACGGATGGGTGGAA 180
CN33_VP2        TCTACGGGTACTTTCAATAATCAGACGGAATTTAAATTTTTGGAAAACGGATGGGTGGAA 180
CN34_VP2        TCTACGGGTACTTTCAATAATCAGACGGAATTTAAATTTTTGGAAAACGGATGGGTGGAA 180
CN35_VP2        TCTACGGGTACTTTCAATAATCAGACGGAATTTAAATTTTTGGAAAACGGATGGGTGGAA 180
CN36_VP2        TCTACGGGTACTTTCAATAATCAGACGGAATTTAAATTTTTGGAAAACGGATGGGTGGAA 180
CN37_VP2        TCTACGGGTACTTTCAATAATCAGACGGAATTTAAATTTTTGGAAAACGGATGGGTGGAA 180
CN38_VP2        TCTACGGGTACTTTCAATAATCAGACGGAATTTAAATTTTTGGAAAACGGATGGGTGGAA 180
CN39_VP2        TCTACGGGTACTTTCAATAATCAGACGGAATTTAAATTTTTGGAAAACGGATGGGTGGAA 180
CN40_VP2        TCTACGGGTACTTTCAATAATCAGACGGAATTTAAATTTTTGGAAAACGGATGGGTGGAA 180
CK81_VP2        TCTACGGGTACTTTCAATAATCAGACGGAATTTAAATTTTTGGAAAACGGATGGGTGGAA 180
CK84_VP2        TCTACGGGTACTTTCAATAATCAGACGGAATTTAAATTTTTGGAAAACGGATGGGTGGAA 180
RVC50_VP2       TCTACGGGTACTTTCAATAATCAGACGGAATTTAAATTTTTGGAAAACGGATGGGTGGAA 180
                ************************************************************

CN7_VP2         ATCACAGCAAACTCAAGCAGACTTGTACATTTAAATATGCCAGAAAGTGAAAATTATAGA 240
CN8_VP2         ATCACAGCAAACTCAAGCAGACTTGTACATTTAAATATGCCAGAAAGTGAAAATTATAGA 240
CN10_VP2        ATCACAGCAAACTCAAGCAGACTTGTACATTTAAATATGCCAGAAAGTGAAAATTATAGA 240
CN11_VP2        ATCACAGCAAACTCAAGCAGACTTGTACATTTAAATATGCCAGAAAGTGAAAATTATAGA 240
CN12_VP2        ATCACAGCAAACTCAAGCAGACTTGTACATTTAAATATGCCAGAAAGTGAAAATTATAGA 240
CN13_VP2        ATCACAGCAAACTCAAGCAGACTTGTACATTTAAATATGCCAGAAAGTGAAAATTATAGA 240
CN14_VP2        ATCACAGCAAACTCAAGCAGACTTGTACATTTAAATATGCCAGAAAGTGAAAATTATAGA 240
CN15_VP2        ATCACAGCAAACTCAAGCAGACTTGTACATTTAAATATGCCAGAAAGTGAAAATTATAGA 240
CN16_VP2        ATCACAGCAAACTCAAGCAGACTTGTACATTTAAATATGCCAGAAAGTGAAAATTATAGA 240
CN17_VP2        ATCACAGCAAACTCAAGCAGACTTGTACATTTAAATATGCCAGAAAGTGAAAATTATAGA 240
CN18_VP2        ATCACAGCAAACTCAAGCAGACTTGTACATTTAAATATGCCAGAAAGTGAAAATTATAGA 240
CN19_VP2        ATCACAGCAAACTCAAGCAGACTTGTACATTTAAATATGCCAGAAAGTGAAAATTATAGA 240
CN20_VP2        ATCACAGCAAACTCAAGCAGACTTGTACATTTAAATATGCCAGAAAGTGAAAATTATAGA 240
CN21_VP2        ATCACAGCAAACTCAAGCAGACTTGTACATTTAAATATGCCAGAAAGTGAAAATTATAGA 240
CN22_VP2        ATCACAGCAAACTCAAGCAGACTTGTACATTTAAATATGCCAGAAAGTGAAAATTATAGA 240
CN23_VP2        ATCACAGCAAACTCAAGCAGACTTGTACATTTAAATATGCCAGAAAGTGAAAATTATAGA 240
CN24_VP2        ATCACAGCAAACTCAAGCAGACTTGTACATTTAAATATGCCAGAAAGTGAAAATTATAGA 240
CN25_VP2        ATCACAGCAAACTCAAGCAGACTTGTACATTTAAATATGCCAGAAAGTGAAAATTATAGA 240
CN26_VP2        ATCACAGCAAACTCAAGCAGACTTGTACATTTAAATATGCCAGAAAGTGAAAATTATAGA 240
CN27_VP2        ATCACAGCAAACTCAAGCAGACTTGTACATTTAAATATGCCAGAAAGTGAAAATTATAGA 240
CN28_VP2        ATCACAGCAAACTCAAGCAGACTTGTACATTTAAATATGCCAGAAAGTGAAAATTATAGA 240
CN29_VP2        ATCACAGCAAACTCAAGCAGACTTGTACATTTAAATATGCCAGAAAGTGAAAATTATAGA 240
CN30_VP2        ATCACAGCAAACTCAAGCAGACTTGTACATTTAAATATGCCAGAAAGTGAAAATTATAGA 240
CN31_VP2        ATCACAGCAAACTCAAGCAGACTTGTACATTTAAATATGCCAGAAAGTGAAAATTATAGA 240
CN32_VP2        ATCACAGCAAACTCAAGCAGACTTGTACATTTAAATATGCCAGAAAGTGAAAATTATAGA 240
CN33_VP2        ATCACAGCAAACTCAAGCAGACTTGTACATTTAAATATGCCAGAAAGTGAAAATTATAGA 240
CN34_VP2        ATCACAGCAAACTCAAGCAGACTTGTACATTTAAATATGCCAGAAAGTGAAAATTATAGA 240
CN35_VP2        ATCACAGCAAACTCAAGCAGACTTGTACATTTAAATATGCCAGAAAGTGAAAATTATAGA 240
CN36_VP2        ATCACAGCAAACTCAAGCAGACTTGTACATTTAAATATGCCAGAAAGTGAAAATTATAGA 240
CN37_VP2        ATCACAGCAAACTCAAGCAGACTTGTACATTTAAATATGCCAGAAAGTGAAAATTATAGA 240
CN38_VP2        ATCACAGCAAACTCAAGCAGACTTGTACATTTAAATATGCCAGAAAGTGAAAATTATAGA 240
CN39_VP2        ATCACAGCAAACTCAAGCAGACTTGTACATTTAAATATGCCAGAAAGTGAAAATTATAGA 240
CN40_VP2        ATCACAGCAAACTCAAGCAGACTTGTACATTTAAATATGCCAGAAAGTGAAAATTATAGA 240
CK81_VP2        ATCACAGCAAACTCAAGCAGACTTGTACATTTAAATATGCCAGAAAGTGAAAATTATAGA 240
CK84_VP2        ATCACAGCAAACTCAAGCAGACTTGTACATTTAAATATGCCAGAAAGTGAAAATTATAGA 240
RVC50_VP2       ATCACAGCAAACTCAAGCAGACTTGTACATTTAAATATGCCAGAAAGTGAAAATTATAGA 240
                ************************************************************
```

```
CN7_VP2         AGAGTGGTTGTAAATAATTTGGATAAAACTGCAGTTAACGGAAACATGGCTTTAGATGAT 300
CN8_VP2         AGAGTGGTTGTAAATAATTTAGATAAAACTGCAGTTAACGGAAACATGGCTTTAGATGAT 300
CN10_VP2        AGAGTGGTTGTAAATAATTTGGATAAAACTGCAGTTAACGGAAACATGGCTTTAGATGAT 300
CN11_VP2        AGAGTGGTTGTAAATAATTTGGATAAAACTGCAGTTAACGGAAACATGGCTTTAGATGAT 300
CN12_VP2        AGAGTGGTTGTAAATAATTTAGATAAAACTGCAGTTAACGGAAACATGGCTTTAGATGAT 300
CN13_VP2        AGAGTGGTTGTAAATAATTTAGATAAAACTGCAGTTAACGGAAACATGGCTTTAGATGAT 300
CN14_VP2        AGAGTGGTTGTAAATAATTTGGATAAAACTGCAGTTAACGGAAACATGGCTTTAGATGAT 300
CN15_VP2        AGAGTGGTTGTAAATAATTTGGATAAAACTGCAGTTAACGGAAACATGGCTTTAGATGAT 300
CN16_VP2        AGAGTGGTTGTAAATAATTTAGATAAAACTGCAGTTAACGGAAACATGGCTTTAGATGAT 300
CN17_VP2        AGAGTGGTTGTAAATAATTTGGATAAAACTGCAGTTAACGGAAACATGGCTTTAGATGAT 300
CN18_VP2        AGAGTGGTTGTAAATAATTTAGATAAAACTGCAGTTAACGGAAACATGGCTTTAGATGAT 300
CN19_VP2        AGAGTGGTTGTAAATAATTTGGATAAAACTGCAGTTAACGGAAACATGGCTTTAGATGAT 300
CN20_VP2        AGAGTGGTTGTAAATAATTTGGATAAAACTGCAGTTAACGGAAACATGGCTTTAGATGAT 300
CN21_VP2        AGAGTGGTTGTAAATAATTTGGATAAAACTGCAGTTAACGGAAACATGGCTTTAGATGAT 300
CN22_VP2        AGAGTGGTTGTAAATAATTTGGATAAAACTGCAGTTAACGGAAACATGGCTTTAGATGAT 300
CN23_VP2        AGAGTGGTTGTAAATAATTTGGATAAAACTGCAGTTAACGGAAACATGGCTTTAGATGAT 300
CN24_VP2        AGAGTGGTTGTAAATAATTTGGATAAAACTGCAGTTAACGGAAACATGGCTTTAGATGAT 300
CN25_VP2        AGAGTGGTTGTAAATAATTTGGATAAAACTGCAGTTAACGGAAACATGGCTTTAGATGAT 300
CN26_VP2        AGAGTGGTTGTAAATAATTTGGATAAAACTGCAGTTAACGGAAACATGGCTTTAGATGAT 300
CN27_VP2        AGAGTGGTTGTAAATAATTTGGATAAAACTGCAGTTAACGGAAACATGGCTTTAGATGAT 300
CN28_VP2        AGAGTGGTTGTAAATAATTTGGATAAAACTGCAGTTAACGGAAACATGGCTTTAGATGAT 300
CN29_VP2        AGAGTGGTTGTAAATAATTTAGATAAAACTGCAGTTAACGGAAACATGGCTTTAGATGAT 300
CN30_VP2        AGAGTGGTTGTAAATAATTTGGATAAAACTGCAGTTAACGGAAACATGGCTTTAGATGAT 300
CN31_VP2        AGAGTGGTTGTAAATAATTTGGATAAAACTGCAGTTAACGGAAACATGGCTTTAGATGAT 300
CN32_VP2        AGAGTGGTTGTAAATAATTTGGATAAAACTGCAGTTAACGGAAACATGGCTTTAGATGAT 300
CN33_VP2        AGAGTGGTTGTAAATAATTTGGATAAAACTGCAGTTAACGGAAACATGGCTTTAGATGAT 300
CN34_VP2        AGAGTGGTTGTAAATAATTTAGATAAAACTGCAGTTAACGGAAACATGGCTTTAGATGAT 300
CN35_VP2        AGAGTGGTTGTAAATAATTTGGATAAAACTGCAGTTAACGGAAACATGGCTTTAGATGAT 300
CN36_VP2        AGAGTGGTTGTAAATAATTTGGATAAAACTGCAGTTAACGGAAACATGGCTTTAGATGAT 300
CN37_VP2        AGAGTGGTTGTAAATAATTTGGATAAAACTGCAGTTAACGGAAACATGGCTTTAGATGAT 300
CN38_VP2        AGAGTGGTTGTAAATAATTTGGATAAAACTGCAGTTAACGGAAACATGGCTTTAGATGAT 300
CN39_VP2        AGAGTGGTTGTAAATAATTTAGATAAAACTGCAGTTAACGGAAACATGGCTTTAGATGAT 300
CN40_VP2        AGAGTGGTTGTAAATAATTTGGATAAAACTGCAGTTAACGGAAACATGGCTTTAGATGAT 300
CK81_VP2        AGAGTGGTTGTAAATAATTTGGATAAAACTGCAGTTAACGGAAACATGGCTTTAGATGAT 300
CK84_VP2        AGAGTGGTTGTAAATAATTTGGATAAAACTGCAGTTAACGGAAACATGGCTTTAGATGAT 300
RVC50_VP2       AGAGTGGTTGTAAATAATTTGGATAAAACTGCAGTTAACGGAAACATGGCTTTAGATGAT 300
                ******************** ***************************************

CN7_VP2         ACTCATGCACAAATTGTAACACCTTGGTCATTGGTTGATGCAAATGCTTGGGGAGTTTGG 360
CN8_VP2         ACTCATGCACAAATTGTAACACCTTGGTCATTGGTTGATGCAAATGCTTGGGGAGTTTGG 360
CN10_VP2        ACTCATGCACAAATTGTAACACCTTGGTCATTGGTTGATGCAAATGCTTGGGGAGTTTGG 360
CN11_VP2        ACTCATGCACAAATTGTAACACCTTGGTCATTGGTTGATGCAAATGCTTGGGGAGTTTGG 360
CN12_VP2        ACTCATGCACAAATTGTAACACCTTGGTCATTGGTTGATGCAAATGCTTGGGGAGTTTGG 360
CN13_VP2        ACTCATGCACAAATTGTAACACCTTGGTCATTGGTTGATGCAAATGCTTGGGGAGTTTGG 360
CN14_VP2        ACTCATGCACAAATTGTAACACCTTGGTCATTGGTTGATGCAAATGCTTGGGGAGTTTGG 360
CN15_VP2        ACTCATGCACAAATTGTAACACCTTGGTCATTGGTTGATGCAAATGCTTGGGGAGTTTGG 360
CN16_VP2        ACTCATGCACAAATTGTAACACCTTGGTCATTGGTTGATGCAAATGCTTGGGGAGTTTGG 360
CN17_VP2        ACTCATGCACAAATTGTAACACCTTGGTCATTGGTTGATGCAAATGCTTGGGGAGTTTGG 360
CN18_VP2        ACTCATGCACAAATTGTAACACCTTGGTCATTGGTTGATGCAAATGCTTGGGGAGTTTGG 360
CN19_VP2        ACTCATGCACAAATTGTAACACCTTGGTCATTGGTTGATGCAAATGCTTGGGGAGTTTGG 360
CN20_VP2        ACTCATGCACAAATTGTAACACCTTGGTCATTGGTTGATGCAAATGCTTGGGGAGTTTGG 360
CN21_VP2        ACTCATGCACAAATTGTAACACCTTGGTCATTGGTTGATGCAAATGCTTGGGGAGTTTGG 360
CN22_VP2        ACTCATGCACAAATTGTAACACCTTGGTCATTGGTTGATGCAAATGCTTGGGGAGTTTGG 360
CN23_VP2        ACTCATGCACAAATTGTAACACCTTGGTCATTGGTTGATGCAAATGCTTGGGGAGTTTGG 360
CN24_VP2        ACTCATGCACAAATTGTAACACCTTGGTCATTGGTTGATGCAAATGCTTGGGGAGTTTGG 360
CN25_VP2        ACTCATGCACAAATTGTAACACCTTGGTCATTGGTTGATGCAAATGCTTGGGGAGTTTGG 360
CN26_VP2        ACTCATGCACAAATTGTAACACCTTGGTCATTGGTTGATGCAAATGCTTGGGGAGTTTGG 360
CN27_VP2        ACTCATGCACAAATTGTAACACCTTGGTCATTGGTTGATGCAAATGCTTGGGGAGTTTGG 360
CN28_VP2        ACTCATGCACAAATTGTAACACCTTGGTCATTGGTTGATGCAAATGCTTGGGGAGTTTGG 360
CN29_VP2        ACTCATGCACAAATTGTAACACCTTGGTCATTGGTTGATGCAAATGCTTGGGGAGTTTGG 360
CN30_VP2        ACTCATGCACAAATTGTAACACCTTGGTCATTGGTTGATGCAAATGCTTGGGGAGTTTGG 360
CN31_VP2        ACTCATGCACAAATTGTAACACCTTGGTCATTGGTTGATGCAAATGCTTGGGGAGTTTGG 360
CN32_VP2        ACTCATGCACAAATTGTAACACCTTGGTCATTGGTTGATGCAAATGCTTGGGGAGTTTGG 360
CN33_VP2        ACTCATGCACAAATTGTAACACCTTGGTCATTGGTTGATGCAAATGCTTGGGGAGTTTGG 360
CN34_VP2        ACTCATGCACAAATTGTAACACCTTGGTCATTGGTTGATGCAAATGCTTGGGGAGTTTGG 360
CN35_VP2        ACTCATGCACAAATTGTAACACCTTGGTCATTGGTTGATGCAAATGCTTGGGGAGTTTGG 360
CN36_VP2        ACTCATGCACAAATTGTAACACCTTGGTCATTGGTTGATGCAAATGCTTGGGGAGTTTGG 360
CN37_VP2        ACTCATGCACAAATTGTAACACCTTGGTCATTGGTTGATGCAAATGCTTGGGGAGTTTGG 360
CN38_VP2        ACTCATGCACAAATTGTAACACCTTGGTCATTGGTTGATGCAAATGCTTGGGGAGTTTGG 360
CN39_VP2        ACTCATGCACAAATTGTAACACCTTGGTCATTGGTTGATGCAAATGCTTGGGGAGTTTGG 360
CN40_VP2        ACTCATGCACAAATTGTAACACCTTGGTCATTGGTTGATGCAAATGCTTGGGGAGTTTGG 360
CK81_VP2        ACTCATGCACAAATTGTAACACCTTGGTCATTGGTTGATGCAAATGCTTGGGGAGTTTGG 360
CK84_VP2        ACTCATGCACAAATTGTAACACCTTGGTCATTGGTTGATGCAAATGCTTGGGGAGTTTGG 360
RVC50_VP2       ACTCATGCACAAATTGTAACACCTTGGTCATTGGTTGATGCAAATGCTTGGGGAGTTTGG 360
                ************************************************************
```

```
CN7_VP2         TTTAATCCAGGAGATTGGCAACTAATTGTTAATACTATGAGTGAGTTACATTTAGTTAGT 420
CN8_VP2         TTTAATCCAGGAGATTGGCAACTAATTGTTAATACTATGAGTGAGTTACATTTAGTTAGT 420
CN10_VP2        TTTAATCCAGGAGATTGGCAACTAATTGTTAATACTATGAGTGAGTTACATTTAGTTAGT 420
CN11_VP2        TTTAATCCAGGAGATTGGCAACTAATTGTTAATACTATGAGTGAGTTACATTTAGTTAGT 420
CN12_VP2        TTTAATCCAGGAGATTGGCAACTAATTGTTAATACTATGAGTGAGTTACATTTAGTTAGT 420
CN13_VP2        TTTAATCCAGGAGATTGGCAACTAATTGTTAATACTATGAGTGAGTTACATTTAGTTAGT 420
CN14_VP2        TTTAATCCAGGAGATTGGCAACTAATTGTTAATACTATGAGTGAGTTACATTTAGTTAGT 420
CN15_VP2        TTTAATCCAGGAGATTGGCAACTAATTGTTAATACTATGAGTGAGTTACATTTAGTTAGT 420
CN16_VP2        TTTAATCCAGGAGATTGGCAACTAATTGTTAATACTATGAGTGAGTTACATTTAGTTAGT 420
CN17_VP2        TTTAATCCAGGAGATTGGCAACTAATTGTTAATACTATGAGTGAGTTACATTTAGTTAGT 420
CN18_VP2        TTTAATCCAGGAGATTGGCAACTAATTGTTAATACTATGAGTGAGTTACATTTAGTTAGT 420
CN19_VP2        TTTAATCCAGGAGATTGGCAACTAATTGTTAATACTATGAGTGAGTTACATTTAGTTAGT 420
CN20_VP2        TTTAATCCAGGAGATTGGCAACTAATTGTTAATACTATGAGTGAGTTACATTTAGTTAGT 420
CN21_VP2        TTTAATCCAGGAGATTGGCAACTAATTGTTAATACTATGAGTGAGTTACATTTAGTTAGT 420
CN22_VP2        TTTAATCCAGGAGATTGGCAACTAATTGTTAATACTATGAGTGAGTTACATTTAGTTAGT 420
CN23_VP2        TTTAATCCAGGAGATTGGCAACTAATTGTTAATACTATGAGTGAGTTACATTTAGTTAGT 420
CN24_VP2        TTTAATCCAGGAGATTGGCAACTAATTGTTAATACTATGAGTGAGTTACATTTAGTTAGT 420
CN25_VP2        TTTAATCCAGGAGATTGGCAACTAATTGTTAATACTATGAGTGAGTTACATTTAGTTAGT 420
CN26_VP2        TTTAATCCAGGAGATTGGCAACTAATTGTTAATACTATGAGTGAGTTACATTTAGTTAGT 420
CN27_VP2        TTTAATCCAGGAGATTGGCAACTAATTGTTAATACTATGAGTGAGTTACATTTAGTTAGT 420
CN28_VP2        TTTAATCCAGGAGATTGGCAACTAATTGTTAATACTATGAGTGAGTTACATTTAGTTAGT 420
CN29_VP2        TTTAATCCAGGAGATTGGCAACTAATTGTTAATACTATGAGTGAGTTACATTTAGTTAGT 420
CN30_VP2        TTTAATCCAGGAGATTGGCAACTAATTGTTAATACTATGAGTGAGTTACATTTAGTTAGT 420
CN31_VP2        TTTAATCCAGGAGATTGGCAACTAATTGTTAATACTATGAGTGAGTTACATTTAGTTAGT 420
CN32_VP2        TTTAATCCAGGAGATTGGCAACTAATTGTTAATACTATGAGTGAGTTACATTTAGTTAGT 420
CN33_VP2        TTTAATCCAGGAGATTGGCAACTAATTGTTAATACTATGAGTGAGTTACATTTAGTTAGT 420
CN34_VP2        TTTAATCCAGGAGATTGGCAACTAATTGTTAATACTATGAGTGAGTTACATTTAGTTAGT 420
CN35_VP2        TTTAATCCAGGAGATTGGCAACTAATTGTTAATACTATGAGTGAGTTACATTTAGTTAGT 420
CN36_VP2        TTTAATCCAGGAGATTGGCAACTAATTGTTAATACTATGAGTGAGTTACATTTAGTTAGT 420
CN37_VP2        TTTAATCCAGGAGATTGGCAACTAATTGTTAATACTATGAGTGAGTTACATTTAGTTAGT 420
CN38_VP2        TTTAATCCAGGAGATTGGCAACTAATTGTTAATACTATGAGTGAGTTACATTTAGTTAGT 420
CN39_VP2        TTTAATCCAGGAGATTGGCAACTAATTGTTAATACTATGAGTGAGTTACATTTAGTTAGT 420
CN40_VP2        TTTAATCCAGGAGATTGGCAACTAATTGTTAATACTATGAGTGAGTTACATTTAGTTAGT 420
CK81_VP2        TTTAATCCAGGAGATTGGCAACTAATTGTTAATACTATGAGTGAGTTACATTTAGTTAGT 420
CK84_VP2        TTTAATCCAGGAGATTGGCAACTAATTGTTAATACTATGAGTGAGTTACATTTAGTTAGT 420
RVC50_VP2       TTTAATCCAGGAGATTGGCAACTAATTGTTAATACTATGAGTGAGTTACATTTAGTTAGT 420
                ************************************************************

CN7_VP2         TTTGAGCAAGAAATTTTTAATGTTGTTTTAAAGACTGTTTCAGAATCTGCTACTCAGCCA 480
CN8_VP2         TTTGAGCAAGAAATTTTTAATGTTGTTTTAAAGACTGTTTCAGAATCTGCTACTCAGCCA 480
CN10_VP2        TTTGAGCAAGAAATTTTTAATGTTGTTTTAAAGACTGTTTCAGAATCTGCTACTCAGCCA 480
CN11_VP2        TTTGAGCAAGAAATTTTTAATGTTGTTTTAAAGACTGTTTCAGAATCTGCTACTCAGCCA 480
CN12_VP2        TTTGAGCAAGAAATTTTTAATGTTGTTTTAAAGACTGTTTCAGAATCTGCTACTCAGCCA 480
CN13_VP2        TTTGAGCAAGAAATTTTTAATGTTGTTTTAAAGACTGTTTCAGAATCTGCTACTCAGCCA 480
CN14_VP2        TTTGAGCAAGAAATTTTTAATGTTGTTTTAAAGACTGTTTCAGAATCTGCTACTCAGCCA 480
CN15_VP2        TTTGAGCAAGAAATTTTTAATGTTGTTTTAAAGACTGTTTCAGAATCTGCTACTCAGCCA 480
CN16_VP2        TTTGAGCAAGAAATTTTTAATGTTGTTTTAAAGACTGTTTCAGAATCTGCTACTCAGCCA 480
CN17_VP2        TTTGAGCAAGAAATTTTTAATGTTGTTTTAAAGACTGTTTCAGAATCTGCTACTCAGCCA 480
CN18_VP2        TTTGAGCAAGAAATTTTTAATGTTGTTTTAAAGACTGTTTCAGAATCTGCTACTCAGCCA 480
CN19_VP2        TTTGAGCAAGAAATTTTTAATGTTGTTTTAAAGACTGTTTCAGAATCTGCTACTCAGCCA 480
CN20_VP2        TTTGAGCAAGAAATTTTTAATGTTGTTTTAAAGACTGTTTCAGAATCTGCTACTCAGCCA 480
CN21_VP2        TTTGAGCAAGAAATTTTTAATGTTGTTTTAAAGACTGTTTCAGAATCTGCTACTCAGCCA 480
CN22_VP2        TTTGAGCAAGAAATTTTTAATGTTGTTTTAAAGACTGTTTCAGAATCTGCTACTCAGCCA 480
CN23_VP2        TTTGAGCAAGAAATTTTTAATGTTGTTTTAAAGACTGTTTCAGAATCTGCTACTCAGCCA 480
CN24_VP2        TTTGAGCAAGAAATTTTTAATGTTGTTTTAAAGACTGTTTCAGAATCTGCTACTCAGCCA 480
CN25_VP2        TTTGAGCAAGAAATTTTTAATGTTGTTTTAAAGACTGTTTCAGAATCTGCTACTCAGCCA 480
CN26_VP2        TTTGAGCAAGAAATTTTTAATGTTGTTTTAAAGACTGTTTCAGAATCTGCTACTCAGCCA 480
CN27_VP2        TTTGAGCAAGAAATTTTTAATGTTGTTTTAAAGACTGTTTCAGAATCTGCTACTCAGCCA 480
CN28_VP2        TTTGAGCAAGAAATTTTTAATGTTGTTTTAAAGACTGTTTCAGAATCTGCTACTCAGCCA 480
CN29_VP2        TTTGAGCAAGAAATTTTTAATGTTGTTTTAAAGACTGTTTCAGAATCTGCTACTCAGCCA 480
CN30_VP2        TTTGAGCAAGAAATTTTTAATGTTGTTTTAAAGACTGTTTCAGAATCTGCTACTCAGCCA 480
CN31_VP2        TTTGAGCAAGAAATTTTTAATGTTGTTTTAAAGACTGTTTCAGAATCTGCTACTCAGCCA 480
CN32_VP2        TTTGAGCAAGAAATTTTTAATGTTGTTTTAAAGACTGTTTCAGAATCTGCTACTCAGCCA 480
CN33_VP2        TTTGAGCAAGAAATTTTTAATGTTGTTTTAAAGACTGTTTCAGAATCTGCTACTCAGCCA 480
CN34_VP2        TTTGAGCAAGAAATTTTTAATGTTGTTTTAAAGACTGTTTCAGAATCTGCTACTCAGCCA 480
CN35_VP2        TTTGAGCAAGAAATTTTTAATGTTGTTTTAAAGACTGTTTCAGAATCTGCTACTCAGCCA 480
CN36_VP2        TTTGAGCAAGAAATTTTTAATGTTGTTTTAAAGACTGTTTCAGAATCTGCTACTCAGCCA 480
CN37_VP2        TTTGAGCAAGAAATTTTTAATGTTGTTTTAAAGACTGTTTCAGAATCTGCTACTCAGCCA 480
CN38_VP2        TTTGAGCAAGAAATTTTTAATGTTGTTTTAAAGACTGTTTCAGAATCTGCTACTCAGCCA 480
CN39_VP2        TTTGAGCAAGAAATTTTTAATGTTGTTTTAAAGACTGTTTCAGAATCTGCTACTCAGCCA 480
CN40_VP2        TTTGAGCAAGAAATTTTTAATGTTGTTTTAAAGACTGTTTCAGAATCTGCTACTCAGCCA 480
CK81_VP2        TTTGAGCAAGAAATTTTTAATGTTGTTTTAAAGACTGTTTCAGAATCTGCTACTCAGCCA 480
CK84_VP2        TTTGAGCAAGAAATTTTTAATGTTGTTTTAAAGACTGTTTCAGAATCTGCTACTCAGCCA 480
RVC50_VP2       TTTGAGCAAGAAATTTTTAATGTTGTTTTAAAGACTGTTTCAGAATCTGCTACTCAGCCA 480
                ************************************************************
```

```
CN7_VP2         CCAACTAAAGTTTATAATAATGATTTAACTGCATCATTGATGGTTGCATTAGATAGTAAT 540
CN8_VP2         CCAACTAAAGTTTATAATAATGATTTAACTGCATCATTGATGGTTGCATTAGATAGTAAT 540
CN10_VP2        CCAACTAAAGTTTATAATAATGATTTAACTGCATCATTGATGGTGGCATTAGATAGTAAT 540
CN11_VP2        CCAACTAAAGTTTATAATAATGATTTAACTGCATCATTGATGGTTGCATTAGATAGTAAT 540
CN12_VP2        CCAACTAAAGTTTATAATAATGATTTAACTGCATCATTGATGGTTGCATTAGATAGTAAT 540
CN13_VP2        CCAACTAAAGTTTATAATAATGATTTAACTGCATCATTGATGGTTGCATTAGATAGTAAT 540
CN14_VP2        CCAACTAAAGTTTATAATAATGATTTAACTGCATCATTGATGGTTGCATTAGATAGTAAT 540
CN15_VP2        CCAACTAAAGTTTATAATAATGATTTAACTGCATCATTGATGGTTGCATTAGATAGTAAT 540
CN16_VP2        CCAACTAAAGTTTATAATAATGATTTAACTGCATCATTGATGGTTGCATTAGATAGTAAT 540
CN17_VP2        CCAACTAAAGTTTATAATAATGATTTAACTGCATCATTGATGGTTGCATTAGATAGTAAT 540
CN18_VP2        CCAACTAAAGTTTATAATAATGATTTAACTGCATCATTGATGGTTGCATTAGATAGTAAT 540
CN19_VP2        CCAACTAAAGTTTATAATAATGATTTAACTGCATCATTGATGGTTGCATTAGATAGTAAT 540
CN20_VP2        CCAACTAAAGTTTATAATAATGATTTAACTGCATCATTGATGGTTGCATTAGATAGTAAT 540
CN21_VP2        CCAACTAAAGTTTATAATAATGATTTAACTGCATCATTGATGGTTGCATTAGATAGTAAT 540
CN22_VP2        CCAACTAAAGTTTATAATAATGATTTAACTGCATCATTGATGGTTGCATTAGATAGTAAT 540
CN23_VP2        CCAACTAAAGTTTATAATAATGATTTAACTGCATCATTGATGGTTGCATTAGATAGTAAT 540
CN24_VP2        CCAACTAAAGTTTATAATAATGATTTAACTGCATCATTGATGGTTGCATTAGATAGTAAT 540
CN25_VP2        CCAACTAAAGTTTATAATAATGATTTAACTGCATCATTGATGGTTGCATTAGATAGTAAT 540
CN26_VP2        CCAACTAAAGTTTATAATAATGATTTAACTGCATCATTGATGGTTGCATTAGATAGTAAT 540
CN27_VP2        CCAACTAAAGTTTATAATAATGATTTAACTGCATCATTGATGGTTGCATTAGATAGTAAT 540
CN28_VP2        CCAACTAAAGTTTATAATAATGATTTAACTGCATCATTGATGGTTGCATTAGATAGTAAT 540
CN29_VP2        CCAACTAAAGTTTATAATAATGATTTAACTGCATCATTGATGGTTGCATTAGATAGTAAT 540
CN30_VP2        CCAACTAAAGTTTATAATAATGATTTAACTGCATCATTGATGGTTGCATTAGATAGTAAT 540
CN31_VP2        CCAACTAAAGTTTATAATAATGATTTAACTGCATCATTGATGGTTGCATTAGATAGTAAT 540
CN32_VP2        CCAACTAAAGTTTATAATAATGATTTAACTGCATCATTGATGGTTGCATTAGATAGTAAT 540
CN33_VP2        CCAACTAAAGTTTATAATAATGATTTAACTGCATCATTGATGGTTGCATTAGATAGTAAT 540
CN34_VP2        CCAACTAAAGTTTATAATAATGATTTAACTGCATCATTGATGGTTGCATTAGATAGTAAT 540
CN35_VP2        CCAACTAAAGTTTATAATAATGATTTAACTGCATCATTGATGGTTGCATTAGATAGTAAT 540
CN36_VP2        CCAACTAAAGTTTATAATAATGATTTAACTGCATCATTGATGGTTGCATTAGATAGTAAT 540
CN37_VP2        CCAACTAAAGTTTATAATAATGATTTAACTGCATCATTGATGGTTGCATTAGATAGTAAT 540
CN38_VP2        CCAACTAAAGTTTATAATAATGATTTAACTGCATCATTGATGGTTGCATTAGATAGTAAT 540
CN39_VP2        CCAACTAAAGTTTATAATAATGATTTAACTGCATCATTGATGGTTGCATTAGATAGTAAT 540
CN40_VP2        CCAACTAAAGTTTATAATAATGATTTAACTGCATCATTGATGGTTGCATTAGATAGTAAT 540
CK81_VP2        CCAACTAAAGTTTATAATAATGATTTAACTGCATCATTGATGGTTGCATTAGATAGTAAT 540
CK84_VP2        CCAACTAAAGTTTATAATAATGATTTAACTGCATCATTGATGGTTGCATTAGATAGTAAT 540
RVC50_VP2       CCAACTAAAGTTTATAATAATGATTTAACTGCATCATTGATGGTTGCATTAGATAGTAAT 540
                ******************************************** ***************

CN7_VP2         AATACTATGCCATTTACTCCAGCAGCTATGAGATCTGAGACATTGGGTTTTTATCCATGG 600
CN8_VP2         AATACTATGCCATTTACTCCAGCAGCTATGAGATCTGAAACATTGGGTTTTTATCCATGG 600
CN10_VP2        AATACTATGCCATTTACTCCAGCAGCTATGAGATCTGAGACATTGGGTTTTTATCCATGG 600
CN11_VP2        AATACTATGCCATTTACTCCAGCAGCTATGAGATCTGAGACATTGGGTTTTTATCCATGG 600
CN12_VP2        AATACTATGCCATTTACTCCAGCAGCTATGAGATCTGAAACATTGGGTTTTTATCCATGG 600
CN13_VP2        AATACTATGCCATTTACTCCAGCAGCTATGAGATCTGAAACATTGGGTTTTTATCCATGG 600
CN14_VP2        AATACTATGCCATTTACTCCAGCAGCTATGAGATCTGAGACATTGGGTTTTTATCCATGG 600
CN15_VP2        AATACTATGCCATTTACTCCAGCAGCTATGAGATCTGAGACATTGGGTTTTTATCCATGG 600
CN16_VP2        AATACTATGCCATTTACTCCAGCAGCTATGAGATCTGAAACATTGGGTTTTTATCCATGG 600
CN17_VP2        AATACTATGCCATTTACTCCAGCAGCTATGAGATCTGAGACATTGGGTTTTTATCCATGG 600
CN18_VP2        AATACTATGCCATTTACTCCAGCAGCTATGAGATCTGAAACATTGGGTTTTTATCCATGG 600
CN19_VP2        AATACTATGCCATTTACTCCAGCAGCTATGAGATCTGAGACATTGGGTTTTTATCCATGG 600
CN20_VP2        AATACTATGCCATTTACTCCAGCAGCTATGAGATCTGAGACATTGGGTTTTTATCCATGG 600
CN21_VP2        AATACTATGCCATTTACTCCAGCAGCTATGAGATCTGAGACATTGGGTTTTTATCCATGG 600
CN22_VP2        AATACTATGCCATTTACTCCAGCAGCTATGAGATCTGAGACATTGGGTTTTTATCCATGG 600
CN23_VP2        AATACTATGCCATTTACTCCAGCAGCTATGAGATCTGAGACATTGGGTTTTTATCCATGG 600
CN24_VP2        AATACTATGCCATTTACTCCAGCAGCTATGAGATCTGAGACATTGGGTTTTTATCCATGG 600
CN25_VP2        AATACTATGCCATTTACTCCAGCAGCTATGAGATCTGAGACATTGGGTTTTTATCCATGG 600
CN26_VP2        AATACTATGCCATTTACTCCAGCAGCTATGAGATCTGAGACATTGGGTTTTTATCCATGG 600
CN27_VP2        AATACTATGCCATTTACTCCAGCAGCTATGAGATCTGAGACATTGGGTTTTTATCCATGG 600
CN28_VP2        AATACTATGCCATTTACTCCAGCAGCTATGAGATCTGAGACATTGGGTTTTTATCCATGG 600
CN29_VP2        AATACTATGCCATTTACTCCAGCAGCTATGAGATCTGAAACATTGGGTTTTTATCCATGG 600
CN30_VP2        AATACTATGCCATTTACTCCAGCAGCTATGAGATCTGAGACATTGGGTTTTTATCCATGG 600
CN31_VP2        AATACTATGCCATTTACTCCAGCAGCTATGAGATCTGAGACATTGGGTTTTTATCCATGG 600
CN32_VP2        AATACTATGCCATTTACTCCAGCAGCTATGAGATCTGAGACATTGGGTTTTTATCCATGG 600
CN33_VP2        AATACTATGCCATTTACTCCAGCAGCTATGAGATCTGAGACATTGGGTTTTTATCCATGG 600
CN34_VP2        AATACTATGCCATTTACTCCAGCAGCTATGAGATCTGAAACATTGGGTTTTTATCCATGG 600
CN35_VP2        AATACTATGCCATTTACTCCAGCAGCTATGAGATCTGAGACATTGGGTTTTTATCCATGG 600
CN36_VP2        AATACTATGCCATTTACTCCAGCAGCTATGAGATCTGAGACATTGGGTTTTTATCCATGG 600
CN37_VP2        AATACTATGCCATTTACTCCAGCAGCTATGAGATCTGAGACATTGGGTTTTTATCCATGG 600
CN38_VP2        AATACTATGCCATTTACTCCAGCAGCTATGAGATCTGAGACATTGGGTTTTTATCCATGG 600
CN39_VP2        AATACTATGCCATTTACTCCAGCAGCTATGAGATCTGAAACATTGGGTTTTTATCCATGG 600
CN40_VP2        AATACTATGCCATTTACTCCAGCAGCTATGAGATCTGAGACATTGGGTTTTTATCCATGG 600
CK81_VP2        AATACTATGCCATTTACTCCAGCAGCTATGAGATCTGAGACATTGGGTTTTTATCCATGG 600
CK84_VP2        AATACTATGCCATTTACTCCAGCAGCTATGAGATCTGAGACATTGGGTTTTTATCCATGG 600
RVC50_VP2       AATACTATGCCATTTACTCCAGCAGCTATGAGATCTGAGACATTGGGTTTTTATCCATGG 600
                ************************************** *********************
```

```
CN7_VP2         AAACCAACCATACCAACTCCATGGAGATATTATTTTCAATGGGATAGAACATTAATACCA 660
CN8_VP2         AAACCAACCATACCAACTCCATGGAGATATTATTTTCAATGGGATAGAACATTAATACCA 660
CN10_VP2        AAGCCAACCATACCAACTCCATGGAGATATTATTTTCAATGGGATAGAACATTAATACCA 660
CN11_VP2        AAACCAACCATACCAACTCCATGGAGATATTATTTTCAATGGGATAGAACATTAATACCA 660
CN12_VP2        AAACCAACCATACCAACTCCATGGAGATATTATTTTCAATGGGATAGAACATTAATACCA 660
CN13_VP2        AAACCAACCATACCAACTCCATGGAGATATTATTTTCAATGGGATAGAACATTAATACCA 660
CN14_VP2        AAACCAACCATACCAACTCCATGGAGATATTATTTTCAATGGGATAGAACATTAATACCA 660
CN15_VP2        AAACCAACCATACCAACTCCATGGAGATATTATTTTCAATGGGATAGAACATTAATACCA 660
CN16_VP2        AAACCAACCATACCAACTCCATGGAGATATTATTTTCAATGGGATAGAACATTAATACCA 660
CN17_VP2        AAACCAACCATACCAACTCCATGGAGATATTATTTTCAATGGGATAGAACATTAATACCA 660
CN18_VP2        AAACCAACCATACCAACTCCATGGAGATATTATTTTCAATGGGATAGAACATTAATACCA 660
CN19_VP2        AAACCAACCATACCAACTCCATGGAGATATTATTTTCAATGGGATAGAACATTAATACCA 660
CN20_VP2        AAACCAACCATACCAACTCCATGGAGATATTATTTTCAATGGGATAGAACATTAATACCA 660
CN21_VP2        AAACCAACCATACCAACTCCATGGAGATATTATTTTCAATGGGATAGAACATTAATACCA 660
CN22_VP2        AAACCAACCATACCAACTCCATGGAGATATTATTTTCAATGGGATAGAACATTAATACCA 660
CN23_VP2        AAACCAACCATACCAACTCCATGGAGATATTATTTTCAATGGGATAGAACATTAATACCA 660
CN24_VP2        AAACCAACCATACCAACTCCATGGAGATATTATTTTCAATGGGATAGAACATTAATACCA 660
CN25_VP2        AAACCAACCATACCAACTCCATGGAGATATTATTTTCAATGGGATAGAACATTAATACCA 660
CN26_VP2        AAACCAACCATACCAACTCCATGGAGATATTATTTTCAATGGGATAGAACATTAATACCA 660
CN27_VP2        AAACCAACCATACCAACTCCATGGAGATATTATTTTCAATGGGATAGAACATTAATACCA 660
CN28_VP2        AAACCAACCATACCAACTCCATGGAGATATTATTTTCAATGGGATAGAACATTAATACCA 660
CN29_VP2        AAACCAACCATACCAACTCCATGGAGATATTATTTTCAATGGGATAGAACATTAATACCA 660
CN30_VP2        AAACCAACCATACCAACTCCATGGAGATATTATTTTCAATGGGATAGAACATTAATACCA 660
CN31_VP2        AAACCAACCATACCAACTCCATGGAGATATTATTTTCAATGGGATAGAACATTAATACCA 660
CN32_VP2        AAACCAACCATACCAACTCCATGGAGATATTATTTTCAATGGGATAGAACATTAATACCA 660
CN33_VP2        AAACCAACCATACCAACTCCATGGAGATATTATTTTCAATGGGATAGAACATTAATACCA 660
CN34_VP2        AAACCAACCATACCAACTCCATGGAGATATTATTTTCAATGGGATAGAACATTAATACCA 660
CN35_VP2        AAACCAACCATACCAACTCCATGGAGATATTATTTTCAATGGGATAGAACATTAATACCA 660
CN36_VP2        AAACCAACCATACCAACTCCATGGAGATATTATTTTCAATGGGATAGAACATTAATACCA 660
CN37_VP2        AAACCAACCATACCAACTCCATGGAGATATTATTTTCAATGGGATAGAACATTAATACCA 660
CN38_VP2        AAACCAACCATACCAACTCCATGGAGATATTATTTTCAATGGGATAGAACATTAATACCA 660
CN39_VP2        AAACCAACCATACCAACTCCATGGAGATATTATTTTCAATGGGATAGAACATTAATACCA 660
CN40_VP2        AAACCAACCATACCAACTCCATGGAGATATTATTTTCAATGGGATAGAACATTAATACCA 660
CK81_VP2        AAACCAACCATACCAACTCCATGGAGATATTATTTTCAATGGGATAGAACATTAATACCA 660
CK84_VP2        AAACCAACCATACCAACTCCATGGAGATATTATTTTCAATGGGATAGAACATTAATACCA 660
RVC50_VP2       AAACCAACCATACCAACTCCATGGAGATATTATTTTCAATGGGATAGAACATTAATACCA 660
                ** *********************************************************

CN7_VP2         TCTCATACTGGAACTAGTGGCACACCAACAAATATATACCATGGTACAGATCCAGATGAT 720
CN8_VP2         TCTCATACTGGAACTAGTGGCACACCAACAAATATATACCATGGTACAGATCCAGATGAT 720
CN10_VP2        TCTCATACTGGAACTAGTGGCACACCAACAAATATATACCATGGTACAGATCCAGATGAT 720
CN11_VP2        TCTCATACTGGAACTAGTGGCACACCAACAAATATATACCATGGTACAGATCCAGATGAT 720
CN12_VP2        TCTCATACTGGAACTAGTGGCACACCAACAAATATATACCATGGTACAGATCCAGATGAT 720
CN13_VP2        TCTCATACTGGAACTAGTGGCACACCAACAAATATATACCATGGTACAGATCCAGATGAT 720
CN14_VP2        TCTCATACTGGAACTAGTGGCACACCAACAAATATATACCATGGTACAGATCCAGATGAT 720
CN15_VP2        TCTCATACTGGAACTAGTGGCACACCAACAAATATATACCATGGTACAGATCCAGATGAT 720
CN16_VP2        TCTCATACTGGAACTAGTGGCACACCAACAAATATATACCATGGTACAGATCCAGATGAT 720
CN17_VP2        TCTCATACTGGAACTAGTGGCACACCAACAAATATATACCATGGTACAGATCCAGATGAT 720
CN18_VP2        TCTCATACTGGAACTAGTGGCACACCAACAAATATATACCATGGTACAGATCCAGATGAT 720
CN19_VP2        TCTCATACTGGAACTAGTGGCACACCAACAAATATATACCATGGTACAGATCCAGATGAT 720
CN20_VP2        TCTCATACTGGAACTAGTGGCACACCAACAAATATATACCATGGTACAGATCCAGATGAT 720
CN21_VP2        TCTCATACTGGAACTAGTGGCACACCAACAAATATATACCATGGTACAGATCCAGATGAT 720
CN22_VP2        TCTCATACTGGAACTAGTGGCACACCAACAAATATATACCATGGTACAGATCCAGATGAT 720
CN23_VP2        TCTCATACTGGAACTAGTGGCACACCAACAAATATATACCATGGTACAGATCCAGATGAT 720
CN24_VP2        TCTCATACTGGAACTAGTGGCACACCAACAAATATATACCATGGTACAGATCCAGATGAT 720
CN25_VP2        TCTCATACTGGAACTAGTGGCACACCAACAAATATATACCATGGTACAGATCCAGATGAT 720
CN26_VP2        TCTCATACTGGAACTAGTGGCACACCAACAAATATATACCATGGTACAGATCCAGATGAT 720
CN27_VP2        TCTCATACTGGAACTAGTGGCACACCAACAAATATATACCATGGTACAGATCCAGATGAT 720
CN28_VP2        TCTCATACTGGAACTAGTGGCACACCAACAAATATATACCATGGTACAGATCCAGATGAT 720
CN29_VP2        TCTCATACTGGAACTAGTGGCACACCAACAAATATATACCATGGTACAGATCCAGATGAT 720
CN30_VP2        TCTCATACTGGAACTAGTGGCACACCAACAAATATATACCATGGTACAGATCCAGATGAT 720
CN31_VP2        TCTCATACTGGAACTAGTGGCACACCAACAAATATATACCATGGTACAGATCCAGATGAT 720
CN32_VP2        TCTCATACTGGAACTAGTGGCACACCAACAAATATATACCATGGTACAGATCCAGATGAT 720
CN33_VP2        TCTCATACTGGAACTAGTGGCACACCAACAAATATATACCATGGTACAGATCCAGATGAT 720
CN34_VP2        TCTCATACTGGAACTAGTGGCACACCAACAAATATATACCATGGTACAGATCCAGATGAT 720
CN35_VP2        TCTCATACTGGAACTAGTGGCACACCAACAAATATATACCATGGTACAGATCCAGATGAT 720
CN36_VP2        TCTCATACTGGAACTAGTGGCACACCAACAAATATATACCATGGTACAGATCCAGATGAT 720
CN37_VP2        TCTCATACTGGAACTAGTGGCACACCAACAAATATATACCATGGTACAGATCCAGATGAT 720
CN38_VP2        TCTCATACTGGAACTAGTGGCACACCAACAAATATATACCATGGTACAGATCCAGATGAT 720
CN39_VP2        TCTCATACTGGAACTAGTGGCACACCAACAAATATATACCATGGTACAGATCCAGATGAT 720
CN40_VP2        TCTCATACTGGAACTAGTGGCACACCAACAAATATATACCATGGTACAGATCCAGATGAT 720
CK81_VP2        TCTCATACTGGAACTAGTGGCACACCAACAAATATATACCATGGTACAGATCCAGATGAT 720
CK84_VP2        TCTCATACTGGAACTAGTGGCACACCAACAAATATATACCATGGTACAGATCCAGATGAT 720
RVC50_VP2       TCTCATACTGGAACTAGTGGCACACCAACAAATATATACCATGGTACAGATCCAGATGAT 720
                ************************************************************
```

```
CN7_VP2         GTTCAATTTTATACTATTGAAAATTCTGTGCCAGTACACTTACTAAGAACAGGTGATGAA 780
CN8_VP2         GTTCAATTTTATACTATTGAAAATTCTGTGCCAGTACACTTACTAAGAACAGGTGATGAA 780
CN10_VP2        GTTCAATTTTATACTATTGAAAATTCTGTGCCAGTACACTTACTAAGAACAGGTGATGAA 780
CN11_VP2        GTTCAATTTTATACTATTGAAAATTCTGTGCCAGTACACTTACTAAGAACAGGTGATGAA 780
CN12_VP2        GTTCAATTTTATACTATTGAAAATTCTGTGCCAGTACACTTACTAAGAACAGGTGATGAA 780
CN13_VP2        GTTCAATTTTATACTATTGAAAATTCTGTGCCAGTACACTTACTAAGAACAGGTGATGAA 780
CN14_VP2        GTTCAATTTTATACTATTGAAAATTCTGTGCCAGTACACTTACTAAGAACAGGTGATGAA 780
CN15_VP2        GTTCAATTTTATACTATTGAAAATTCTGTGCCAGTACACTTACTAAGAACAGGTGATGAA 780
CN16_VP2        GTTCAATTTTATACTATTGAAAATTCTGTGCCAGTACACTTACTAAGAACAGGTGATGAA 780
CN17_VP2        GTTCAATTTTATACTATTGAAAATTCTGTGCCAGTACACTTACTAAGAACAGGTGATGAA 780
CN18_VP2        GTTCAATTTTATACTATTGAAAATTCTGTGCCAGTACACTTACTAAGAACAGGTGATGAA 780
CN19_VP2        GTTCAATTTTATACTATTGAAAATTCTGTGCCAGTACACTTACTAAGAACAGGTGATGAA 780
CN20_VP2        GTTCAATTTTATACTATTGAAAATTCTGTGCCAGTACACTTACTAAGAACAGGTGATGAA 780
CN21_VP2        GTTCAATTTTATACTATTGAAAATTCTGTGCCAGTACACTTACTAAGAACAGGTGATGAA 780
CN22_VP2        GTTCAATTTTATACTATTGAAAATTCTGTGCCAGTACACTTACTAAGAACAGGTGATGAA 780
CN23_VP2        GTTCAATTTTATACTATTGAAAATTCTGTGCCAGTACACTTACTAAGAACAGGTGATGAA 780
CN24_VP2        GTTCAATTTTATACTATTGAAAATTCTGTGCCAGTACACTTACTAAGAACAGGTGATGAA 780
CN25_VP2        GTTCAATTTTATACTATTGAAAATTCTGTGCCAGTACACTTACTAAGAACAGGTGATGAA 780
CN26_VP2        GTTCAATTTTATACTATTGAAAATTCTGTGCCAGTACACTTACTAAGAACAGGTGATGAA 780
CN27_VP2        GTTCAATTTTATACTATTGAAAATTCTGTGCCAGTACACTTACTAAGAACAGGTGATGAA 780
CN28_VP2        GTTCAATTTTATACTATTGAAAATTCTGTGCCAGTACACTTACTAAGAACAGGTGATGAA 780
CN29_VP2        GTTCAATTTTATACTATTGAAAATTCTGTGCCAGTACACTTACTAAGAACAGGTGATGAA 780
CN30_VP2        GTTCAATTTTATACTATTGAAAATTCTGTGCCAGTACACTTACTAAGAACAGGTGATGAA 780
CN31_VP2        GTTCAATTTTATACTATTGAAAATTCTGTGCCAGTACACTTACTAAGAACAGGTGATGAA 780
CN32_VP2        GTTCAATTTTATACTATTGAAAATTCTGTGCCAGTACACTTACTAAGAACAGGTGATGAA 780
CN33_VP2        GTTCAATTTTATACTATTGAAAATTCTGTGCCAGTACACTTACTAAGAACAGGTGATGAA 780
CN34_VP2        GTTCAATTTTATACTATTGAAAATTCTGTGCCAGTACACTTACTAAGAACAGGTGATGAA 780
CN35_VP2        GTTCAATTTTATACTATTGAAAATTCTGTGCCAGTACACTTACTAAGAACAGGTGATGAA 780
CN36_VP2        GTTCAATTTTATACTATTGAAAATTCTGTGCCAGTACACTTACTAAGAACAGGTGATGAA 780
CN37_VP2        GTTCAATTTTATACTATTGAAAATTCTGTGCCAGTACACTTACTAAGAACAGGTGATGAA 780
CN38_VP2        GTTCAATTTTATACTATTGAAAATTCTGTGCCAGTACACTTACTAAGAACAGGTGATGAA 780
CN39_VP2        GTTCAATTTTATACTATTGAAAATTCTGTGCCAGTACACTTACTAAGAACAGGTGATGAA 780
CN40_VP2        GTTCAATTTTATACTATTGAAAATTCTGTGCCAGTACACTTACTAAGAACAGGTGATGAA 780
CK81_VP2        GTTCAATTTTATACTATTGAAAATTCTGTGCCAGTACACTTACTAAGAACAGGTGATGAA 780
CK84_VP2        GTTCAATTTTATACTATTGAAAATTCTGTGCCAGTACACTTACTAAGAACAGGTGATGAA 780
RVC50_VP2       GTTCAATTTTATACTATTGAAAATTCTGTGCCAGTACACTTACTAAGAACAGGTGATGAA 780
                ************************************************************

CN7_VP2         TTTGCTACAGGAACATTTTTTTTTGATTGTAAACCATGTAGACTAACACATACATGGCAA 840
CN8_VP2         TTTGCTACAGGAACATTTTTTTTTGATTGTAAACCATGTAGACTAACACATACATGGCAA 840
CN10_VP2        TTTGCTACAGGAACATTTTTTTTTGATTGTAAACCATGTAGACTAACACATACATGGCAA 840
CN11_VP2        TTTGCTACAGGAACATTTTTTTTTGATTGTAAACCATGTAGACTAACACATACATGGCAA 840
CN12_VP2        TTTGCTACAGGAACATTTTTTTTTGATTGTAAACCATGTAGACTAACACATACATGGCAA 840
CN13_VP2        TTTGCTACAGGAACATTTTTTTTTGATTGTAAACCATGTAGACTAACACATACATGGCAA 840
CN14_VP2        TTTGCTACAGGAACATTTTTTTTTGATTGTAAACCATGTAGACTAACACATACATGGCAA 840
CN15_VP2        TTTGCTACAGGAACATTTTTTTTTGATTGTAAACCATGTAGACTAACACATACATGGCAA 840
CN16_VP2        TTTGCTACAGGAACATTTTTTTTTGATTGTAAACCATGTAGACTAACACATACATGGCAA 840
CN17_VP2        TTTGCTACAGGAACATTTTTTTTTGATTGTAAACCATGTAGACTAACACATACATGGCAA 840
CN18_VP2        TTTGCTACAGGAACATTTTTTTTTGATTGTAAACCATGTAGACTAACACATACATGGCAA 840
CN19_VP2        TTTACTACAGGAACATTTTTTTTTGATTGTAAACCATGTAGACTAACACATACATGGCAA 840
CN20_VP2        TTTACTACAGGAACATTTTTTTTTGATTGTAAACCATGTAGACTAACACATACATGGCAA 840
CN21_VP2        TTTGCTACAGGAACATTTTTTTTTGATTGTAAACCATGTAGACTAACACATACATGGCAA 840
CN22_VP2        TTTGCTACAGGAACATTTTTTTTTGATTGTAAACCATGTAGACTAACACATACATGGCAA 840
CN23_VP2        TTTGCTACAGGAACATTTTTTTTTGATTGTAAACCATGTAGACTAACACATACATGGCAA 840
CN24_VP2        TTTACTACAGGAACATTTTTTTTTGATTGTAAACCATGTAGACTAACACATACATGGCAA 840
CN25_VP2        TTTGCTACAGGAACATTTTTTTTTGATTGTAAACCATGTAGACTAACACATACATGGCAA 840
CN26_VP2        TTTGCTACAGGAACATTTTTTTTTGATTGTAAACCATGTAGACTAACACATACATGGCAA 840
CN27_VP2        TTTACTACAGGAACATTTTTTTTTGATTGTAAACCATGTAGACTAACACATACATGGCAA 840
CN28_VP2        TTTGCTACAGGAACATTTTTTTTTGATTGTAAACCATGTAGACTAACACATACATGGCAA 840
CN29_VP2        TTTGCTACAGGAACATTTTTTTTTGATTGTAAACCATGTAGACTAACACATACATGGCAA 840
CN30_VP2        TTTGCTACAGGAACATTTTTTTTTGATTGTAAACCATGTAGACTAACACATACATGGCAA 840
CN31_VP2        TTTGCTACAGGAACATTTTTTTTTGATTGTAAACCATGTAGACTAACACATACATGGCAA 840
CN32_VP2        TTTGCTACAGGAACATTTTTTTTTGATTGTAAACCATGTAGACTAACACATACATGGCAA 840
CN33_VP2        TTTGCTACAGGAACATTTTTTTTTGATTGTAAACCATGTAGACTAACACATACATGGCAA 840
CN34_VP2        TTTGCTACAGGAACATTTTTTTTTGATTGTAAACCATGTAGACTAACACATACATGGCAA 840
CN35_VP2        TTTGCTACAGGAACATTTTTTTTTGATTGTAAACCATGTAGACTAACACATACATGGCAA 840
CN36_VP2        TTTGCTACAGGAACATTTTTTTTTGATTGTAAACCATGTAGACTAACACATACATGGCAA 840
CN37_VP2        TTTGCTACAGGAACATTTTTTTTTGATTGTAAACCATGTAGACTAACACATACATGGCAA 840
CN38_VP2        TTTGCTACAGGAACATTTTTTTTTGATTGTAAACCATGTAGACTAACACATACATGGCAA 840
CN39_VP2        TTTGCTACAGGAACATTTTTTTTTGATTGTAAACCATGTAGACTAACACATACATGGCAA 840
CN40_VP2        TTTGCTACAGGAACATTTTTTTTTGATTGTAAACCATGTAGACTAACACATACATGGCAA 840
CK81_VP2        TTTGCTACAGGAACATTTTTTTTTGATTGTAAACCATGTAGACTAACACATACATGGCAA 840
CK84_VP2        TTTGCTACAGGAACATTTTTTTTTGATTGTAAACCATGTAGACTAACACATACATGGCAA 840
RVC50_VP2       TTTGCTACAGGAACATTTTTTTTTGATTGTAAACCATGTAGACTAACACATACATGGCAA 840
                *** ********************************************************
```

```
CN7_VP2         ACAAATAGAGCATTGGGCTTACCACCATTTCTAAATTCTTTGCCTCAAGCTGAAGGAGGT 900
CN8_VP2         ACAAATAGAGCATTGGGCTTACCACCATTTCTAAATTCTTTGCCTCAAGCTGAAGGAGGT 900
CN10_VP2        ACAAATAGAGCATTGGGCTTACCACCATTTCTAAATTCTTTGCCTCAAGCTGAAGGAGGT 900
CN11_VP2        ACAAATAGAGCATTGGGCTTACCACCATTTCTAAATTCTTTGCCTCAAGCTGAAGGAGGT 900
CN12_VP2        ACAAATAGAGCATTGGGCTTACCACCATTTCTAAATTCTTTGCCTCAAGCTGAAGGAGGT 900
CN13_VP2        ACAAATAGAGCATTGGGCTTACCACCATTTCTAAATTCTTTGCCTCAAGCTGAAGGAGGT 900
CN14_VP2        ACAAATAGAGCATTGGGCTTACCACCATTTCTAAATTCTTTGCCTCAAGCTGAAGGAGGT 900
CN15_VP2        ACAAATAGAGCATTGGGCTTACCACCATTTCTAAATTCTTTGCCTCAAGCTGAAGGAGGT 900
CN16_VP2        ACAAATAGAGCATTGGGCTTACCACCATTTCTAAATTCTTTGCCTCAAGCTGAAGGAGGT 900
CN17_VP2        ACAAATAGAGCATTGGGCTTACCACCATTTCTAAATTCTTTGCCTCAAGCTGAAGGAGGT 900
CN18_VP2        ACAAATAGAGCATTGGGCTTACCACCATTTCTAAATTCTTTGCCTCAAGCTGAAGGAGGT 900
CN19_VP2        ACAAATAGAGCATTGGGCTTACCACCATTTCTAAATTCTTTGCCTCAAGCTGAAGGAGGT 900
CN20_VP2        ACAAATAGAGCATTGGGCTTACCACCATTTCTAAATTCTTTGCCTCAAGCTGAAGGAGGT 900
CN21_VP2        ACAAATAGAGCATTGGGCTTACCACCATTTCTAAATTCTTTGCCTCAAGCTGAAGGAGGT 900
CN22_VP2        ACAAATAGAGCATTGGGCTTACCACCATTTCTAAATTCTTTGCCTCAAGCTGAAGGAGGT 900
CN23_VP2        ACAAATAGAGCATTGGGCTTACCACCATTTCTAAATTCTTTGCCTCAAGCTGAAGGAGGT 900
CN24_VP2        ACAAATAGAGCATTGGGCTTACCACCATTTCTAAATTCTTTGCCTCAAGCTGAAGGAGGT 900
CN25_VP2        ACAAATAGAGCATTGGGCTTACCACCATTTCTAAATTCTTTGCCTCAAGCTGAAGGAGGT 900
CN26_VP2        ACAAATAGAGCATTGGGCTTACCACCATTTCTAAATTCTTTGCCTCAAGCTGAAGGAGGT 900
CN27_VP2        ACAAATAGAGCATTGGGCTTACCACCATTTCTAAATTCTTTGCCTCAAGCTGAAGGAGGT 900
CN28_VP2        ACAAATAGAGCATTGGGCTTACCACCATTTCTAAATTCTTTGCCTCAAGCTGAAGGAGGT 900
CN29_VP2        ACAAATAGAGCATTGGGCTTACCACCATTTCTAAATTCTTTGCCTCAAGCTGAAGGAGGT 900
CN30_VP2        ACAAATAGAGCATTGGGCTTACCACCATTTCTAAATTCTTTGCCTCAAGCTGAAGGAGGT 900
CN31_VP2        ACAAATAGAGCATTGGGCTTACCACCATTTCTAAATTCTTTGCCTCAAGCTGAAGGAGGT 900
CN32_VP2        ACAAATAGAGCATTGGGCTTACCACCATTTCTAAATTCTTTGCCTCAAGCTGAAGGAGGT 900
CN33_VP2        ACAAATAGAGCATTGGGCTTACCACCATTTCTAAATTCTTTGCCTCAAGCTGAAGGAGGT 900
CN34_VP2        ACAAATAGAGCATTGGGCTTACCACCATTTCTAAATTCTTTGCCTCAAGCTGAAGGAGGT 900
CN35_VP2        ACAAATAGAGCATTGGGCTTACCACCATTTCTAAATTCTTTGCCTCAAGCTGAAGGAGGT 900
CN36_VP2        ACAAATAGAGCATTGGGCTTACCACCATTTCTAAATTCTTTGCCTCAAGCTGAAGGAGGT 900
CN37_VP2        ACAAATAGAGCATTGGGCTTACCACCATTTCTAAATTCTTTGCCTCAAGCTGAAGGAGGT 900
CN38_VP2        ACAAATAGAGCATTGGGCTTACCACCATTTCTAAATTCTTTGCCTCAAGCTGAAGGAGGT 900
CN39_VP2        ACAAATAGAGCATTGGGCTTACCACCATTTCTAAATTCTTTGCCTCAAGCTGAAGGAGGT 900
CN40_VP2        ACAAATAGAGCATTGGGCTTACCACCATTTCTAAATTCTTTGCCTCAAGCTGAAGGAGGT 900
CK81_VP2        ACAAATAGAGCATTGGGCTTACCACCATTTCTAAATTCTTTGCCTCAAGCTGAAGGAGGT 900
CK84_VP2        ACAAATAGAGCATTGGGCTTACCACCATTTCTAAATTCTTTGCCTCAAGCTGAAGGAGGT 900
RVC50_VP2       ACAAATAGAGCATTGGGCTTACCACCATTTCTAAATTCTTTGCCTCAAGCTGAAGGAGGT 900
                ************************************************************

CN7_VP2         ACTAACTTTGGTTATATAGGAGTTCAACAAGATAAAAGACGTGGTGTAACTCAAATGGGA 960
CN8_VP2         ACTAACTTTGGTTATATAGGAGTTCAACAAGATAAAAGACGTGGTGTAACTCAAATGGGA 960
CN10_VP2        ACTAACTTTGGTTATATAGGAGTTCAACAAGATAAAAGACGTGGTGTAACTCAAATGGGA 960
CN11_VP2        ACTAACTTTGGTTATATAGGAGTTCAACAAGATAAAAGACGTGGTGTAACTCAAATGGGA 960
CN12_VP2        ACTAACTTTGGTTATATAGGAGTTCAACAAGATAAAAGACGTGGTGTAACTCAAATGGGA 960
CN13_VP2        ACTAACTTTGGTTATATAGGAGTTCAACAAGATAAAAGACGTGGTGTAACTCAAATGGGA 960
CN14_VP2        ACTAACTTTGGTTATATAGGAGTTCAACAAGATAAAAGACGTGGTGTAACTCAAATGGGA 960
CN15_VP2        ACTAACTTTGGTTATATAGGAGTTCAACAAGATAAAAGACGTGGTGTAACTCAAATGGGA 960
CN16_VP2        ACTAACTTTGGTTATATAGGAGTTCAACAAGATAAAAGACGTGGTGTAACTCAAATGGGA 960
CN17_VP2        ACTAACTTTGGTTATATAGGAGTTCAACAAGATAAAAGACGTGGTGTAACTCAAATGGGA 960
CN18_VP2        ACTAACTTTGGTTATATAGGAGTTCAACAAGATAAAAGACGTGGTGTAACTCAAATGGGA 960
CN19_VP2        ACTAACTTTGGTTATATAGGAGTTCAACAAGATAAAAGACGTGGTGTAACTCAAATGGGA 960
CN20_VP2        ACTAACTTTGGTTATATAGGAGTTCAACAAGATAAAAGACGTGGTGTAACTCAAATGGGA 960
CN21_VP2        ACTAACTTTGGTTATATAGGAGTTCAACAAGATAAAAGACGTGGTGTAACTCAAATGGGA 960
CN22_VP2        ACTAACTTTGGTTATATAGGAGTTCAACAAGATAAAAGACGTGGTGTAACTCAAATGGGA 960
CN23_VP2        ACTAACTTTGGTTATATAGGAGTTCAACAAGATAAAAGACGTGGTGTAACTCAAATGGGA 960
CN24_VP2        ACTAACTTTGGTTATATAGGAGTTCAACAAGATAAAAGACGTGGTGTAACTCAAATGGGA 960
CN25_VP2        ACTAACTTTGGTTATATAGGAGTTCAACAAGATAAAAGACGTGGTGTAACTCAAATGGGA 960
CN26_VP2        ACTAACTTTGGTTATATAGGAGTTCAACAAGATAAAAGACGTGGTGTAACTCAAATGGGA 960
CN27_VP2        ACTAACTTTGGTTATATAGGAGTTCAACAAGATAAAAGACGTGGTGTAACTCAAATGGGA 960
CN28_VP2        ACTAACTTTGGTTATATAGGAGTTCAACAAGATAAAAGACGTGGTGTAACTCAAATGGGA 960
CN29_VP2        ACTAACTTTGGTTATATAGGAGTTCAACAAGATAAAAGACGTGGTGTAACTCAAATGGGA 960
CN30_VP2        ACTAACTTTGGTTATATAGGAGTTCAACAAGATAAAAGACGTGGTGTAACTCAAATGGGA 960
CN31_VP2        ACTAACTTTGGTTATATAGGAGTTCAACAAGATAAAAGACGTGGTGTAACTCAAATGGGA 960
CN32_VP2        ACTAACTTTGGTTATATAGGAGTTCAACAAGATAAAAGACGTGGTGTAACTCAAATGGGA 960
CN33_VP2        ACTAACTTTGGTTATATAGGAGTTCAACAAGATAAAAGACGTGGTGTAACTCAAATGGGA 960
CN34_VP2        ACTAACTTTGGTTATATAGGAGTTCAACAAGATAAAAGACGTGGTGTAACTCAAATGGGA 960
CN35_VP2        ACTAACTTTGGTTATATAGGAGTTCAACAAGATAAAAGACGTGGTGTAACTCAAATGGGA 960
CN36_VP2        ACTAACTTTGGTTATATAGGAGTTCAACAAGATAAAAGACGTGGTGTAACTCAAATGGGA 960
CN37_VP2        ACTAACTTTGGTTATATAGGAGTTCAACAAGATAAAAGACGTGGTGTAACTCAAATGGGA 960
CN38_VP2        ACTAACTTTGGTTATATAGGAGTTCAACAAGATAAAAGACGTGGTGTAACTCAAATGGGA 960
CN39_VP2        ACTAACTTTGGTTATATAGGAGTTCAACAAGATAAAAGACGTGGTGTAACTCAAATGGGA 960
CN40_VP2        ACTAACTTTGGTTATATAGGAGTTCAACAAGATAAAAGACGTGGTGTAACTCAAATGGGA 960
CK81_VP2        ACTAACTTTGGTTATATAGGAGTTCAACAAGATAAAAGACGTGGTGTAACTCAAATGGGA 960
CK84_VP2        ACTAACTTTGGTTATATAGGAGTTCAACAAGATAAAAGACGTGGTGTAACTCAAATGGGA 960
RVC50_VP2       ACTAACTTTGGTTATATAGGAGTTCAACAAGATAAAAGACGTGGTGTAACTCAAATGGGA 960
                ************************************************************
```

```
CN7_VP2         AATACAAACTATATTACTGAAGCTACTATTATGAGACCAGCTGAGGTTGGTTATAGTGCA 1020
CN8_VP2         AATACAAACTATATTACTGAAGCTACTATTATGAGACCAGCTGAGGTTGGTTATAGTGCA 1020
CN10_VP2        AATACAAACTATATTACTGAAGCTACTATTATGAGACCAGCTGAGGTTGGTTATAGTGCA 1020
CN11_VP2        AATACAAACTATATTACTGAAGCTACTATTATGAGACCAGCTGAGGTTGGTTATAGTGCA 1020
CN12_VP2        AATACAAACTATATTACTGAAGCTACTATTATGAGACCAGCTGAGGTTGGTTATAGTGCA 1020
CN13_VP2        AATACAAACTATATTACTGAAGCTACTATTATGAGACCAGCTGAGGTTGGTTATAGTGCA 1020
CN14_VP2        AATACAAACTATATTACTGAAGCTACTATTATGAGACCAGCTGAGGTTGGTTATAGTGCA 1020
CN15_VP2        AATACAAACTATATTACTGAAGCTACTATTATGAGACCAGCTGAGGTTGGTTATAGTGCA 1020
CN16_VP2        AATACAAACTATATTACTGAAGCTACTATTATGAGACCAGCTGAGGTTGGTTATAGTGCA 1020
CN17_VP2        AATACAAACTATATTACTGAAGCTACTATTATGAGACCAGCTGAGGTTGGTTATAGTGCA 1020
CN18_VP2        AATACAAACTATATTACTGAAGCTACTATTATGAGACCAGCTGAGGTTGGTTATAGTGCA 1020
CN19_VP2        AATACAAACTATATTACTGAAGCTACTATTATGAGACCAGCTGAGGTTGGTTATAGTGCA 1020
CN20_VP2        AATACAAACTATATTACTGAAGCTACTATTATGAGACCAGCTGAGGTTGGTTATAGTGCA 1020
CN21_VP2        AATACAAACTATATTACTGAAGCTACTATTATGAGACCAGCTGAGGTTGGTTATAGTGCA 1020
CN22_VP2        AATACAAACTATATTACTGAAGCTACTATTATGAGACCAGCTGAGGTTGGTTATAGTGCA 1020
CN23_VP2        AATACAAACTATATTACTGAAGCTACTATTATGAGACCAGCTGAGGTTGGTTATAGTGCA 1020
CN24_VP2        AATACAAACTATATTACTGAAGCTACTATTATGAGACCAGCTGAGGTTGGTTATAGTGCA 1020
CN25_VP2        AATACAAACTATATTACTGAAGCTACTATTATGAGACCAGCTGAGGTTGGTTATAGTGCA 1020
CN26_VP2        AATACAAACTATATTACTGAAGCTACTATTATGAGACCAGCTGAGGTTGGTTATAGTGCA 1020
CN27_VP2        AATACAAACTATATTACTGAAGCTACTATTATGAGACCAGCTGAGGTTGGTTATAGTGCA 1020
CN28_VP2        AATACAAACTATATTACTGAAGCTACTATTATGAGACCAGCTGAGGTTGGTTATAGTGCA 1020
CN29_VP2        AATACAAACTATATTACTGAAGCTACTATTATGAGACCAGCTGAGGTTGGTTATAGTGCA 1020
CN30_VP2        AATACAAACTATATTACTGAAGCTACTATTATGAGACCAGCTGAGGTTGGTTATAGTGCA 1020
CN31_VP2        AATACAAACTATATTACTGAAGCTACTATTATGAGACCAGCTGAGGTTGGTTATAGTGCA 1020
CN32_VP2        AATACAAACTATATTACTGAAGCTACTATTATGAGACCAGCTGAGGTTGGTTATAGTGCA 1020
CN33_VP2        AATACAAACTATATTACTGAAGCTACTATTATGAGACCAGCTGAGGTTGGTTATAGTGCA 1020
CN34_VP2        AATACAAACTATATTACTGAAGCTACTATTATGAGACCAGCTGAGGTTGGTTATAGTGCA 1020
CN35_VP2        AATACAAACTATATTACTGAAGCTACTATTATGAGACCAGCTGAGGTTGGTTATAGTGCA 1020
CN36_VP2        AATACAAACTATATTACTGAAGCTACTATTATGAGACCAGCTGAGGTTGGTTATAGTGCA 1020
CN37_VP2        AATACAAACTATATTACTGAAGCTACTATTATGAGACCAGCTGAGGTTGGTTATAGTGCA 1020
CN38_VP2        AATACAAACTATATTACTGAAGCTACTATTATGAGACCAGCTGAGGTTGGTTATAGTGCA 1020
CN39_VP2        AATACAAACTATATTACTGAAGCTACTATTATGAGACCAGCTGAGGTTGGTTATAGTGCA 1020
CN40_VP2        AATACAAACTATATTACTGAAGCTACTATTATGAGACCAGCTGAGGTTGGTTATAGTGCA 1020
CK81_VP2        AATACAAACTATATTACTGAAGCTACTATTATGAGACCAGCTGAGGTTGGTTATAGTGCA 1020
CK84_VP2        AATACAAACTATATTACTGAAGCTACTATTATGAGACCAGCTGAGGTTGGTTATAGTGCA 1020
RVC50_VP2       AATACAAACTATATTACTGAAGCTACTATTATGAGACCAGCTGAGGTTGGTTATAGTGCA 1020
                ************************************************************
```

```
CN7_VP2         CCATATTATTCTTTTGAGGCGTCTACACAAGGGCCATTTAAAACACCCATTGCAGCAGGA 1080
CN8_VP2         CCATATTATTCTTTTGAGGCGTCTACACAAGGGCCATTTAAAACACCCATTGCAGCAGGA 1080
CN10_VP2        CCATATTATTCTTTTGAGGCGTCTACACAAGGGCCATTTAAAACACCCATTGCAGCAGGA 1080
CN11_VP2        CCATATTATTCTTTTGAGGCGTCTACACAAGGGCCATTTAAAACACCCATTGCAGCAGGA 1080
CN12_VP2        CCATATTATTCTTTTGAGGCGTCTACACAAGGGCCATTTAAAACACCCATTGCAGCAGGA 1080
CN13_VP2        CCATATTATTCTTTTGAGGCGTCTACACAAGGGCCATTTAAAACACCCATTGCAGCAGGA 1080
CN14_VP2        CCATATTATTCTTTTGAGGCGTCTACACAAGGGCCATTTAAAACACCCATTGCAGCAGGA 1080
CN15_VP2        CCATATTATTCTTTTGAGGCGTCTACACAAGGGCCATTTAAAACACCCATTGCAGCAGGA 1080
CN16_VP2        CCATATTATTCTTTTGAGGCGTCTACACAAGGGCCATTTAAAACACCCATTGCAGCAGGA 1080
CN17_VP2        CCATATTATTCTTTTGAGGCGTCTACACAAGGGCCATTTAAAACACCCATTGCAGCAGGA 1080
CN18_VP2        CCATATTATTCTTTTGAGGCGTCTACACAAGGGCCATTTAAAACACCCATTGCAGCAGGA 1080
CN19_VP2        CCATATTATTCTTTTGAGGCGTCTACACAAGGGCCATTTAAAACACCCATTGCAGCAGGA 1080
CN20_VP2        CCATATTATTCTTTTGAGGCGTCTACACAAGGGCCATTTAAAACACCCATTGCAGCAGGA 1080
CN21_VP2        CCATATTATTCTTTTGAGGCGTCTACACAAGGGCCATTTAAAACACCCATTGCAGCAGGA 1080
CN22_VP2        CCATATTATTCTTTTGAGGCGTCTACACAAGGGCCATTTAAAACACCCATTGCAGCAGGA 1080
CN23_VP2        CCATATTATTCTTTTGAGGCGTCTACACAAGGGCCATTTAAAACACCCATTGCAGCAGGA 1080
CN24_VP2        CCATATTATTCTTTTGAGGCGTCTACACAAGGGCCATTTAAAACACCCATTGCAGCAGGA 1080
CN25_VP2        CCATATTATTCTTTTGAGGCGTCTACACAAGGGCCATTTAAAACACCCATTGCAGCAGGA 1080
CN26_VP2        CCATATTATTCTTTTGAGGCGTCTACACAAGGGCCATTTAAAACACCCATTGCAGCAGGA 1080
CN27_VP2        CCATATTATTCTTTTGAGGCGTCTACACAAGGGCCATTTAAAACACCCATTGCAGCAGGA 1080
CN28_VP2        CCATATTATTCTTTTGAGGCGTCTACACAAGGGCCATTTAAAACACCCATTGCAGCAGGA 1080
CN29_VP2        CCATATTATTCTTTTGAGGCGTCTACACAAGGGCCATTTAAAACACCCATTGCAGCAGGA 1080
CN30_VP2        CCATATTATTCTTTTGAGGCGTCTACACAAGGGCCATTTAAAACACCCATTGCAGCAGGA 1080
CN31_VP2        CCATATTATTCTTTTGAGGCGTCTACACAAGGGCCATTTAAAACACCCATTGCAGCAGGA 1080
CN32_VP2        CCATATTATTCTTTTGAGGCGTCTACACAAGGGCCATTTAAAACACCCATTGCAGCAGGA 1080
CN33_VP2        CCATATTATTCTTTTGAGGCGTCTACACAAGGGCCATTTAAAACACCCATTGCAGCAGGA 1080
CN34_VP2        CCATATTATTCTTTTGAGGCGTCTACACAAGGGCCATTTAAAACACCCATTGCAGCAGGA 1080
CN35_VP2        CCATATTATTCTTTTGAGGCGTCTACACAAGGGCCATTTAAAACACCCATTGCAGCAGGA 1080
CN36_VP2        CCATATTATTCTTTTGAGGCGTCTACACAAGGGCCATTTAAAACACCCATTGCAGCAGGA 1080
CN37_VP2        CCATATTATTCTTTTGAGGCGTCTACACAAGGGCCATTTAAAACACCCATTGCAGCAGGA 1080
CN38_VP2        CCATATTATTCTTTTGAGGCGTCTACACAAGGGCCATTTAAAACACCCATTGCAGCAGGA 1080
CN39_VP2        CCATATTATTCTTTTGAGGCGTCTACACAAGGGCCATTTAAAACACCCATTGCAGCAGGA 1080
CN40_VP2        CCATATTATTCTTTTGAGGCGTCTACACAAGGGCCATTTAAAACACCCATTGCAGCAGGA 1080
CK81_VP2        CCATATTATTCTTTTGAGGCGTCTACACAAGGGCCATTTAAAACACCCATTGCAGCAGGA 1080
CK84_VP2        CCATATTATTCTTTTGAGGCGTCTACACAAGGGCCATTTAAAACACCCATTGCAGCAGGA 1080
RVC50_VP2       CCATATTATTCTTTTGAGGCGTCTACACAAGGGCCATTTAAAACACCCATTGCAGCAGGA 1080
                ************************************************************
```

```
CN7_VP2         CGGGGGGGAGCGCAAACAGATGAAAATCAAGCAGCAGATGGTGATCCAAGATATGCATTT 1140
CN8_VP2         CGGGGGGGAGCGCAAACAGATGAAAATCAAGCAGCAGATGGTGATCCAAGATATGCATTT 1140
CN10_VP2        CGGGGGGGAGCGCAAACAGATGAAAATCAAGCAGCAGATGGTGATCCAAGATATGCATTT 1140
CN11_VP2        CGGGGGGGAGCGCAAACAGATGAAAATCAAGCAGCAGATGGTGATCCAAGATATGCATTT 1140
CN12_VP2        CGGGGGGGAGCGCAAACAGATGAAAATCAAGCAGCAGATGGTGATCCAAGATATGCATTT 1140
CN13_VP2        CGGGGGGGAGCGCAAACAGATGAAAATCAAGCAGCAGATGGTGATCCAAGATATGCATTT 1140
CN14_VP2        CGGGGGGGAGCGCAAACAGATGAAAATCAAGCAGCAAATGGTGATCCAAGATATGCATTT 1140
CN15_VP2        CGGGGGGGAGCGCAAACAGATGAAAATCAAGCAGCAGATGGTGATCCAAGATATGCATTT 1140
CN16_VP2        CGGGGGGGAGCGCAAACAGATGAAAATCAAGCAGCAGATGGTGATCCAAGATATGCATTT 1140
CN17_VP2        CGGGGGGGAGCGCAAACAGATGAAAATCAAGCAGCAGATGGTGATCCAAGATATGCATTT 1140
CN18_VP2        CGGGGGGGAGCGCAAACAGATGAAAATCAAGCAGCAGATGGTGATCCAAGATATGCATTT 1140
CN19_VP2        CGGGGGGGAGCGCAAACAGATGAAAATCAAGCAGCAGATGGTGATCCAAGATATGCATTT 1140
CN20_VP2        CGGGGGGGAGCGCAAACAGATGAAAATCAAGCAGCAGATGGTGATCCAAGATATGCATTT 1140
CN21_VP2        CGGGGGGGAGCGCAAACAGATGAAAATCAAGCAGCAGATGGTGATCCAAGATATGCATTT 1140
CN22_VP2        CGGGGGGGAGCGCAAACAGATGAAAATCAAGCAGCAGATGGTGATCCAAGATATGCATTT 1140
CN23_VP2        CGGGGGGGAGCGCAAACAGATGAAAATCAAGCAGCAAATGGTGATCCAAGATATGCATTT 1140
CN24_VP2        CGGGGGGGAGCGCAAACAGATGAAAATCAAGCAGCAGATGGTGATCCAAGATATGCATTT 1140
CN25_VP2        CGGGGGGGAGCGCAAACAGATGAAAATCAAGCAGCAAATGGTGATCCAAGATATGCATTT 1140
CN26_VP2        CGGGGGGGAGCGCAAACAGATGAAAATCAAGCAGCAGATGGTGATCCAAGATATGCATTT 1140
CN27_VP2        CGGGGGGGAGCGCAAACAGATGAAAATCAAGCAGCAGATGGTGATCCAAGATATGCATTT 1140
CN28_VP2        CGGGGGGGAGCGCAAACAGATGAAAATCAAGCAGCAGATGGTGATCCAAGATATGCATTT 1140
CN29_VP2        CGGGGGGGAGCGCAAACAGATGAAAATCAAGCAGCAGATGGTGATCCAAGATATGCATTT 1140
CN30_VP2        CGGGGGGGAGCGCAAACAGATGAAAATCAAGCAGCAGATGGTGATCCAAGATATGCATTT 1140
CN31_VP2        CGGGGGGGAGCGCAAACAGATGAAAATCAAGCAGCAGATGGTGATCCAAGATATGCATTT 1140
CN32_VP2        CGGGGGGGAGCGCAAACAGATGAAAATCAAGCAGCAGATGGTGATCCAAGATATGCATTT 1140
CN33_VP2        CGGGGGGGAGCGCAAACAGATGAAAATCAAGCAGCAGATGGTGATCCAAGATATGCATTT 1140
CN34_VP2        CGGGGGGGAGCGCAAACAGATGAAAATCAAGCAGCAGATGGTGATCCAAGATATGCATTT 1140
CN35_VP2        CGGGGGGGAGCGCAAACAGATGAAAATCAAGCAGCAGATGGTGATCCAAGATATGCATTT 1140
CN36_VP2        CGGGGGGGAGCGCAAACAGATGAAAATCAAGCAGCAGATGGTGATCCAAGATATGCATTT 1140
CN37_VP2        CGGGGGGGAGCGCAAACAGATGAAAATCAAGCAGCAGATGGTGATCCAAGATATGCATTT 1140
CN38_VP2        CGGGGGGGAGCGCAAACAGATGAAAATCAAGCAGCAGATGGTGATCCAAGATATGCATTT 1140
CN39_VP2        CGGGGGGGAGCGCAAACAGATGAAAATCAAGCAGCAGATGGTGATCCAAGATATGCATTT 1140
CN40_VP2        CGGGGGGGAGCGCAAACAGATGAAAATCAAGCAGCAGATGGTGATCCAAGATATGCATTT 1140
CK81_VP2        CGGGGGGGAGCGCAAACAGATGAAAATCAAGCAGCAGATGGTGATCCAAGATATGCATTT 1140
CK84_VP2        CGGGGGGGAGCGCAAACAGATGAAAATCAAGCAGCAGATGGTGATCCAAGATATGCATTT 1140
RVC50_VP2       CGGGGGGGAGCGCAAACAGATGAAAATCAAGCAGCAGATGGTGATCCAAGATATGCATTT 1140
                ************************************ ***********************

CN7_VP2         GGTAGACAACATGGTCAAAAAACTACCACAACAGGAGAAACACCTGAGAGATTTACATAT 1200
CN8_VP2         GGTAGACAACATGGTCAAAAAACTACCACAACAGGAGAAACACCTGAGAGATTTACATAT 1200
CN10_VP2        GGTAGACAACATGGTCAAAAAACTACCACAACAGGAGAAACACCTGAGAGATTTACATAT 1200
CN11_VP2        GGTAGACAACATGGTCAAAAAACTACCACAACAGGAGAAACACCTGAGAGATTTACATAT 1200
CN12_VP2        GGTAGACAACATGGTCAAAAAACTACCACAACAGGAGAAACACCTGAGAGATTTACATAT 1200
CN13_VP2        GGTAGACAACATGGTCAAAAAACTACCACAACAGGAGAAACACCTGAGAGATTTACATAT 1200
CN14_VP2        GGTAGACAACATGGTCAAAAAACTACCACAACAGGAGAAACACCTGAAAGATTTACATAT 1200
CN15_VP2        GGTAGACAACATGGTCAAAAAACTACCACAACAGGAGAAACACCTGAGAGATTTACATAT 1200
CN16_VP2        GGTAGACAACATGGTCAAAAAACTACCACAACAGGAGAAACACCTGAGAGATTTACATAT 1200
CN17_VP2        GGTAGACAACATGGTCAAAAAACTACCACAACAGGAGAAACACCTGAGAGATTTACATAT 1200
CN18_VP2        GGTAGACAACATGGTCAAAAAACTACCACAACAGGAGAAACACCTGAGAGATTTACATAT 1200
CN19_VP2        GGTAGACAACATGGTCAAAAAACTACCACAACAGGAGAAACACCTGAGAGATTTACATAT 1200
CN20_VP2        GGTAGACAACATGGTCAAAAAACTACCACAACAGGAGAAACACCTGAGAGATTTACATAT 1200
CN21_VP2        GGTAGACAACATGGTCAAAAAACTACCACAACAGGAGAAACACCTGAGAGATTTACATAT 1200
CN22_VP2        GGTAGACAACATGGTCAAAAAACTACCACAACAGGAGAAACACCTGAGAGATTTACATAT 1200
CN23_VP2        GGTAGACAACATGGTCAAAAAACTACCACAACAGGAGAAACACCTGAGAGATTTACATAT 1200
CN24_VP2        GGTAGACAACATGGTCAAAAAACTACCACAACAGGAGAAACACCTGAGAGATTTACATAT 1200
CN25_VP2        GGTAGACAACATGGTCAAAAAACTACCACAACAGGAGAAACACCTGAGAGATTTACATAT 1200
CN26_VP2        GGTAGACAACATGGTCAAAAAACTACCACAACAGGAGAAACACCTGAGAGATTTACATAT 1200
CN27_VP2        GGTAGACAACATGGTCAAAAAACTACCACAACAGGAGAAACACCTGAGAGATTTACATAT 1200
CN28_VP2        GGTAGACAACATGGTCAAAAAACTACCACAACAGGAGAAACACCTGAGAGATTTACATAT 1200
CN29_VP2        GGTAGACAACATGGTCAAAAAACTACCACAACAGGAGAAACACCTGAGAGATTTACATAT 1200
CN30_VP2        GGTAGACAACATGGTCAAAAAACTACCACAACAGGAGAAACACCTGAGAGATTTACATAT 1200
CN31_VP2        GGTAGACAACATGGTCAAAAAACTACCACAACAGGAGAAACACCTGAGAGATTTACATAT 1200
CN32_VP2        GGTAGACAACATGGTCAAAAAACTACCACAACAGGAGAAACACCTGAGAGATTTACATAT 1200
CN33_VP2        GGTAGACAACATGGTCAAAAAACTACCACAACAGGAGAAACACCTGAGAGATTTACATAT 1200
CN34_VP2        GGTAGACAACATGGTCAAAAAACTACCACAACAGGAGAAACACCTGAGAGATTTACATAT 1200
CN35_VP2        GGTAGACAACATGGTCAAAAAACTACCACAACAGGAGAAACACCTGAGAGATTTACATAT 1200
CN36_VP2        GGTAGACAACATGGTCAAAAAACTACCACAACAGGAGAAACACCTGAGAGATTTACATAT 1200
CN37_VP2        GGTAGACAACATGGTCAAAAAACTACCACAACAGGAGAAACACCTGAGAGATTTACATAT 1200
CN38_VP2        GGTAGACAACATGGTCAAAAAACTACCACAACAGGAGAAACACCTGAGAGATTTACATAT 1200
CN39_VP2        GGTAGACAACATGGTCAAAAAACTACCACAACAGGAGAAACACCTGAGAGATTTACATAT 1200
CN40_VP2        GGTAGACAACATGGTCAAAAAACTACCACAACAGGAGAAACACCTGAGAGATTTACATAT 1200
CK81_VP2        GGTAGACAACATGGTCAAAAAACTACCACAACAGGAGAAACACCTGAGAGATTTACATAT 1200
CK84_VP2        GGTAGACAACATGGTCAAAAAACTACCACAACAGGAGAAACACCTGAGAGATTTACATAT 1200
RVC50_VP2       GGTAGACAACATGGTCAAAAAACTACCACAACAGGAGAAACACCTGAGAGATTTACATAT 1200
                *********************************************** ************
```

```
CN7_VP2         ATAGCACATCAAGATACAGGAAGATATCCAGAAGGAGATTGGATTCAAAATATTAACTTT 1260
CN8_VP2         ATAGCACATCAAGATACAGGAAGATATCCAGAAGGAGATTGGATTCAAAATATTAACTTT 1260
CN10_VP2        ATAGCACATCAAGATACAGGAAGATATCCAGAAGGAGATTGGATTCAAAATATTAACTTT 1260
CN11_VP2        ATAGCACATCAAGATACAGGAAGATATCCAGAAGGAGATTGGATTCAAAATATTAACTTT 1260
CN12_VP2        ATAGCACATCAAGATACAGGAAGATATCCAGAAGGAGATTGGATTCAAAATATTAACTTT 1260
CN13_VP2        ATAGCACATCAAGATACAGGAAGATATCCAGAAGGAGATTGGATTCAAAATATTAACTTT 1260
CN14_VP2        ATAGCACATCAAGATACAGGAAGATATCCAGAAGGAGATTGGATTCAAAATATTAACTTT 1260
CN15_VP2        ATAGCACATCAAGATACAGGAAGATATCCAGAAGGAGATTGGATTCAAAATATTAACTTT 1260
CN16_VP2        ATAGCACATCAAGATACAGGAAGATATCCAGAAGGAGATTGGATTCAAAATATTAACTTT 1260
CN17_VP2        ATAGCACATCAAGATACAGGAAGATATCCAGAAGGAGATTGGATTCAAAATATTAACTTT 1260
CN18_VP2        ATAGCACATCAAGATACAGGAAGATATCCAGAAGGAGATTGGATTCAAAATATTAACTTT 1260
CN19_VP2        ATAGCACATCAAGATACAGGAAGATATCCAGAAGGAGATTGGATTCAAAATATTAACTTT 1260
CN20_VP2        ATAGCACATCAAGATACAGGAAGATATCCAGAAGGAGATTGGATTCAAAATATTAACTTT 1260
CN21_VP2        ATAGCACATCAAGATACAGGAAGATATCCAGAAGGAGATTGGATTCAAAATATTAACTTT 1260
CN22_VP2        ATAGCACATCAAGATACAGGAAGATATCCAGAAGGAGATTGGATTCAAAATATTAACTTT 1260
CN23_VP2        ATAGCACATCAAGATACAGGAAGATATCCAGAAGGAGATTGGATTCAAAATATTAACTTT 1260
CN24_VP2        ATAGCACATCAAGATACAGGAAGATATCCAGAAGGAGATTGGATTCAAAATATTAACTTT 1260
CN25_VP2        ATAGCACATCAAGATACAGGAAGATATCCAGAAGGAGATTGGATTCAAAATATTAACTTT 1260
CN26_VP2        ATAGCACATCAAGATACAGGAAGATATCCAGAAGGAGATTGGATTCAAAATATTAACTTT 1260
CN27_VP2        ATAGCACATCAAGATACAGGAAGATATCCAGAAGGAGATTGGATTCAAAATATTAACTTT 1260
CN28_VP2        ATAGCACATCAAGATACAGGAAGATATCCAGAAGGAGATTGGATTCAAAATATTAACTTT 1260
CN29_VP2        ATAGCACATCAAGATACAGGAAGATATCCAGAAGGAGATTGGATTCAAAATATTAACTTT 1260
CN30_VP2        ATAGCACATCAAGATACAGGAAGATATCCAGAAGGAGATTGGATTCAAAATATTAACTTT 1260
CN31_VP2        ATAGCACATCAAGATACAGGAAGATATCCAGAAGGAGATTGGATTCAAAATATTAACTTT 1260
CN32_VP2        ATAGCACATCAAGATACAGGAAGATATCCAGAAGGAGATTGGATTCAAAATATTAACTTT 1260
CN33_VP2        ATAGCACATCAAGATACAGGAAGATATCCAGAAGGAGATTGGATTCAAAATATTAACTTT 1260
CN34_VP2        ATAGCACATCAAGATACAGGAAGATATCCAGAAGGAGATTGGATTCAAAATATTAACTTT 1260
CN35_VP2        ATAGCACATCAAGATACAGGAAGATATCCAGAAGGAGATTGGATTCAAAATATTAACTTT 1260
CN36_VP2        ATAGCACATCAAGATACAGGAAGATATCCAGAAGGAGATTGGATTCAAAATATTAACTTT 1260
CN37_VP2        ATAGCACATCAAGATACAGGAAGATATCCAGAAGGAGATTGGATTCAAAATATTAACTTT 1260
CN38_VP2        ATAGCACATCAAGATACAGGAAGATATCCAGAAGGAGATTGGATTCAAAATATTAACTTT 1260
CN39_VP2        ATAGCACATCAAGATACAGGAAGATATCCAGAAGGAGATTGGATTCAAAATATTAACTTT 1260
CN40_VP2        ATAGCACATCAAGATACAGGAAGATATCCAGAAGGAGATTGGATTCAAAATATTAACTTT 1260
CK81_VP2        ATAGCACATCAAGATACAGGAAGATATCCAGAAGGAGATTGGATTCAAAATATTAACTTT 1260
CK84_VP2        ATAGCACATCAAGATACAGGAAGATATCCAGAAGGAGATTGGATTCAAAATATTAACTTT 1260
RVC50_VP2       ATAGCACATCAAGATACAGGAAGATATCCAGAAGGAGATTGGATTCAAAATATTAACTTT 1260
                ************************************************************

CN7_VP2         AACCTTCCTGTAACAAATGATAATGTATTGCTACCAACAGATCCAATTGGAGGTAAAACA 1320
CN8_VP2         AACCTTCCTGTAACAAATGATAATGTATTGCTACCAACAGATCCAATTGGAGGTAAAACA 1320
CN10_VP2        AACCTTCCTGTAACAAATGATAATGTATTGCTACCAACAGATCCAATTGGAGGTAAAACA 1320
CN11_VP2        AACCTTCCTGTAACAAATGATAATGTATTGCTACCAACAGATCCAATTGGAGGTAAAACA 1320
CN12_VP2        AACCTTCCTGTAACAAATGATAATGTATTGCTACCAACAGATCCAATTGGAGGTAAAACA 1320
CN13_VP2        AACCTTCCTGTAACAAATGATAATGTATTGCTACCAACAGATCCAATTGGAGGTAAAACA 1320
CN14_VP2        AACCTTCCTGTAACAAATGATAATGTATTGCTACCAACAGATCCAATTGGAGGTAAAACA 1320
CN15_VP2        AACCTTCCTGTAACAAATGATAATGTATTGCTACCAACAGATCCAATTGGAGGTAAAACA 1320
CN16_VP2        AACCTTCCTGTAACAAATGATAATGTATTGCTACCAACAGATCCAATTGGAGGTAAAACA 1320
CN17_VP2        AACCTTCCTGTAACAAATGATAATGTATTGCTACCAACAGATCCAATTGGAGGTAAAACA 1320
CN18_VP2        AACCTTCCTGTAACAAATGATAATGTATTGCTACCAACAGATCCAATTGGAGGTAAAACA 1320
CN19_VP2        AACCTTCCTGTAACAAATGATAATGTATTGCTACCAACAGATCCAATTGGAGGTAAAACA 1320
CN20_VP2        AACCTTCCTGTAACAAATGATAATGTATTGCTACCAACAGATCCAATTGGAGGTAAAACA 1320
CN21_VP2        AACCTTCCTGTAACAAATGATAATGTATTGCTACCAACAGATCCAATTGGAGGTAAAACA 1320
CN22_VP2        AACCTTCCTGTAACAAATGATAATGTATTGCTACCAACAGATCCAATTGGAGGTAAAACA 1320
CN23_VP2        AACCTTCCTGTAACAAATGATAATGTATTGCTACCAACAGATCCAATTGGAGGTAAAACA 1320
CN24_VP2        AACCTTCCTGTAACAAATGATAATGTATTGCTACCAACAGATCCAATTGGAGGTAAAACA 1320
CN25_VP2        AACCTTCCTGTAACAAATGATAATGTATTGCTACCAACAGATCCAATTGGAGGTAAAACA 1320
CN26_VP2        AACCTTCCTGTAACAAATGATAATGTATTGCTACCAACAGATCCAATTGGAGGTAAAACA 1320
CN27_VP2        AACCTTCCTGTAACAAATGATAATGTATTGCTACCAACAGATCCAATTGGAGGTAAAACA 1320
CN28_VP2        AACCTTCCTGTAACAAATGATAATGTATTGCTACCAACAGATCCAATTGGAGGTAAAACA 1320
CN29_VP2        AACCTTCCTGTAACAAATGATAATGTATTGCTACCAACAGATCCAATTGGAGGTAAAACA 1320
CN30_VP2        AACCTTCCTGTAACAAATGATAATGTATTGCTACCAACAGATCCAATTGGAGGTAAAACA 1320
CN31_VP2        AACCTTCCTGTAACAAATGATAATGTATTGCTACCAACAGATCCAATTGGAGGTAAAACA 1320
CN32_VP2        AACCTTCCTGTAACAAATGATAATGTATTGCTACCAACAGATCCAATTGGAGGTAAAACA 1320
CN33_VP2        AACCTTCCTGTAACAAATGATAATGTATTGCTACCAACAGATCCAATTGGAGGTAAAACA 1320
CN34_VP2        AACCTTCCTGTAACAAATGATAATGTATTGCTACCAACAGATCCAATTGGAGGTAAAACA 1320
CN35_VP2        AACCTTCCTGTAACAAATGATAATGTATTGCTACCAACAGATCCAATTGGAGGTAAAACA 1320
CN36_VP2        AACCTTCCTGTAACAAATGATAATGTATTGCTACCAACAGATCCAATTGGAGGTAAAACA 1320
CN37_VP2        AACCTTCCTGTAACAAATGATAATGTATTGCTACCAACAGATCCAATTGGAGGTAAAACA 1320
CN38_VP2        AACCTTCCTGTAACAAATGATAATGTATTGCTACCAACAGATCCAATTGGAGGTAAAACA 1320
CN39_VP2        AACCTTCCTGTAACAAATGATAATGTATTGCTACCAACAGATCCAATTGGAGGTAAAACA 1320
CN40_VP2        AACCTTCCTGTAACAAATGATAATGTATTGCTACCAACAGATCCAATTGGAGGTAAAACA 1320
CK81_VP2        AACCTTCCTGTAACAAATGATAATGTATTGCTACCAACAGATCCAATTGGAGGTAAAACA 1320
CK84_VP2        AACCTTCCTGTAACAAATGATAATGTATTGCTACCAACAGATCCAATTGGAGGTAAAACA 1320
RVC50_VP2       AACCTTCCTGTAACAAATGATAATGTATTGCTACCAACAGATCCAATTGGAGGTAAAACA 1320
                ************************************************************
```

```
CN7_VP2         GGAATTAACTATACTAATATATTTAATACTTATGGTCCTTTAACTGCATTAAATAATGTG 1380
CN8_VP2         GGAATTAACTATACTAATATATTTAATACTTATGGTCCTTTAACTGCATTAAATAATGTG 1380
CN10_VP2        GGAATTAACTATACTAATATATTTAATACTTATGGTCCTTTAACTGCATTAAATAATGTG 1380
CN11_VP2        GGAATTAACTATACTAATATATTTAATACTTATGGTCCTTTAACTGCATTAAATAATGTG 1380
CN12_VP2        GGAATTAACTATACTAATATATTTAATACTTATGGTCCTTTAACTGCATTAAATAATGTG 1380
CN13_VP2        GGAATTAACTATACTAATATATTTAATACTTATGGTCCTTTAACTGCATTAAATAATGTG 1380
CN14_VP2        GGAATTAACTATACTAATATATTTAATACTTATGGTCCTTTAACTGCATTAAATAATGTG 1380
CN15_VP2        GGAATTAACTATACTAATATATTTAATACTTATGGTCCTTTAACTGCATTAAATAATGTG 1380
CN16_VP2        GGAATTAACTATACTAATATATTTAATACTTATGGTCCTTTAACTGCATTAAATAATGTG 1380
CN17_VP2        GGAATTAACTATACTAATATATTTAATACTTATGGTCCTTTAACTGCATTAAATAATGTG 1380
CN18_VP2        GGAATTAACTATACTAATATATTTAATACTTATGGTCCTTTAACTGCATTAAATAATGTG 1380
CN19_VP2        GGAATTAACTATACTAATATATTTAATACTTATGGTCCTTTAACTGCATTAAATAATGTG 1380
CN20_VP2        GGAATTAACTATACTAATATATTTAATACTTATGGTCCTTTAACTGCATTAAATAATGTG 1380
CN21_VP2        GGAATTAACTATACTAATATATTTAATACTTATGGTCCTTTAACTGCATTAAATAATGTG 1380
CN22_VP2        GGAATTAACTATACTAATATATTTAATACTTATGGTCCTTTAACTGCATTAAATAATGTG 1380
CN23_VP2        GGAATTAACTATACTAATATATTTAATACTTATGGTCCTTTAACTGCATTAAATAATGTG 1380
CN24_VP2        GGAATTAACTATACTAATATATTTAATACTTATGGTCCTTTAACTGCATTAAATAATGTG 1380
CN25_VP2        GGAATTAACTATACTAATATATTTAATACTTATGGTCCTTTAACTGCATTAAATAATGTG 1380
CN26_VP2        GGAATTAACTATACTAATATATTTAATACTTATGGTCCTTTAACTGCATTAAATAATGTG 1380
CN27_VP2        GGAATTAACTATACTAATATATTTAATACTTATGGTCCTTTAACTGCATTAAATAATGTG 1380
CN28_VP2        GGAATTAACTATACTAATATATTTAATACTTATGGTCCTTTAACTGCATTAAATAATGTG 1380
CN29_VP2        GGAATTAACTATACTAATATATTTAATACTTATGGTCCTTTAACTGCATTAAATAATGTG 1380
CN30_VP2        GGAATTAACTATACTAATATATTTAATACTTATGGTCCTTTAACTGCATTAAATAATGTG 1380
CN31_VP2        GGAATTAACTATACTAATATATTTAATACTTATGGTCCTTTAACTGCATTAAATAATGTG 1380
CN32_VP2        GGAATTAACTATACTAATATATTTAATACTTATGGTCCTTTAACTGCATTAAATAATGTG 1380
CN33_VP2        GGAATTAACTATACTAATATATTTAATACTTATGGTCCTTTAACTGCATTAAATAATGTG 1380
CN34_VP2        GGAATTAACTATACTAATATATTTAATACTTATGGTCCTTTAACTGCATTAAATAATGTG 1380
CN35_VP2        GGAATTAACTATACTAATATATTTAATACTTATGGTCCTTTAACTGCATTAAATAATGTG 1380
CN36_VP2        GGAATTAACTATACTAATATATTTAATACTTATGGTCCTTTAACTGCATTAAATAATGTG 1380
CN37_VP2        GGAATTAACTATACTAATATATTTAATACTTATGGTCCTTTAACTGCATTAAATAATGTG 1380
CN38_VP2        GGAATTAACTATACTAATATATTTAATACTTATGGTCCTTTAACTGCATTAAATAATGTG 1380
CN39_VP2        GGAATTAACTATACTAATATATTTAATACTTATGGTCCTTTAACTGCATTAAATAATGTG 1380
CN40_VP2        GGAATTAACTATACTAATATATTTAATACTTATGGTCCTTTAACTGCATTAAATAATGTG 1380
CK81_VP2        GGAATTAACTATACTAATATATTTAATACTTATGGTCCTTTAACTGCATTAAATAATGTG 1380
CK84_VP2        GGAATTAACTATACTAATATATTTAATACTTATGGTCCTTTAACTGCATTAAATAATGTG 1380
RVC50_VP2       GGAATTAACTATACTAATATATTTAATACTTATGGTCCTTTAACTGCATTAAATAATGTG 1380
                ************************************************************

CN7_VP2         CCACCAGTTTATCCAAATGGTCAAATTTGGGATAAAGAATTTGATACTGACTTAAAACCA 1440
CN8_VP2         CCACCAGTTTATCCAAATGGTCAAATTTGGGATAAAGAATTTGATACTGACTTAAAACCA 1440
CN10_VP2        CCACCAGTTTATCCAAATGGTCAAATTTGGGATAAAGAATTTGATACTGACTTAAAACCA 1440
CN11_VP2        CCACCAGTTTATCCAAATGGTCAAATTTGGGATAAAGAATTTGATACTGACTTAAAACCA 1440
CN12_VP2        CCACCAGTTTATCCAAATGGTCAAATTTGGGATAAAGAATTTGATACTGACTTAAAACCA 1440
CN13_VP2        CCACCAGTTTATCCAAATGGTCAAATTTGGGATAAAGAATTTGATACTGACTTAAAACCA 1440
CN14_VP2        CCACCAGTTTATCCAAATGGTCAAATTTGGGATAAAGAATTTGATACTGACTTAAAACCA 1440
CN15_VP2        CCACCAGTTTATCCAAATGGTCAAATTTGGGATAAAGAATTTGATACTGACTTAAAACCA 1440
CN16_VP2        CCACCAGTTTATCCAAATGGTCAAATTTGGGATAAAGAATTTGATACTGACTTAAAACCA 1440
CN17_VP2        CCACCAGTTTATCCAAATGGTCAAATTTGGGATAAAGAATTTGATACTGACTTAAAACCA 1440
CN18_VP2        CCACCAGTTTATCCAAATGGTCAAATTTGGGATAAAGAATTTGATACTGACTTAAAACCA 1440
CN19_VP2        CCACCAGTTTATCCAAATGGTCAAATTTGGGATAAAGAATTTGATACTGACTTAAAACCA 1440
CN20_VP2        CCACCAGTTTATCCAAATGGTCAAATTTGGGATAAAGAATTTGATACTGACTTAAAACCA 1440
CN21_VP2        CCACCAGTTTATCCAAATGGTCAAATTTGGGATAAAGAATTTGATACTGACTTAAAACCA 1440
CN22_VP2        CCACCAGTTTATCCAAATGGTCAAATTTGGGATAAAGAATTTGATACTGACTTAAAACCA 1440
CN23_VP2        CCACCAGTTTATCCAAATGGTCAAATTTGGGATAAAGAATTTGATACTGACTTAAAACCA 1440
CN24_VP2        CCACCAGTTTATCCAAATGGTCAAATTTGGGATAAAGAATTTGATACTGACTTAAAACCA 1440
CN25_VP2        CCACCAGTTTATCCAAATGGTCAAATTTGGGATAAAGAATTTGATACTGACTTAAAACCA 1440
CN26_VP2        CCACCAGTTTATCCAAATGGTCAAATTTGGGATAAAGAATTTGATACTGACTTAAAACCA 1440
CN27_VP2        CCACCAGTTTATCCAAATGGTCAAATTTGGGATAAAGAATTTGATACTGACTTAAAACCA 1440
CN28_VP2        CCACCAGTTTATCCAAATGGTCAAATTTGGGATAAAGAATTTGATACTGACTTAAAACCA 1440
CN29_VP2        CCACCAGTTTATCCAAATGGTCAAATTTGGGATAAAGAATTTGATACTGACTTAAAACCA 1440
CN30_VP2        CCACCAGTTTATCCAAATGGTCAAATTTGGGATAAAGAATTTGATACTGACTTAAAACCA 1440
CN31_VP2        CCACCAGTTTATCCAAATGGTCAAATTTGGGATAAAGAATTTGATACTGACTTAAAACCA 1440
CN32_VP2        CCACCAGTTTATCCAAATGGTCAAATTTGGGATAAAGAATTTGATACTGACTTAAAACCA 1440
CN33_VP2        CCACCAGTTTATCCAAATGGTCAAATTTGGGATAAAGAATTTGATACTGACTTAAAACCA 1440
CN34_VP2        CCACCAGTTTATCCAAATGGTCAAATTTGGGATAAAGAATTTGATACTGACTTAAAACCA 1440
CN35_VP2        CCACCAGTTTATCCAAATGGTCAAATTTGGGATAAAGAATTTGATACTGACTTAAAACCA 1440
CN36_VP2        CCACCAGTTTATCCAAATGGTCAAATTTGGGATAAAGAATTTGATACTGACTTAAAACCA 1440
CN37_VP2        CCACCAGTTTATCCAAATGGTCAAATTTGGGATAAAGAATTTGATACTGACTTAAAACCA 1440
CN38_VP2        CCACCAGTTTATCCAAATGGTCAAATTTGGGATAAAGAATTTGATACTGACTTAAAACCA 1440
CN39_VP2        CCACCAGTTTATCCAAATGGTCAAATTTGGGATAAAGAATTTGATACTGACTTAAAACCA 1440
CN40_VP2        CCACCAGTTTATCCAAATGGTCAAATTTGGGATAAAGAATTTGATACTGACTTAAAACCA 1440
CK81_VP2        CCACCAGTTTATCCAAATGGTCAAATTTGGGATAAAGAATTTGATACTGACTTAAAACCA 1440
CK84_VP2        CCACCAGTTTATCCAAATGGTCAAATTTGGGATAAAGAATTTGATACTGACTTAAAACCA 1440
RVC50_VP2       CCACCAGTTTATCCAAATGGTCAAATTTGGGATAAAGAATTTGATACTGACTTAAAACCA 1440
                ************************************************************
```

```
CN7_VP2         AGACTTCATGTAAATGCACCATTTGTTTGTCAAAATAATTGTCCTGGTCAATTATTTGTA 1500
CN8_VP2         AGACTTCATGTAAATGCACCATTTGTTTGTCAAAATAATTGTCCTGGTCAATTATTTGTA 1500
CN10_VP2        AGACTTCATGTAAATGCACCATTTGTTTGTCAAAATAATTGTCCTGGTCAATTATTTGTA 1500
CN11_VP2        AGACTTCATGTAAATGCACCATTTGTTTGTCAAAATAATTGTCCTGGTCAATTATTTGTA 1500
CN12_VP2        AGACTTCATGTAAATGCACCATTTGTTTGTCAAAATAATTGTCCTGGTCAATTATTTGTA 1500
CN13_VP2        AGACTTCATGTAAATGCACCATTTGTTTGTCAAAATAATTGTCCTGGTCAATTATTTGTA 1500
CN14_VP2        AGACTTCATGTAAATGCACCATTTGTTTGTCAAAATAATTGTCCTGGTCAATTATTTGTA 1500
CN15_VP2        AGACTTCATGTAAATGCACCATTTGTTTGTCAAAATAATTGTCCTGGTCAATTATTTGTA 1500
CN16_VP2        AGACTTCATGTAAATGCACCATTTGTTTGTCAAAATAATTGTCCTGGTCAATTATTTGTA 1500
CN17_VP2        AGACTTCATGTAAATGCACCATTTGTTTGTCAAAATAATTGTCCTGGTCAATTATTTGTA 1500
CN18_VP2        AGACTTCATGTAAATGCACCATTTGTTTGTCAAAATAATTGTCCTGGTCAATTATTTGTA 1500
CN19_VP2        AGACTTCATGTAAATGCACCATTTGTTTGTCAAAATAATTGTCCTGGTCAATTATTTGTA 1500
CN20_VP2        AGACTTCATGTAAATGCACCATTTGTTTGTCAAAATAATTGTCCTGGTCAATTATTTGTA 1500
CN21_VP2        AGACTTCATGTAAATGCACCATTTGTTTGTCAAAATAATTGTCCTGGTCAATTATTTGTA 1500
CN22_VP2        AGACTTCATGTAAATGCACCATTTGTTTGTCAAAATAATTGTCCTGGTCAATTATTTGTA 1500
CN23_VP2        AGACTTCATGTAAATGCACCATTTGTTTGTCAAAATAATTGTCCTGGTCAATTATTTGTA 1500
CN24_VP2        AGACTTCATGTAAATGCACCATTTGTTTGTCAAAATAATTGTCCTGGTCAATTATTTGTA 1500
CN25_VP2        AGACTTCATGTAAATGCACCATTTGTTTGTCAAAATAATTGTCCTGGTCAATTATTTGTA 1500
CN26_VP2        AGACTTCATGTAAATGCACCATTTGTTTGTCAAAATAATTGTCCTGGTCAATTATTTGTA 1500
CN27_VP2        AGACTTCATGTAAATGCACCATTTGTTTGTCAAAATAATTGTCCTGGTCAATTATTTGTA 1500
CN28_VP2        AGACTTCATGTAAATGCACCATTTGTTTGTCAAAATAATTGTCCTGGTCAATTATTTGTA 1500
CN29_VP2        AGACTTCATGTAAATGCACCATTTGTTTGTCAAAATAATTGTCCTGGTCAATTATTTGTA 1500
CN30_VP2        AGACTTCATGTAAATGCACCATTTGTTTGTCAAAATAATTGTCCTGGTCAATTATTTGTA 1500
CN31_VP2        AGACTTCATGTAAATGCACCATTTGTTTGTCAAAATAATTGTCCTGGTCAATTATTTGTA 1500
CN32_VP2        AGACTTCATGTAAATGCACCATTTGTTTGTCAAAATAATTGTCCTGGTCAATTATTTGTA 1500
CN33_VP2        AGACTTCATGTAAATGCACCATTTGTTTGTCAAAATAATTGTCCTGGTCAATTATTTGTA 1500
CN34_VP2        AGACTTCATGTAAATGCACCATTTGTTTGTCAAAATAATTGTCCTGGTCAATTATTTGTA 1500
CN35_VP2        AGACTTCATGTAAATGCACCATTTGTTTGTCAAAATAATTGTCCTGGTCAATTATTTGTA 1500
CN36_VP2        AGACTTCATGTAAATGCACCATTTGTTTGTCAAAATAATTGTCCTGGTCAATTATTTGTA 1500
CN37_VP2        AGACTTCATGTAAATGCACCATTTGTTTGTCAAAATAATTGTCCTGGTCAATTATTTGTA 1500
CN38_VP2        AGACTTCATGTAAATGCACCATTTGTTTGTCAAAATAATTGTCCTGGTCAATTATTTGTA 1500
CN39_VP2        AGACTTCATGTAAATGCACCATTTGTTTGTCAAAATAATTGTCCTGGTCAATTATTTGTA 1500
CN40_VP2        AGACTTCATGTAAATGCACCATTTGTTTGTCAAAATAATTGTCCTGGTCAATTATTTGTA 1500
CK81_VP2        AGACTTCATGTAAATGCACCATTTGTTTGTCAAAATAATTGTCCTGGTCAATTATTTGTA 1500
CK84_VP2        AGACTTCATGTAAATGCACCATTTGTTTGTCAAAATAATTGTCCTGGTCAATTATTTGTA 1500
RVC50_VP2       AGACTTCATGTAAATGCACCATTTGTTTGTCAAAATAATTGTCCTGGTCAATTATTTGTA 1500
                ************************************************************

CN7_VP2         AAGGTTGCGCCTAATCTAACAAATGAATATGATCCTGATGCATCTGCTAATATGTCAAGA 1560
CN8_VP2         AAGGTTGCGCCTAATCTAACAAATGAATATGATCCTGATGCATCTGCTAATATGTCAAGA 1560
CN10_VP2        AAGGTTGCGCCTAATCTAACAAATGAATATGATCCTGATGCATCTGCTAATATGTCAAGA 1560
CN11_VP2        AAGGTTGCGCCTAATCTAACAAATGAATATGATCCTGATGCATCTGCTAATATGTCAAGA 1560
CN12_VP2        AAGGTTGCGCCTAATCTAACAAATGAATATGATCCTGATGCATCTGCTAATATGTCAAGA 1560
CN13_VP2        AAGGTTGCGCCTAATCTAACAAATGAATATGATCCTGATGCATCTGCTAATATGTCAAGA 1560
CN14_VP2        AAGGTTGCGCCTAATCTAACAAATGAATATGATCCTGATGCATCTGCTAATATGTCAAGA 1560
CN15_VP2        AAGGTTGCGCCTAATCTAACAAATGAATATGATCCTGATGCATCTGCTAATATGTCAAGA 1560
CN16_VP2        AAGGTTGCGCCTAATCTAACAAATGAATATGATCCTGATGCATCTGCTAATATGTCAAGA 1560
CN17_VP2        AAGGTTGCGCCTAATCTAACAAATGAATATGATCCTGATGCATCTGCTAATATGTCAAGA 1560
CN18_VP2        AAGGTTGCGCCTAATCTAACAAATGAATATGATCCTGATGCATCTGCTAATATGTCAAGA 1560
CN19_VP2        AAGGTTGCGCCTAATCTAACAAATGAATATGATCCTGATGCATCTGCTAATATGTCAAGA 1560
CN20_VP2        AAGGTTGCGCCTAATCTAACAAATGAATATGATCCTGATGCATCTGCTAATATGTCAAGA 1560
CN21_VP2        AAGGTTGCGCCTAATCTAACAAATGAATATGATCCTGATGCATCTGCTAATATGTCAAGA 1560
CN22_VP2        AAGGTTGCGCCTAATCTAACAAATGAATATGATCCTGATGCATCTGCTAATATGTCAAGA 1560
CN23_VP2        AAGGTTGCGCCTAATCTAACAAATGAATATGATCCTGATGCATCTGCTAATATGTCAAGA 1560
CN24_VP2        AAGGTTGCGCCTAATCTAACAAATGAATATGATCCTGATGCATCTGCTAATATGTCAAGA 1560
CN25_VP2        AAGGTTGCGCCTAATCTAACAAATGAATATGATCCTGATGCATCTGCTAATATGTCAAGA 1560
CN26_VP2        AAGGTTGCGCCTAATCTAACAAATGAATATGATCCTGATGCATCTGCTAATATGTCAAGA 1560
CN27_VP2        AAGGTTGCGCCTAATCTAACAAATGAATATGATCCTGATGCATCTGCTAATATGTCAAGA 1560
CN28_VP2        AAGGTTGCGCCTAATCTAACAAATGAATATGATCCTGATGCATCTGCTAATATGTCAAGA 1560
CN29_VP2        AAGGTTGCGCCTAATCTAACAAATGAATATGATCCTGATGCATCTGCTAATATGTCAAGA 1560
CN30_VP2        AAGGTTGCGCCTAATCTAACAAATGAATATGATCCTGATGCATCTGCTAATATGTCAAGA 1560
CN31_VP2        AAGGTTGCGCCTAATCTAACAAATGAATATGATCCTGATGCATCTGCTAATATGTCAAGA 1560
CN32_VP2        AAGGTTGCGCCTAATCTAACAAATGAATATGATCCTGATGCATCTGCTAATATGTCAAGA 1560
CN33_VP2        AAGGTTGCGCCTAATCTAACAAATGAATATGATCCTGATGCATCTGCTAATATGTCAAGA 1560
CN34_VP2        AAGGTTGCGCCTAATCTAACAAATGAATATGATCCTGATGCATCTGCTAATATGTCAAGA 1560
CN35_VP2        AAGGTTGCGCCTAATCTAACAAATGAATATGATCCTGATGCATCTGCTAATATGTCAAGA 1560
CN36_VP2        AAGGTTGCGCCTAATCTAACAAATGAATATGATCCTGATGCATCTGCTAATATGTCAAGA 1560
CN37_VP2        AAGGTTGCGCCTAATCTAACAAATGAATATGATCCTGATGCATCTGCTAATATGTCAAGA 1560
CN38_VP2        AAGGTTGCGCCTAATCTAACAAATGAATATGATCCTGATGCATCTGCTAATATGTCAAGA 1560
CN39_VP2        AAGGTTGCGCCTAATCTAACAAATGAATATGATCCTGATGCATCTGCTAATATGTCAAGA 1560
CN40_VP2        AAGGTTGCGCCTAATCTAACAAATGAATATGATCCTGATGCATCTGCTAATATGTCAAGA 1560
CK81_VP2        AAGGTTGCGCCTAATCTAACAAATGAATATGATCCTGATGCATCTGCTAATATGTCAAGA 1560
CK84_VP2        AAGGTTGCGCCTAATCTAACAAATGAATATGATCCTGATGCATCTGCTAATATGTCAAGA 1560
RVC50_VP2       AAGGTTGCGCCTAATCTAACAAATGAATATGATCCTGATGCATCTGCTAATATGTCAAGA 1560
                ************************************************************
```

```
CN7_VP2         ATTGTAACTTACTCAGATTTTTGGTGGAAAGGTAAATTAGTATTTAAAGCTAAACTAAGA 1620
CN8_VP2         ATTGTAACTTACTCAGATTTTTGGTGGAAAGGTAAATTAGTATTTAAAGCTAAACTAAGA 1620
CN10_VP2        ATTGTAACTTACTCAGATTTTTGGTGGAAAGGTAAATTAGTATTTAAAGCTAAACTAAGA 1620
CN11_VP2        ATTGTAACTTACTCAGATTTTTGGTGGAAAGGTAAATTAGTATTTAAAGCTAAACTAAGA 1620
CN12_VP2        ATTGTAACTTACTCAGATTTTTGGTGGAAAGGTAAATTAGTATTTAAAGCTAAACTAAGA 1620
CN13_VP2        ATTGTAACTTACTCAGATTTTTGGTGGAAAGGTAAATTAGTATTTAAAGCTAAACTAAGA 1620
CN14_VP2        ATTGTAACTTACTCAGATTTTTGGTGGAAAGGTAAATTAGTATTTAAAGCTAAACTAAGA 1620
CN15_VP2        ATTGTAACTTACTCAGATTTTTGGTGGAAAGGTAAATTAGTATTTAAAGCTAAACTAAGA 1620
CN16_VP2        ATTGTAACTTACTCAGATTTTTGGTGGAAAGGTAAATTAGTATTTAAAGCTAAACTAAGA 1620
CN17_VP2        ATTGTAACTTACTCAGATTTTTGGTGGAAAGGTAAATTAGTATTTAAAGCTAAACTAAGA 1620
CN18_VP2        ATTGTAACTTACTCAGATTTTTGGTGGAAAGGTAAATTAGTATTTAAAGCTAAACTAAGA 1620
CN19_VP2        ATTGTAACTTACTCAGATTTTTGGTGGAAAGGTAAATTAGTATTTAAAGCTAAACTAAGA 1620
CN20_VP2        ATTGTAACTTACTCAGATTTTTGGTGGAAAGGTAAATTAGTATTTAAAGCTAAACTAAGA 1620
CN21_VP2        ATTGTAACTTACTCAGATTTTTGGTGGAAAGGTAAATTAGTATTTAAAGCTAAACTAAGA 1620
CN22_VP2        ATTGTAACTTACTCAGATTTTTGGTGGAAAGGTAAATTAGTATTTAAAGCTAAACTAAGA 1620
CN23_VP2        ATTGTAACTTACTCAGATTTTTGGTGGAAAGGTAAATTAGTATTTAAAGCTAAACTAAGA 1620
CN24_VP2        ATTGTAACTTACTCAGATTTTTGGTGGAAAGGTAAATTAGTATTTAAAGCTAAACTAAGA 1620
CN25_VP2        ATTGTAACTTACTCAGATTTTTGGTGGAAAGGTAAATTAGTATTTAAAGCTAAACTAAGA 1620
CN26_VP2        ATTGTAACTTACTCAGATTTTTGGTGGAAAGGTAAATTAGTATTTAAAGCTAAACTAAGA 1620
CN27_VP2        ATTGTAACTTACTCAGATTTTTGGTGGAAAGGTAAATTAGTATTTAAAGCTAAACTAAGA 1620
CN28_VP2        ATTGTAACTTACTCAGATTTTTGGTGGAAAGGTAAATTAGTATTTAAAGCTAAACTAAGA 1620
CN29_VP2        ATTGTAACTTACTCAGATTTTTGGTGGAAAGGTAAATTAGTATTTAAAGCTAAACTAAGA 1620
CN30_VP2        ATTGTAACTTACTCAGATTTTTGGTGGAAAGGTAAATTAGTATTTAAAGCTAAACTAAGA 1620
CN31_VP2        ATTGTAACTTACTCAGATTTTTGGTGGAAAGGTAAATTAGTATTTAAAGCTAAACTAAGA 1620
CN32_VP2        ATTGTAACTTACTCAGATTTTTGGTGGAAAGGTAAATTAGTATTTAAAGCTAAACTAAGA 1620
CN33_VP2        ATTGTAACTTACTCAGATTTTTGGTGGAAAGGTAAATTAGTATTTAAAGCTAAACTAAGA 1620
CN34_VP2        ATTGTAACTTACTCAGATTTTTGGTGGAAAGGTAAATTAGTATTTAAAGCTAAACTAAGA 1620
CN35_VP2        ATTGTAACTTACTCAGATTTTTGGTGGAAAGGTAAATTAGTATTTAAAGCTAAACTAAGA 1620
CN36_VP2        ATTGTAACTTACTCAGATTTTTGGTGGAAAGGTAAATTAGTATTTAAAGCTAAACTAAGA 1620
CN37_VP2        ATTGTAACTTACTCAGATTTTTGGTGGAAAGGTAAATTAGTATTTAAAGCTAAACTAAGA 1620
CN38_VP2        ATTGTAACTTACTCAGATTTTTGGTGGAAAGGTAAATTAGTATTTAAAGCTAAACTAAGA 1620
CN39_VP2        ATTGTAACTTACTCAGATTTTTGGTGGAAAGGTAAATTAGTATTTAAAGCTAAACTAAGA 1620
CN40_VP2        ATTGTAACTTACTCAGATTTTTGGTGGAAAGGTAAATTAGTATTTAAAGCTAAACTAAGA 1620
CK81_VP2        ATTGTAACTTACTCAGATTTTTGGTGGAAAGGTAAATTAGTATTTAAAGCTAAACTAAGA 1620
CK84_VP2        ATTGTAACTTACTCAGATTTTTGGTGGAAAGGTAAATTAGTATTTAAAGCTAAACTAAGA 1620
RVC50_VP2       ATTGTAACTTACTCAGATTTTTGGTGGAAAGGTAAATTAGTATTTAAAGCTAAACTAAGA 1620
                ************************************************************

CN7_VP2         GCCTCTCATACTTGGAATCCAATTCAACAAATGAGTATTAATGTAGATAACCAATTTAAC 1680
CN8_VP2         GCCTCTCATACTTGGAATCCAATTCAACAAATGAGTATTAATGTAGATAACCAATTTAAC 1680
CN10_VP2        GCCTCTCATACTTGGAATCCAATTCAACAAATGAGTATTAATGTAGATAACCAATTTAAC 1680
CN11_VP2        GCCTCTCATACTTGGAATCCAATTCAACAAATGAGTATTAATGTAGATAACCAATTTAAC 1680
CN12_VP2        GCCTCTCATACTTGGAATCCAATTCAACAAATGAGTATTAATGTAGATAACCAATTTAAC 1680
CN13_VP2        GCCTCTCATACTTGGAATCCAATTCAACAAATGAGTATTAATGTAGATAACCAATTTAAC 1680
CN14_VP2        GCCTCTCATACTTGGAATCCAATTCAACAAATGAGTATTAATGTAGATAACCAATTTAAC 1680
CN15_VP2        GCCTCTCATACTTGGAATCCAATTCAACAAATGAGTATTAATGTAGATAACCAATTTAAC 1680
CN16_VP2        GCCTCTCATACTTGGAATCCAATTCAACAAATGAGTATTAATGTAGATAACCAATTTAAC 1680
CN17_VP2        GCCTCTCATACTTGGAATCCAATTCAACAAATGAGTATTAATGTAGATAACCAATTTAAC 1680
CN18_VP2        GCCTCTCATACTTGGAATCCAATTCAACAAATGAGTATTAATGTAGATAACCAATTTAAC 1680
CN19_VP2        GCCTCTCATACTTGGAATCCAATTCAACAAATGAGTATTAATGTAGATAACCAATTTAAC 1680
CN20_VP2        GCCTCTCATACTTGGAATCCAATTCAACAAATGAGTATTAATGTAGATAACCAATTTAAC 1680
CN21_VP2        GCCTCTCATACTTGGAATCCAATTCAACAAATGAGTATTAATGTAGATAACCAATTTAAC 1680
CN22_VP2        GCCTCTCATACTTGGAATCCAATTCAACAAATGAGTATTAATGTAGATAACCAATTTAAC 1680
CN23_VP2        GCCTCTCATACTTGGAATCCAATTCAACAAATGAGTATTAATGTAGATAACCAATTTAAC 1680
CN24_VP2        GCCTCTCATACTTGGAATCCAATTCAACAAATGAGTATTAATGTAGATAACCAATTTAAC 1680
CN25_VP2        GCCTCTCATACTTGGAATCCAATTCAACAAATGAGTATTAATGTAGATAACCAATTTAAC 1680
CN26_VP2        GCCTCTCATACTTGGAATCCAATTCAACAAATGAGTATTAATGTAGATAACCAATTTAAC 1680
CN27_VP2        GCCTCTCATACTTGGAATCCAATTCAACAAATGAGTATTAATGTAGATAACCAATTTAAC 1680
CN28_VP2        GCCTCTCATACTTGGAATCCAATTCAACAAATGAGTATTAATGTAGATAACCAATTTAAC 1680
CN29_VP2        GCCTCTCATACTTGGAATCCAATTCAACAAATGAGTATTAATGTAGATAACCAATTTAAC 1680
CN30_VP2        GCCTCTCATACTTGGAATCCAATTCAACAAATGAGTATTAATGTAGATAACCAATTTAAC 1680
CN31_VP2        GCCTCTCATACTTGGAATCCAATTCAACAAATGAGTATTAATGTAGATAACCAATTTAAC 1680
CN32_VP2        GCCTCTCATACTTGGAATCCAATTCAACAAATGAGTATTAATGTAGATAACCAATTTAAC 1680
CN33_VP2        GCCTCTCATACTTGGAATCCAATTCAACAAATGAGTATTAATGTAGATAACCAATTTAAC 1680
CN34_VP2        GCCTCTCATACTTGGAATCCAATTCAACAAATGAGTATTAATGTAGATAACCAATTTAAC 1680
CN35_VP2        GCCTCTCATACTTGGAATCCAATTCAACAAATGAGTATTAATGTAGATAACCAATTTAAC 1680
CN36_VP2        GCCTCTCATACTTGGAATCCAATTCAACAAATGAGTATTAATGTAGATAACCAATTTAAC 1680
CN37_VP2        GCCTCTCATACTTGGAATCCAATTCAACAAATGAGTATTAATGTAGATAACCAATTTAAC 1680
CN38_VP2        GCCTCTCATACTTGGAATCCAATTCAACAAATGAGTATTAATGTAGATAACCAATTTAAC 1680
CN39_VP2        GCCTCTCATACTTGGAATCCAATTCAACAAATGAGTATTAATGTAGATAACCAATTTAAC 1680
CN40_VP2        GCCTCTCATACTTGGAATCCAATTCAACAAATGAGTATTAATGTAGATAACCAATTTAAC 1680
CK81_VP2        GCCTCTCATACTTGGAATCCAATTCAACAAATGAGTATTAATGTAGATAACCAATTTAAC 1680
CK84_VP2        GCCTCTCATACTTGGAATCCAATTCAACAAATGAGTATTAATGTAGATAACCAATTTAAC 1680
RVC50_VP2       GCCTCTCATACTTGGAATCCAATTCAACAAATGAGTATTAATGTAGATAACCAATTTAAC 1680
                ************************************************************
```

```
CN7_VP2         TATGTACCAAGTAATATTGGAGGTATGAAAATTGTATATGAAAAATCTCAACTAGCACCT 1740
CN8_VP2         TATGTACCAAGTAATATTGGAGGTATGAAAATTGTATATGAAAAATCTCAACTAGCACCT 1740
CN10_VP2        TATGTACCAAGTAATATTGGAGGTATGAAAATTGTATATGAAAAATCTCAACTAGCACCT 1740
CN11_VP2        TATGTACCAAGTAATATTGGAGGTATGAAAATTGTATATGAAAAATCTCAACTAGCACCT 1740
CN12_VP2        TATGTACCAAGTAATATTGGAGGTATGAAAATTGTATATGAAAAATCTCAACTAGCACCT 1740
CN13_VP2        TATGTACCAAGTAATATTGGAGGTATGAAAATTGTATATGAAAAATCTCAACTAGCACCT 1740
CN14_VP2        TATGTACCAAGTAATATTGGAGGTATGAAAATTGTATATGAAAAATCTCAACTAGCACCT 1740
CN15_VP2        TATGTACCAAGTAATATTGGAGGTATGAAAATTGTATATGAAAAATCTCAACTAGCACCT 1740
CN16_VP2        TATGTACCAAGTAATATTGGAGGTATGAAAATTGTATATGAAAAATCTCAACTAGCACCT 1740
CN17_VP2        TATGTACCAAGTAATATTGGAGGTATGAAAATTGTATATGAAAAATCTCAACTAGCACCT 1740
CN18_VP2        TATGTACCAAGTAATATTGGAGGTATGAAAATTGTATATGAAAAATCTCAACTAGCACCT 1740
CN19_VP2        TATGTACCAAGTAATATTGGAGGTATGAAAATTGTATATGAAAAATCTCAACTAGCACCT 1740
CN20_VP2        TATGTACCAAGTAATATTGGAGGTATGAAAATTGTATATGAAAAATCTCAACTAGCACCT 1740
CN21_VP2        TATGTACCAAGTAATATTGGAGGTATGAAAATTGTATATGAAAAATCTCAACTAGCACCT 1740
CN22_VP2        TATGTACCAAGTAATATTGGAGGTATGAAAATTGTATATGAAAAATCTCAACTAGCACCT 1740
CN23_VP2        TATGTACCAAGTAATATTGGAGGTATGAAAATTGTATATGAAAAATCTCAACTAGCACCT 1740
CN24_VP2        TATGTACCAAGTAATATTGGAGGTATGAAAATTGTATATGAAAAATCTCAACTAGCACCT 1740
CN25_VP2        TATGTACCAAGTAATATTGGAGGTATGAAAATTGTATATGAAAAATCTCAACTAGCACCT 1740
CN26_VP2        TATGTACCAAGTAATATTGGAGGTATGAAAATTGTATATGAAAAATCTCAACTAGCACCT 1740
CN27_VP2        TATGTACCAAGTAATATTGGAGGTATGAAAATTGTATATGAAAAATCTCAACTAGCACCT 1740
CN28_VP2        TATGTACCAAGTAATATTGGAGGTATGAAAATTGTATATGAAAAATCTCAACTAGCACCT 1740
CN29_VP2        TATGTACCAAGTAATATTGGAGGTATGAAAATTGTATATGAAAAATCTCAACTAGCACCT 1740
CN30_VP2        TATGTACCAAGTAATATTGGAGGTATGAAAATTGTATATGAAAAATCTCAACTAGCACCT 1740
CN31_VP2        TATGTACCAAGTAATATTGGAGGTATGAAAATTGTATATGAAAAATCTCAACTAGCACCT 1740
CN32_VP2        TATGTACCAAGTAATATTGGAGGTATGAAAATTGTATATGAAAAATCTCAACTAGCACCT 1740
CN33_VP2        TATGTACCAAGTAATATTGGAGGTATGAAAATTGTATATGAAAAATCTCAACTAGCACCT 1740
CN34_VP2        TATGTACCAAGTAATATTGGAGGTATGAAAATTGTATATGAAAAATCTCAACTAGCACCT 1740
CN35_VP2        TATGTACCAAGTAATATTGGAGGTATGAAAATTGTATATGAAAAATCTCAACTAGCACCT 1740
CN36_VP2        TATGTACCAAGTAATATTGGAGGTATGAAAATTGTATATGAAAAATCTCAACTAGCACCT 1740
CN37_VP2        TATGTACCAAGTAATATTGGAGGTATGAAAATTGTATATGAAAAATCTCAACTAGCACCT 1740
CN38_VP2        TATGTACCAAGTAATATTGGAGGTATGAAAATTGTATATGAAAAATCTCAACTAGCACCT 1740
CN39_VP2        TATGTACCAAGTAATATTGGAGGTATGAAAATTGTATATGAAAAATCTCAACTAGCACCT 1740
CN40_VP2        TATGTACCAAGTAATATTGGAGGTATGAAAATTGTATATGAAAAATCTCAACTAGCACCT 1740
CK81_VP2        TATGTACCAAGTAATATTGGAGGTATGAAAATTGTATATGAAAAATCTCAACTAGCACCT 1740
CK84_VP2        TATGTACCAAGTAATATTGGAGGTATGAAAATTGTATATGAAAAATCTCAACTAGCACCT 1740
RVC50_VP2       TATGTACCAAGTAATATTGGAGGTATGAAAATTGTATATGAAAAATCTCAACTAGCACCT 1740
                ************************************************************

CN7_VP2         AGAAAATTATATTAA 1755
CN8_VP2         AGAAAATTATATTAA 1755
CN10_VP2        AGAAAATTATATTAA 1755
CN11_VP2        AGAAAATTATATTAA 1755
CN12_VP2        AGAAAATTATATTAA 1755
CN13_VP2        AGAAAATTATATTAA 1755
CN14_VP2        AGAAAATTATATTAA 1755
CN15_VP2        AGAAAATTATATTAA 1755
CN16_VP2        AGAAAATTATATTAA 1755
CN17_VP2        AGAAAATTATATTAA 1755
CN18_VP2        AGAAAATTATATTAA 1755
CN19_VP2        AGAAAATTATATTAA 1755
CN20_VP2        AGAAAATTATATTAA 1755
CN21_VP2        AGAAAATTATATTAA 1755
CN22_VP2        AGAAAATTATATTAA 1755
CN23_VP2        AGAAAATTATATTAA 1755
CN24_VP2        AGAAAATTATATTAA 1755
CN25_VP2        AGAAAATTATATTAA 1755
CN26_VP2        AGAAAATTATATTAA 1755
CN27_VP2        AGAAAATTATATTAA 1755
CN28_VP2        AGAAAATTATATTAA 1755
CN29_VP2        AGAAAATTATATTAA 1755
CN30_VP2        AGAAAATTATATTAA 1755
CN31_VP2        AGAAAATTATATTAA 1755
CN32_VP2        AGAAAATTATATTAA 1755
CN33_VP2        AGAAAATTATATTAA 1755
CN34_VP2        AGAAAATTATATTAA 1755
CN35_VP2        AGAAAATTATATTAA 1755
CN36_VP2        AGAAAATTATATTAA 1755
CN37_VP2        AGAAAATTATATTAA 1755
CN38_VP2        AGAAAATTATATTAA 1755
CN39_VP2        AGAAAATTATATTAA 1755
CN40_VP2        AGAAAATTATATTAA 1755
CK81_VP2        AGAAAATTATATTAA 1755
CK84_VP2        AGAAAATTATATTAA 1755
RVC50_VP2       AGAAAATTATATTAA 1755
                ***************
```
