# Supplementary material for: Molecular Investigation of Canine Parvovirus-2 (CPV-2) Outbreak in Nevis Island: Analysis of the Nearly Complete Genomes of CPV-2 Strains from the Caribbean Region
Source: Viruses. 2021 Jun 6;13(6):1083. doi: 10.3390/v13061083 (PMC8227521; doi:10.3390/v13061083)

Supplementary material S4. Gross **(A)** and microscopic **(B)** lesions observed in the small intestine of a dog with hemorrhagic gastroenteritis that eventually died on the island of St. Kitts. Small intestinal scrapings collected during necropsy tested positive for canine parvovirus-2 (CPV-2) by PCR. Analysis of the complete deduced VP2 amino acid sequence identified the CPV-2 strain as a new CPV-2a variant. **(A)** Grossly, the lumen of small intestine contained red-tinged fluid, and mucosal surfaces were dark red-diffuse hemorrhagic enteritis. **(B)** Microscopically, the intestinal architecture was distorted. The remnant intestinal crypts (asterisks) were distended, and contained sloughed and necrotic cellular debris - crypt necrosis, lined by hypertrophic enterocytes - attempts at regeneration (arrowheads). Viable and degenerate neutrophils infiltrated the lamina propria (arrows).

(A) **(B)**

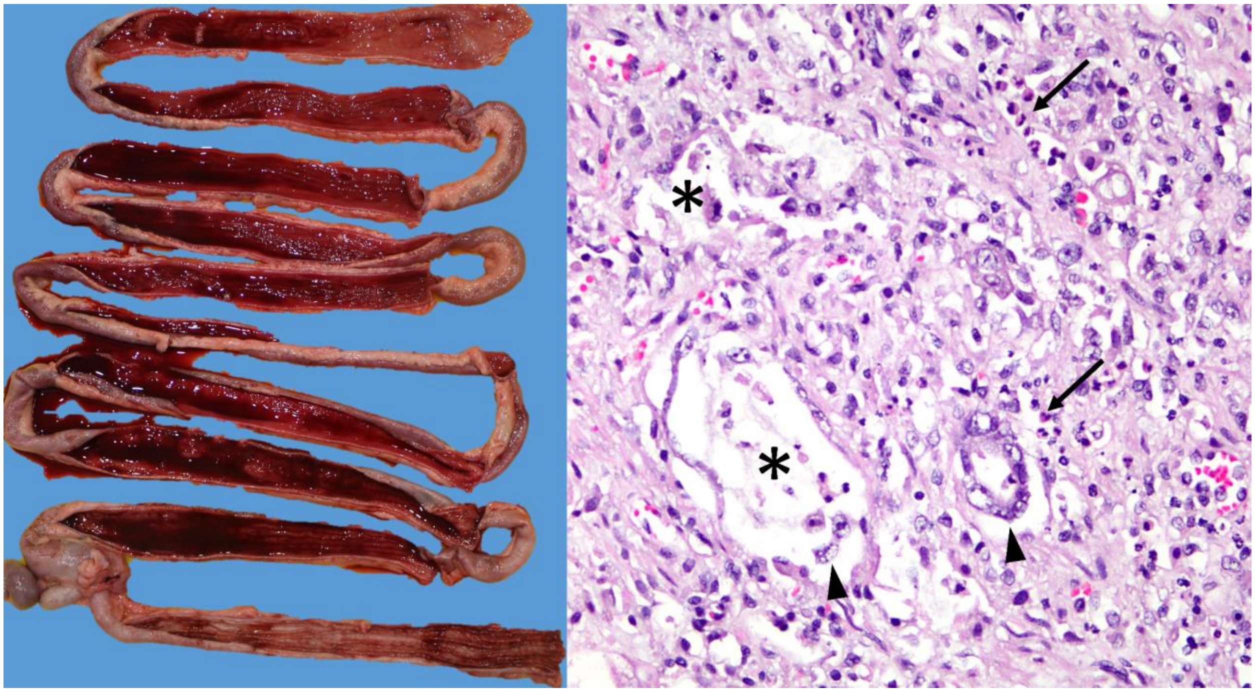

Supplement: Supplementary file 1 [file viruses-13-01083-s001.zip › Supplementary materials_R1/Supplementary material S4.pdf]
