# Supplementary material for: Molecular Investigation of Canine Parvovirus-2 (CPV-2) Outbreak in Nevis Island: Analysis of the Nearly Complete Genomes of CPV-2 Strains from the Caribbean Region
Source: Viruses. 2021 Jun 6;13(6):1083. doi: 10.3390/v13061083 (PMC8227521; doi:10.3390/v13061083)
Supplement: Supplementary file 1 [file viruses-13-01083-s001.zip › Supplementary materials_R1/Supplementary material S5.pdf]

Supplementary material S5. Nucleotide (nt) mismatches between the nearly complete genome sequences (4269 nt, spanning all the coding regions) of canine parvovirus-2 (CPV-2) strain CPV12 (representing the earliest CPV-2 strains from the late 1970s) and CPV-2 strains detected on St. Kitts in 2016 (strain RVC50) and Nevis in 2020 (strains CN10, CN14 and CN20). Synonymous and nonsynonymous mutations that differed in a nt from the CPV12 sequence is shown with green and red, respectively. A '*' denotes an identical nt residue. Numbers to the right indicate the positions of the nt for respective CPV-2 strains.

```
CPV12_USA_1978          ATGTCTGGCAACCAGTATACTGAGGAAGTTATGGAGGGAGTAAATTGGTTAAAGAAACAT 60
RVC50_St._Kitts_2016    ATGTCTGGCAACCAGTATACTGAGGAAGTTATGGAGGGAGTAAATTGGTTAAAGAAACAT 60
CN10_Nevis_2020         ATGTCTGGCAACCAGTATACTGAGGAAGTTATGGAGGGAGTAAATTGGTTAAAGAAACAT 60
CN14_Nevis_2020         ATGTCTGGCAACCAGTATACTGAGGAAGTTATGGAGGGAGTAAATTGGTTAAAGAAACAT 60
CN20_Nevis_2020         ATGTCTGGCAACCAGTATACTGAGGAAGTTATGGAGGGAGTAAATTGGTTAAAGAAACAT 60
                        ************************************************************

CPV12_USA_1978          GCAGAAAATGAAGCATTTTCGTTTGTTTTTAAATGTGACAACGTCCAACTAAATGGAAAG 120
RVC50_St._Kitts_2016    GCAGAAAATGAAGCATTTTCGTTTGTTTTTAAATGTGACAACGTCCAACTAAATGGAAAG 120
CN10_Nevis_2020         GCAGAAAATGAAGCATTTTCGTTTGTTTTTAAATGTGACAACGTCCAACTAAATGGAAAG 120
CN14_Nevis_2020         GCAGAAAATGAAGCATTTTCGTTTGTTTTTAAATGTGACAACGTCCAACTAAATGGAAAG 120
CN20_Nevis_2020         GCAGAAAATGAAGCATTTTCGTTTGTTTTTAAATGTGACAACGTCCAACTAAATGGAAAG 120
                        ************************************************************

CPV12_USA_1978          GATGTTCGCTGGAACAACTATACCAAACCAATTCAAAATGAAGAGCTAACATCTTTAATT 180
RVC50_St._Kitts_2016    GATGTTCGCTGGAACAACTATACCAAACCAATTCAAAATGAAGAGCTAACATCTTTAATT 180
CN10_Nevis_2020         GATGTTCGCTGGAACAACTATACCAAACCAATTCAAAATGAAGAGCTAACATCTTTAATT 180
CN14_Nevis_2020         GATGTTCGCTGGAACAACTATACCAAACCAATTCAAAATGAAGAGCTAACATCTTTAATT 180
CN20_Nevis_2020         GATGTTCGCTGGAACAACTATACCAAACCAATTCAAAATGAAGAGCTAACATCTTTAATT 180
                        ************************************************************

CPV12_USA_1978          AGAGGAGCACAAACAGCAATGGATCAAACCGAAGAAGAAGAAATGGACTGGGAATCGGAA 240
RVC50_St._Kitts_2016    AGAGGAGCACAAACAGCAATGGATCAAACCGAAGAAGAAGAAATGGACTGGGAATCGGAA 240
CN10_Nevis_2020         AGAGGAGCACAAACAGCAATGGATCAAACCGAAGAAGAAGAAATGGACTGGGAATCGGAA 240
CN14_Nevis_2020         AGAGGAGCACAAACAGCAATGGATCAAACCGAAGAAGAAGAAATGGACTGGGAATCGGAA 240
CN20_Nevis_2020         AGAGGAGCACAAACAGCAATGGATCAAACCGAAGAAGAAGAAATGGACTGGGAATCGGAA 240
                        ************************************************************

CPV12_USA_1978          GTTGATAGTCTCGCCAAAAAGCAAGTACAAACTTTTGATGCATTAATTAAAAAATGTCTT 300
RVC50_St._Kitts_2016    GTTGATAGTCTCGCCAAAAAGCAAGTACAAACTTTTGATGCATTAATTAAAAAATGTCTT 300
CN10_Nevis_2020         GTTGATAGTCTCGCCAAAAAGCAAGTACAAACTTTTGATGCATTAATTAAAAAATGTCTT 300
CN14_Nevis_2020         GTTGATAGTCTCGCCAAAAAGCAAGTACAAACTTTTGATGCATTAATTAAAAAATGTCTT 300
CN20_Nevis_2020         GTTGATAGTCTCGCCAAAAAGCAAGTACAAACTTTTGATGCATTAATTAAAAAATGTCTT 300
                        ************************************************************

CPV12_USA_1978          TTTGAAGTCTTTGTTTCTAAAAATATAGAACCAAATGAATGTGTTTGGTTTATTCAACAT 360
RVC50_St._Kitts_2016    TTTGAAGTCTTTGTTTCTAAAAATATAGAACCAAATGAATGTGTTTGGTTTATTCAACAT 360
CN10_Nevis_2020         TTTGAAGTCTTTGTTTCTAAAAATATAGAACCAAATGAATGTGTTTGGTTTATTCAACAT 360
CN14_Nevis_2020         TTTGAAGTCTTTGTTTCTAAAAATATAGAACCAAATGAATGTGTTTGGTTTATTCAACAT 360
CN20_Nevis_2020         TTTGAAGTCTTTGTTTCTAAAAATATAGAACCAAATGAATGTGTTTGGTTTATTCAACAT 360
                        ************************************************************

CPV12_USA_1978          GAATGGGGAAAAGATCAAGGCTGGCATTGTCATGTTTTACTTCATAGTAAGAACTTACAA 420
RVC50_St._Kitts_2016    GAATGGGGAAAAGATCAAGGCTGGCATTGTCATGTTTTACTTCATAGTAAGAACTTACAA 420
CN10_Nevis_2020         GAATGGGGAAAAGATCAAGGCTGGCATTGTCATGTTTTACTTCATAGTAAGAACTTACAA 420
CN14_Nevis_2020         GAATGGGGAAAAGATCAAGGCTGGCATTGTCATGTTTTACTTCATAGTAAGAACTTACAA 420
CN20_Nevis_2020         GAATGGGGAAAAGATCAAGGCTGGCATTGTCATGTTTTACTTCATAGTAAGAACTTACAA 420
                        ************************************************************

CPV12_USA_1978          CAAGCAACTGGTAAATGGCTACGCAGACAAATGAATATGTATTGGAGTAGATGGTTGGTG 480
RVC50_St._Kitts_2016    CAAGCAACTGGTAAATGGCTACGCAGACAAATGAATATGTATTGGAGTAGATGGTTGGTG 480
CN10_Nevis_2020         CAAGCAACTGGTAAATGGCTACGCAGACAAATGAATATGTATTGGAGTAGATGGTTGGTG 480
CN14_Nevis_2020         CAAGCAACTGGTAAATGGCTACGCAGACAAATGAATATGTATTGGAGTAGATGGTTGGTG 480
CN20_Nevis_2020         CAAGCAACTGGTAAATGGCTACGCAGACAAATGAATATGTATTGGAGTAGATGGTTGGTG 480
                        ************************************************************
```

```
CPV12_USA_1978          ACTCTTTGTTCGGTAAACTTAACACCAACTGAAAAGATTAAGCTCAGAGAAATTGCAGAA 540
RVC50_St._Kitts_2016    ACTCTTTGTTCGGTAAACTTAACACCAACTGAAAAAATTAAGCTCAGAGAAATTGCAGAA 540
CN10_Nevis_2020         ACTCTTTGTTCGGTAAACTTAACACCAACTGAAAAAATTAAGCTCAGAGAAATTGCAGAA 540
CN14_Nevis_2020         ACTCTTTGTTCGGTAAACTTAACACCAACTGAAAAAATTAAGCTCAGAGAAATTGCAGAA 540
CN20_Nevis_2020         ACTCTTTGTTCGGTAAACTTAACACCAACTGAAAAAATTAAGCTCAGAGAAATTGCAGAA 540
                        *********************************** ************************

CPV12_USA_1978          GATAGTGAATGGGTGACTATATTAACATACAGACATAAGCAAACAAAAAAAGACTATGTT 600
RVC50_St._Kitts_2016    GATAGTGAATGGGTGACTATACTAACATACAGACATAAGCAAACAAAAAAAGACTATGTT 600
CN10_Nevis_2020         GATAGTGAATGGGTGACTATACTAACATACAGACATAAGCAAACAAAAAAAGACTATGTT 600
CN14_Nevis_2020         GATAGTGAATGGGTGACTATACTAACATACAGACATAAGCAAACAAAAAAAGACTATGTT 600
CN20_Nevis_2020         GATAGTGAATGGGTGACTATACTAACATACAGACATAAGCAAACAAAAAAAGACTATGTT 600
                        ********************* **************************************

CPV12_USA_1978          AAAATGGTTCATTTTGGAAATATGATAGCATATTACTTTTTAACAAAGAAAAAAATTGTC 660
RVC50_St._Kitts_2016    AAAATGGTTCATTTTGGAAATATGATAGCATATTACTTTTTAACAAAGAAAAAAATTGTC 660
CN10_Nevis_2020         AAAATGGTTCATTTTGGAAATATGATAGCATATTACTTTTTAACAAAGAAAAAAATTGTC 660
CN14_Nevis_2020         AAAATGGTTCATTTTGGAAATATGATAGCATATTACTTTTTAACAAAGAAAAAAATTGTC 660
CN20_Nevis_2020         AAAATGGTTCATTTTGGAAATATGATAGCATATTACTTTTTAACAAAGAAAAAAATTGTC 660
                        ************************************************************

CPV12_USA_1978          CACATGACAAAAGAAAGTGGCTATTTTTTAAGTACTGATTCTGGTTGGAAATTTAACTTT 720
RVC50_St._Kitts_2016    CACATGACAAAAGAAAGTGGCTATTTTTTAAGTACTGATTCTGGTTGGAAATTTAACTTT 720
CN10_Nevis_2020         CACATGACAAAAGAAAGTGGCTATTTTTTAAGTACTGATTCTGGTTGGAAATTTAACTTT 720
CN14_Nevis_2020         CACATGACAAAAGAAAGTGGCTATTTTTTAAGTACTGATTCTGGTTGGAAATTTAACTTT 720
CN20_Nevis_2020         CACATGACAAAAGAAAGTGGCTATTTTTTAAGTACTGATTCTGGTTGGAAATTTAACTTT 720
                        ************************************************************

CPV12_USA_1978          ATGAAGTATCAAGACAGACAAATTGTCAGCACACTTTACACTGAACAAATGAAACCAGAA 780
RVC50_St._Kitts_2016    ATGAAGTATCAAGACAGACAAATTGTCAGCACACTTTACACTGAACAAATGAAACCAGAA 780
CN10_Nevis_2020         ATGAAATATCAAGACAGACAAATTGTCAGCACGCTTTACACTGAACAAATGAAACCAGAA 780
CN14_Nevis_2020         ATGAAGTATCAAGACAGACAAATTGTCAGCACACTTTACACTGAACAAATGAAACCAGAA 780
CN20_Nevis_2020         ATGAAATATCAAGACAGACAAATTGTCAGCACGCTTTACACTGAACAAATGAAACCAGAA 780
                        ***** ************************** ***************************

CPV12_USA_1978          ACCGTTGAAACCACAGTGACGACAGCACAGGAAACAAAGCGCGGGAGAATTCAAACTAAA 840
RVC50_St._Kitts_2016    ACCGTTGAAACCACAGTGACGACAGCACAGGAAACAAAGCGCGGGAGAATTCAAACTAAA 840
CN10_Nevis_2020         ACCGTTGAAACCACAGTGACGACAGCACAGGAAACAAAGCGCGGGAGAATTCAAACTAAA 840
CN14_Nevis_2020         ACCGTTGAAACCACAGTGACGACAGCACAGGAAACAAAGCGCGGGAGAATTCAAACTAAA 840
CN20_Nevis_2020         ACCGTTGAAACCACAGTGACGACAGCACAGGAAACAAAGCGCGGGAGAATTCAAACTAAA 840
                        ************************************************************

CPV12_USA_1978          AAGGAAGTGTCAATCAAATGTACTTTGCGGGACTTGGTTAGTAAAAGAGTAACATCACCT 900
RVC50_St._Kitts_2016    AAGGAAGTGTCAATCAAATGTACTTTGCGGGACTTGGTTAGTAAAAGAGTAACATCACCT 900
CN10_Nevis_2020         AAGGAAGTGTCAATCAAATGTACTTTGCGGGACTTGGTTAGTAAAAGAGTAACATCACCT 900
CN14_Nevis_2020         AAGGAAGTGTCAATCAAATGTACTTTGCGGGACTTGGTTAGTAAAAGAGTAACATCACCT 900
CN20_Nevis_2020         AAGGAAGTGTCAATCAAATGTACTTTGCGGGACTTGGTTAGTAAAAGAGTAACATCACCT 900
                        ************************************************************

CPV12_USA_1978          GAAGACTGGATGATGTTACAACCAGATAGTTATATTGAAATGATGGCACAACCAGGAGGT 960
RVC50_St._Kitts_2016    GAAGACTGGATGATGTTACAACCAGATAGTTATATTGAAATGATGGCACAACCAGGAGGT 960
CN10_Nevis_2020         GAAGACTGGATGATGTTACAACCAGATAGTTATATTGAAATGATGGCACAACCAGGAGGT 960
CN14_Nevis_2020         GAAGACTGGATGATGTTACAACCAGATAGTTATATTGAAATGATGGCACAACCAGGAGGT 960
CN20_Nevis_2020         GAAGACTGGATGATGTTACAACCAGATAGTTATATTGAAATGATGGCACAACCAGGAGGT 960
                        ************************************************************
```

```
CPV12_USA_1978          GAAAATCTTTTAAAAAATACACTTGAAATTTGTACTTTGACTTTAGCAAGAACAAAAACA 1020
RVC50_St._Kitts_2016    GAAAATCTTTTAAAAAATACACTTGAAATTTGTACTTTGACTTTAGCAAGAACAAAAACA 1020
CN10_Nevis_2020         GAAAATCTTTTAAAAAATACACTTGAAATTTGTACTTTGACTTTAGCAAGAACAAAAACA 1020
CN14_Nevis_2020         GAAAATCTTTTAAAAAATACACTTGAAATTTGTACTTTGACTTTAGCAAGAACAAAAACA 1020
CN20_Nevis_2020         GAAAATCTTTTAAAAAATACACTTGAAATTTGTACTTTGACTTTAGCAAGAACAAAAACA 1020
                        ************************************************************

CPV12_USA_1978          GCATTTGAATTAATACTTGAAAAAGCAGATAATACTAAACTAACTAACTTTGATCTTGCA 1080
RVC50_St._Kitts_2016    GCATTTGAATTAATACTTGAAAAAGCAGATAATACTAAACTAACTAACTTTGATCTTGCA 1080
CN10_Nevis_2020         GCATTTGAATTAATACTTGAAAAAGCAGATAATACTAAACTAACTAACTTTGATCTTGCA 1080
CN14_Nevis_2020         GCATTTGAATTAATACTTGAAAAAGCAGATAATACTAAACTAACTAACTTTGATCTTGCA 1080
CN20_Nevis_2020         GCATTTGAATTAATACTTGAAAAAGCAGATAATACTAAACTAACTAACTTTGATCTTGCA 1080
                        ************************************************************

CPV12_USA_1978          AATTCTAGAACATGTCAAATTTTTAGAATGCACGGATGGAATTGGATTAAAGTTTGTCAC 1140
RVC50_St._Kitts_2016    AATTCTAGAACATGTCAAATTTTTAGAATGCACGGATGGAATTGGATTAAAGTTTGTCAC 1140
CN10_Nevis_2020         AATTCTAGAACATGTCAAATTTTCAGAATGCACGGATGGAATTGGATTAAAGTTTGTCAC 1140
CN14_Nevis_2020         AATTCTAGAACATGTCAAATTTTTAGAATGCACGGATGGAATTGGATTAAAGTTTGTCAC 1140
CN20_Nevis_2020         AATTCTAGAACATGTCAAATTTTCAGAATGCACGGATGGAATTGGATTAAAGTTTGTCAC 1140
                        *********************** ************************************

CPV12_USA_1978          GCTATAGCATGTGTTTTAAATAGACAAGGTGGTAAAAGAAATACAGTTCTTTTTCATGGA 1200
RVC50_St._Kitts_2016    GCTATAGCATGTGTTTTAAATAGACAAGGTGGTAAAAGAAATACAGTTCTTTTTCATGGA 1200
CN10_Nevis_2020         GCTATAGCATGTGTTTTAAATAGGCAAGGTGGTAAAAGAAATACAGTTCTTTTTCATGGA 1200
CN14_Nevis_2020         GCTATAGCATGTGTTTTAAATAGACAAGGTGGTAAAAGAAATACAGTTCTTTTTCATGGA 1200
CN20_Nevis_2020         GCTATAGCATGTGTTTTAAATAGGCAAGGTGGTAAAAGAAATACAGTTCTTTTTCATGGA 1200
                        *********************** ************************************

CPV12_USA_1978          CCAGCAAGTACAGGAAAATCTATCATTGCTCAAGCCATAGCACAAGCTGTGGGTAATGTT 1260
RVC50_St._Kitts_2016    CCAGCAAGCACAGGAAAATCTATCATTGCTCAAGCCATAGCACAAGCTGTGGGTAATGTT 1260
CN10_Nevis_2020         CCAGCAAGCACAGGAAAATCTATCATTGCTCAAGCCATAGCACAAGCTGTGGGTAATGTT 1260
CN14_Nevis_2020         CCAGCAAGCACAGGAAAATCTATCATTGCTCAAGCCATAGCACAAGCTGTGGGTAATGTT 1260
CN20_Nevis_2020         CCAGCAAGCACAGGAAAATCTATCATTGCTCAAGCCATAGCACAAGCTGTGGGTAATGTT 1260
                        ******** ***************************************************

CPV12_USA_1978          GGTTGTTATAATGCAGCAAATGTAAATTTTCCATTTAATGACTGTACCAATAAAAATTTA 1320
RVC50_St._Kitts_2016    GGTTGTTATAATGCAGCAAATGTAAATTTTCCATTTAATGACTGCACCAATAAAAATTTA 1320
CN10_Nevis_2020         GGTTGTTATAATGCAGCAAATGTAAATTTTCCATTTAATGACTGCACCAATAAAAATTTA 1320
CN14_Nevis_2020         GGTTGTTATAATGCAGCAAATGTAAATTTTCCATTTAATGACTGCACCAATAAAAATTTA 1320
CN20_Nevis_2020         GGTTGTTATAATGCAGCAAATGTAAATTTTCCATTTAATGACTGCACCAATAAAAATTTA 1320
                        ******************************************** ***************

CPV12_USA_1978          ATTTGGATTGAAGAAGCTGGTAACTTTGGTCAACAAGTTAATCAATTTAAAGCAATTTGT 1380
RVC50_St._Kitts_2016    ATTTGGATTGAAGAAGCTGGTAACTTTGGTCAACAAGTTAATCAATTTAAAGCAATTTGT 1380
CN10_Nevis_2020         ATTTGGATTGAAGAAGCTGGTAACTTTGGTCAACAAGTTAATCAATTTAAAGCAATTTGT 1380
CN14_Nevis_2020         ATTTGGATTGAAGAAGCTGGTAACTTTGGTCAACAAGTTAATCAATTTAAAGCAATTTGT 1380
CN20_Nevis_2020         ATTTGGATTGAAGAAGCTGGTAACTTTGGTCAACAAGTTAATCAATTTAAAGCAATTTGT 1380
                        ************************************************************

CPV12_USA_1978          TCTGGACAAACAATTAGAATTGATCAAAAAGGTAAAGGAAGTAAGCAAATTGAACCAACT 1440
RVC50_St._Kitts_2016    TCTGGACAAACAATTAGAATTGATCAAAAAGGTAAAGGAAGTAAGCAAATTGAACCAACT 1440
CN10_Nevis_2020         TCTGGACAAACAATTAGAATTGATCAAAAAGGTAAAGGAAGTAAGCAAATTGAACCAACT 1440
CN14_Nevis_2020         TCTGGACAAACAATTAGAATTGATCAAAAAGGTAAAGGAAGTAAGCAAATTGAACCAACT 1440
CN20_Nevis_2020         TCTGGACAAACAATTAGAATTGATCAAAAAGGTAAAGGAAGTAAGCAAATTGAACCAACT 1440
                        ************************************************************
```

```
CPV12_USA_1978          CCAGTAATTATGACAACTAATGAAAATATAACAATTGTGAGAATTGGATGTGAAGAAAGA 1500
RVC50_St._Kitts_2016    CCAGTAATTATGACAACTAATGAAAATATAACAATTGTGAGAATTGGATGTGAAGAAAGA 1500
CN10_Nevis_2020         CCAGTAATTATGACAACTAATGAAAATATAACAATTGTGAGAATTGGATGTGAAGAAAGA 1500
CN14_Nevis_2020         CCAGTAATTATGACAACTAATGAAAATATAACAATTGTGAGAATTGGATGTGAAGAAAGA 1500
CN20_Nevis_2020         CCAGTAATTATGACAACTAATGAAAATATAACAATTGTGAGAATTGGATGTGAAGAAAGA 1500
                        ************************************************************

CPV12_USA_1978          CCTGAACATACACAACCAATAAGAGACAGAATGTTGAACATTAAGTTAGTATGTAAGCTT 1560
RVC50_St._Kitts_2016    CCTGAACATACACAACCAATAAGAGACAGAATGTTGAACATTAAGTTAGTATGTAAGCTT 1560
CN10_Nevis_2020         CCTGAACATACACAACCAATAAGAGACAGAATGTTGAACATTAAGTTAGTATGTAAGCTT 1560
CN14_Nevis_2020         CCTGAACATACACAACCAATAAGAGACAGAATGTTGAACATTAAGTTAGTATGTAAGCTT 1560
CN20_Nevis_2020         CCTGAACATACACAACCAATAAGAGACAGAATGTTGAACATTAAGTTAGTATGTAAGCTT 1560
                        ************************************************************

CPV12_USA_1978          CCAGGAGACTTTGGTTTGGTTGATAAAGAAGAATGGCCTTTAATATGTGCATGGTTAGTT 1620
RVC50_St._Kitts_2016    CCAGGAGACTTTGGTTTGGTTGATAAAGAAGAATGGCCTTTAATATGTGCATGGTTAGTT 1620
CN10_Nevis_2020         CCAGGAGACTTTGGTTTGGTTGATAAAGAAGAATGGCCTTTAATATGTGCATGGTTAGTT 1620
CN14_Nevis_2020         CCAGGAGACTTTGGTTTGGTTGATAAAGAAGAATGGCCTTTAATATGTGCATGGTTAGTT 1620
CN20_Nevis_2020         CCAGGAGACTTTGGTTTGGTTGATAAAGAAGAATGGCCTTTAATATGTGCATGGTTAGTT 1620
                        ************************************************************

CPV12_USA_1978          AAACATGGTTATGAATCAACCATGGCTAACTATACACATCATTGGGGAAAAGTACCAGAA 1680
RVC50_St._Kitts_2016    AAGCATGGTTATGAATCAACCATGGCTAACTATACACATCATTGGGGAAAAGTACCAGAA 1680
CN10_Nevis_2020         AAGCATGGTTATGAATCAACCATGGCTAACTATACACATCATTGGGGAAAAGTACCAGAA 1680
CN14_Nevis_2020         AAACATGGTTATGAATCAACCATGGCTAACTATACACATCATTGGGGAAAAGTACCAGAA 1680
CN20_Nevis_2020         AAGCATGGTTATGAATCAACCATGGCTAACTATACACATCATTGGGGAAAAGTACCAGAA 1680
                        ** *********************************************************

CPV12_USA_1978          TGGGATGAAAACTGGGCGGAGCCTAAAATACAAGAAGGTATAAATTCACCAGGTTGCAAA 1740
RVC50_St._Kitts_2016    TGGGATGAAAACTGGGCGGAGCCTAAAATACAAGAAGGTATAAATTCACCAGGTTGCAAA 1740
CN10_Nevis_2020         TGGGATGAAAACTGGGCGGAGCCTAAAATACAAGAAGGTATAAATTCACCAGGTTGCAAA 1740
CN14_Nevis_2020         TGGGATGAAAACTGGGCGGAGCCTAAAATACAAGAAGGTATAAATTCACCAGGTTGCAAA 1740
CN20_Nevis_2020         TGGGATGAAAACTGGGCGGAGCCTAAAATACAAGAAGGTATAAATTCACCAGGTTGCAAA 1740
                        ************************************************************

CPV12_USA_1978          GACTTAGAGACACAAGCGGCAAGCAATCCTCAGAGTCAAGACCAAGTTCTAACTCCTCTG 1800
RVC50_St._Kitts_2016    GACTTAGAGACGCAAGCGGCAAGCAATCCTCAGAGTCAAGACCAAGTTCTAACTCCTCTG 1800
CN10_Nevis_2020         GACTTAGAGACGCAAGCGGCAAGCAATCCTCAGAGTCAAGACCAAGTTCTAACTCCTCTG 1800
CN14_Nevis_2020         GACTTAGAGACGCAAGCGGCAAGCAATCCTCAGAGTCAAGACCAAGTTCTAACTCCTCTG 1800
CN20_Nevis_2020         GACTTAGAGACGCAAGCGGCAAGCAATCCTCAGAGTCAAGACCAAGTTCTAACTCCTCTG 1800
                        *********** ************************************************

CPV12_USA_1978          ACTCCGGACGTAGTGGACCTTGCACTGGAACCGTGGAGTACTCCAGATACGCCTATTGCA 1860
RVC50_St._Kitts_2016    ACTCCGGACGTAGTGGACCTTGCACTGGAACCGTGGAGTACTCCAGATACGCCTATTGCA 1860
CN10_Nevis_2020         ACTCCGGACGTAGTGGACCTTGCACTGGAACCGTGGAGTACTCCAGATACGCCTATTGCA 1860
CN14_Nevis_2020         ACTCCGGACGTAGTGGACCTTGCACTGGAACCGTGGAGTACTCCAGATACGCCTATTGCA 1860
CN20_Nevis_2020         ACTCCGGACGTAGTGGACCTTGCACTGGAACCGTGGAGTACTCCAGATACGCCTATTGCA 1860
                        ************************************************************

CPV12_USA_1978          GAAACTGCAAATCAACAATCAAACCAACTTGGCGTTACTCACAAAGACGTGCAAGCGAGT 1920
RVC50_St._Kitts_2016    GAAACTGCAAATCAACAATCAAACCAACTTGGCGTTACTCACAAAGACGTGCAAGCGAGT 1920
CN10_Nevis_2020         GAAACTGCAAATCAACAATCAAACCAACTTGGCGTTACTCACAAAGACGTGCAAGCGAGT 1920
CN14_Nevis_2020         GAAACTGCAAATCAACAATCAAACCAACTTGGCGTTACTCACAAAGACGTGCAAGCGAGT 1920
CN20_Nevis_2020         GAAACTGCAAATCAACAATCAAACCAACTTGGCGTTACTCACAAAGACGTGCAAGCGAGT 1920
                        ************************************************************
```

```
CPV12_USA_1978           CCGACATGGTCCGAAATAGAGGCAGACCTGAGAGCCATCTTTACTTCTGAACAATTGGAA 1980
RVC50_St._Kitts_2016     CCAACGTGGTCCGAAATAGAGGCAGACCTGAGAGCCATCTTTACTTCTGAACAACTGGAA 1980
CN10_Nevis_2020          CCAACGTGGTCCGAAATAGAGGCAGACCTGAGAGCCATCTTTACTTCTGAACAACTGGAA 1980
CN14_Nevis_2020          CCAACGTGGTCCGAAATAGAGGCAGACCTGAGAGCCATCTTTACTTCTGAACAACTGGAA 1980
CN20_Nevis_2020          CCAACGTGGTCCGAAATAGAGGCAGACCTGAGAGCCATCTTTACTTCTGAACAACTGGAA 1980
                         ** ** ************************************************ *****

CPV12_USA_1978           GAAGATTTTCGAGACGACTTGGATTAAGGTACGATGGCACCTCCGGCAAAGAGAGCCAGG 2040
RVC50_St._Kitts_2016     GAAGATTTTCGAGACGACTTGGATTAAGGTACGATGGCACCTCCGGCAAAGAGAGCCAGG 2040
CN10_Nevis_2020          GAAGATTTTCGAGACGACTTGGATTAAGGTACGATGGCACCTCCGGCAAAGAGAGCCAGG 2040
CN14_Nevis_2020          GAAGATTTTCGAGACGACTTGGATTAAGGTACGATGGCACCTCCGGCAAAGAGAGCCAGG 2040
CN20_Nevis_2020          GAAGATTTTCGAGACGACTTGGATTAAGGTACGATGGCACCTCCGGCAAAGAGAGCCAGG 2040
                         ************************************************************

CPV12_USA_1978           AGAGGTAAGGGTGTGTTAGTAAAGTGGGGGGAGGGGAAAGATTTAATAACTTAACTAAGT 2100
RVC50_St._Kitts_2016     AGAGGTAAGGGTGTGTTAGTAAAGTGGGGGGAGGGGAAAGATTTAGTAACTTAACTAAGT 2100
CN10_Nevis_2020          AGAGGTAAGGGTGTGTTAGTAAAGTGGGGGGAGGGGAAAGATTTAGTAACTTAACTAAGT 2100
CN14_Nevis_2020          AGAGGTAAGGGTGTGTTAGTAAAGTGGGGGGAGGGGAAAGATTTAGTAACTTAACTAAGT 2100
CN20_Nevis_2020          AGAGGTAAGGGTGTGTTAGTAAAGTGGGGGGAGGGGAAAGATTTAGTAACTTAACTAAGT 2100
                         ********************************************* **************

CPV12_USA_1978           ATGTGTTTTTTTATAGGACTTGTGCCTCCAGGTTATAAATATCTTGGGCCTGGGAACAGT 2160
RVC50_St._Kitts_2016     ATGTGTTTTTTTATAGGACTTGTGCCTCCAGGTTATAAATATCTTGGGCCTGGAAACAGT 2160
CN10_Nevis_2020          ATGTGTTTTTTTATAGGACTTGTGCCTCCAGGTTATAAATATCTTGGGCCTGGAAACAGT 2160
CN14_Nevis_2020          ATGTGTTTTTTTATAGGACTTGTGCCTCCAGGTTATAAATATCTTGGGCCTGGAAACAGT 2160
CN20_Nevis_2020          ATGTGTTTTTTTATAGGACTTGTGCCTCCAGGTTATAAATATCTTGGGCCTGGAAACAGT 2160
                         ***************************************************** ******

CPV12_USA_1978           CTTGACCAAGGAGAACCAACTAACCCTTCTGACGCCGCTGCAAAAGAACACGACGAAGCT 2220
RVC50_St._Kitts_2016     CTTGACCAAGGAGAACCAACTAACCCTTCTGACGCCGCTGCAAAAGAACACGACGAAGCT 2220
CN10_Nevis_2020          CTTGACCAAGGAGAACCAACTAACCCTTCTGACGCCGCTGCAAAAGAACACGACGAAGCT 2220
CN14_Nevis_2020          CTTGACCAAGGAGAACCAACTAACCCTTCTGACGCCGCTGCAAAAGAACACGACGAAGCT 2220
CN20_Nevis_2020          CTTGACCAAGGAGAACCAACTAACCCTTCTGACGCCGCTGCAAAAGAACACGACGAAGCT 2220
                         ************************************************************

CPV12_USA_1978           TACGCTGCTTATCTTCGCTCTGGTAAAAACCCATACTTATATTTCTCGCCAGCAGATCAA 2280
RVC50_St._Kitts_2016     TACGCTGCTTATCTTCGCTCTGGTAAAAACCCATACTTATATTTCTCGCCAGCAGATCAA 2280
CN10_Nevis_2020          TACGCTGCTTATCTTCGCTCTGGTAAAAACCCATACTTATATTTCTCGCCAGCAGATCAA 2280
CN14_Nevis_2020          TACGCTGCTTATCTTCGCTCTGGTAAAAACCCATACTTATATTTCTCGCCAGCAGATCAA 2280
CN20_Nevis_2020          TACGCTGCTTATCTTCGCTCTGGTAAAAACCCATACTTATATTTCTCGCCAGCAGATCAA 2280
                         ************************************************************

CPV12_USA_1978           CGCTTTATAGATCAAACTAAGGACGCTAAAGATTGGGGGGGGAAAATAGGACATTATTTT 2340
RVC50_St._Kitts_2016     CGCTTTATAGATCAAACTAAGGACGCTAAAGATTGGGGGGGGAAAATAGGACATTATTTT 2340
CN10_Nevis_2020          CGCTTTATAGATCAAACTAAGGACGCTAAAGATTGGGGGGGGAAAATAGGACATTATTTT 2340
CN14_Nevis_2020          CGCTTTATAGATCAAACTAAGGACGCTAAAGATTGGGGGGGGAAAATAGGACATTATTTT 2340
CN20_Nevis_2020          CGCTTTATAGATCAAACTAAGGACGCTAAAGATTGGGGGGGGAAAATAGGACATTATTTT 2340
                         ************************************************************

CPV12_USA_1978           TTTAGAGCTAAAAAGGCAATTGCTCCAGTATTAACTGATACACCAGATCATCCATCAACA 2400
RVC50_St._Kitts_2016     TTTAGAGCTAAAAAGGCAATTGCTCCAGTATTAACTGATACACCAGATCATCCATCAACA 2400
CN10_Nevis_2020          TTTAGAGCTAAAAAGGCAATTGCTCCAGTATTAACTGATACACCAGATCATCCATCAACA 2400
CN14_Nevis_2020          TTTAGAGCTAAAAAGGCAATTGCTCCAGTATTAACTGATACACCAGATCATCCATCAACA 2400
CN20_Nevis_2020          TTTAGAGCTAAAAAGGCAATTGCTCCAGTATTAACTGATACACCAGATCATCCATCAACA 2400
                         ************************************************************
```

```
CPV12_USA_1978          TCAAGACCAACAAAACCAACTAAAAGAAGTAAACCACCACCTCATATTTTCATCAATCTT 2460
RVC50_St._Kitts_2016    TCAAGACCAACAAAACCAACTAAAAGAAGTAAACCACCACCTCATATTTTCATCAATCTT 2460
CN10_Nevis_2020         TCAAGACCAACAAAACCAACTAAAAGAAGTAAACCGCCACCTCATATTTTCATCAATCTT 2460
CN14_Nevis_2020         TCAAGACCAACAAAACCAACTAAAAGAAGTAAACCACCACCTCATATTTTCATCAATCTT 2460
CN20_Nevis_2020         TCAAGACCAACAAAACCAACTAAAAGAAGTAAACCGCCACCTCATATTTTCATCAATCTT 2460
                        *********************************** ************************

CPV12_USA_1978          GCAAAAAAAAAAAAAGCCGGTGCAGGACAAGTAAAAAGAGACAATCTTGCACCAATGAGT 2520
RVC50_St._Kitts_2016    GCAAAAAAAAAAAAAGCCGGTGCAGGACAAGTAAAAAGAGACAATCTTGCACCAATGAGT 2520
CN10_Nevis_2020         GCAAAAAAAAAAAAAGCCGGTGCAGGACAAGTAAAAAGAGACAATCTTGCACCAATGAGT 2520
CN14_Nevis_2020         GCAAAAAAAAAAAAAGCCGGTGCAGGACAAGTAAAAAGAGACAATCTTGCACCAATGAGT 2520
CN20_Nevis_2020         GCAAAAAAAAAAAAAGCCGGTGCAGGACAAGTAAAAAGAGACAATCTTGCACCAATGAGT 2520
                        ************************************************************

CPV12_USA_1978          GATGGAGCAGTTCAACCAGACGGTGGTCAACCTGCTGTCAGAAATGAAAGAGCTACAGGA 2580
RVC50_St._Kitts_2016    GATGGAGCAGTTCAACCAGACGGTGGTCAACCTGCTGTCAGAAATGAAAGAGCAACAGGA 2580
CN10_Nevis_2020         GATGGAGCAGTTCAACCAGACGGTGGTCAACCTGCTGTCAGAAATGAAAGAGCAACAGGA 2580
CN14_Nevis_2020         GATGGAGCAGTTCAACCAGACGGTGGTCAACCTGCTGTCAGAAATGAAAGAGCAACAGGA 2580
CN20_Nevis_2020         GATGGAGCAGTTCAACCAGACGGTGGTCAACCTGCTGTCAGAAATGAAAGAGCAACAGGA 2580
                        ***************************************************** ******

CPV12_USA_1978          TCTGGGAACGGGTCTGGAGGCGGGGGTGGTGGTGGTTCTGGGGGTGTGGGGATTTCTACG 2640
RVC50_St._Kitts_2016    TCTGGGAACGGGTCTGGAGGCGGGGGTGGTGGTGGTTCTGGGGGTGTGGGGATTTCTACG 2640
CN10_Nevis_2020         TCTGGGAACGGGTCTGGAGGCGGGGGTGGTGGTGGTTCTGGGGGTGTGGGGATTTCTACG 2640
CN14_Nevis_2020         TCTGGGAACGGGTCTGGAGGCGGGGGTGGTGGTGGTTCTGGGGGTGTGGGGATTTCTACG 2640
CN20_Nevis_2020         TCTGGGAACGGGTCTGGAGGCGGGGGTGGTGGTGGTTCTGGGGGTGTGGGGATTTCTACG 2640
                        ************************************************************

CPV12_USA_1978          GGTACTTTCAATAATCAGACGGAATTTAAATTTTTGGAAAACGGATGGGTGGAAATCACA 2700
RVC50_St._Kitts_2016    GGTACTTTCAATAATCAGACGGAATTTAAATTTTTGGAAAACGGATGGGTGGAAATCACA 2700
CN10_Nevis_2020         GGTACTTTCAATAATCAGACGGAATTTAAATTTTTGGAAAACGGATGGGTGGAAATCACA 2700
CN14_Nevis_2020         GGTACTTTCAATAATCAGACGGAATTTAAATTTTTGGAAAACGGATGGGTGGAAATCACA 2700
CN20_Nevis_2020         GGTACTTTCAATAATCAGACGGAATTTAAATTTTTGGAAAACGGATGGGTGGAAATCACA 2700
                        ************************************************************

CPV12_USA_1978          GCAAACTCAAGCAGACTTGTACATTTAAATATGCCAGAAAGTGAAAATTATAGAAGAGTG 2760
RVC50_St._Kitts_2016    GCAAACTCAAGCAGACTTGTACATTTAAATATGCCAGAAAGTGAAAATTATAGAAGAGTG 2760
CN10_Nevis_2020         GCAAACTCAAGCAGACTTGTACATTTAAATATGCCAGAAAGTGAAAATTATAGAAGAGTG 2760
CN14_Nevis_2020         GCAAACTCAAGCAGACTTGTACATTTAAATATGCCAGAAAGTGAAAATTATAGAAGAGTG 2760
CN20_Nevis_2020         GCAAACTCAAGCAGACTTGTACATTTAAATATGCCAGAAAGTGAAAATTATAGAAGAGTG 2760
                        ************************************************************

CPV12_USA_1978          GTTGTAAATAATATGGATAAAACTGCAGTTAACGGAAACATGGCTTTAGATGATATTCAT 2820
RVC50_St._Kitts_2016    GTTGTAAATAATTTGGATAAAACTGCAGTTAACGGAAACATGGCTTTAGATGATACTCAT 2820
CN10_Nevis_2020         GTTGTAAATAATTTGGATAAAACTGCAGTTAACGGAAACATGGCTTTAGATGATACTCAT 2820
CN14_Nevis_2020         GTTGTAAATAATTTGGATAAAACTGCAGTTAACGGAAACATGGCTTTAGATGATACTCAT 2820
CN20_Nevis_2020         GTTGTAAATAATTTGGATAAAACTGCAGTTAACGGAAACATGGCTTTAGATGATACTCAT 2820
                        ************ ****************************************** ****

CPV12_USA_1978          GCACAAATTGTAACACCTTGGTCATTGGTTGATGCAAATGCTTGGGGAGTTTGGTTTAAT 2880
RVC50_St._Kitts_2016    GCACAAATTGTAACACCTTGGTCATTGGTTGATGCAAATGCTTGGGGAGTTTGGTTTAAT 2880
CN10_Nevis_2020         GCACAAATTGTAACACCTTGGTCATTGGTTGATGCAAATGCTTGGGGAGTTTGGTTTAAT 2880
CN14_Nevis_2020         GCACAAATTGTAACACCTTGGTCATTGGTTGATGCAAATGCTTGGGGAGTTTGGTTTAAT 2880
CN20_Nevis_2020         GCACAAATTGTAACACCTTGGTCATTGGTTGATGCAAATGCTTGGGGAGTTTGGTTTAAT 2880
                        ************************************************************
```

```
CPV12_USA_1978          CCAGGAGATTGGCAACTAATTGTTAATACTATGAGTGAGTTGCATTTAGTTAGTTTTGAA 2940
RVC50_St._Kitts_2016    CCAGGAGATTGGCAACTAATTGTTAATACTATGAGTGAGTTACATTTAGTTAGTTTTGAG 2940
CN10_Nevis_2020         CCAGGAGATTGGCAACTAATTGTTAATACTATGAGTGAGTTACATTTAGTTAGTTTTGAG 2940
CN14_Nevis_2020         CCAGGAGATTGGCAACTAATTGTTAATACTATGAGTGAGTTACATTTAGTTAGTTTTGAG 2940
CN20_Nevis_2020         CCAGGAGATTGGCAACTAATTGTTAATACTATGAGTGAGTTACATTTAGTTAGTTTTGAG 2940
                        ***************************************** ***************** 

CPV12_USA_1978          CAAGAAATTTTTAATGTTGTTTTAAAGACTGTTTCAGAATCTGCTACTCAGCCACCAACT 3000
RVC50_St._Kitts_2016    CAAGAAATTTTTAATGTTGTTTTAAAGACTGTTTCAGAATCTGCTACTCAGCCACCAACT 3000
CN10_Nevis_2020         CAAGAAATTTTTAATGTTGTTTTAAAGACTGTTTCAGAATCTGCTACTCAGCCACCAACT 3000
CN14_Nevis_2020         CAAGAAATTTTTAATGTTGTTTTAAAGACTGTTTCAGAATCTGCTACTCAGCCACCAACT 3000
CN20_Nevis_2020         CAAGAAATTTTTAATGTTGTTTTAAAGACTGTTTCAGAATCTGCTACTCAGCCACCAACT 3000
                        ************************************************************

CPV12_USA_1978          AAAGTCTATAATAATGATTTAACTGCATCATTGATGGTTGCATTAGATAGTAATAATACT 3060
RVC50_St._Kitts_2016    AAAGTTTATAATAATGATTTAACTGCATCATTGATGGTTGCATTAGATAGTAATAATACT 3060
CN10_Nevis_2020         AAAGTTTATAATAATGATTTAACTGCATCATTGATGGTGGCATTAGATAGTAATAATACT 3060
CN14_Nevis_2020         AAAGTTTATAATAATGATTTAACTGCATCATTGATGGTTGCATTAGATAGTAATAATACT 3060
CN20_Nevis_2020         AAAGTTTATAATAATGATTTAACTGCATCATTGATGGTTGCATTAGATAGTAATAATACT 3060
                        ***** ********************************* *********************

CPV12_USA_1978          ATGCCATTTACTCCAGCAGCTATGAGATCTGAGACATTGGGTTTTTATCCATGGAAACCA 3120
RVC50_St._Kitts_2016    ATGCCATTTACTCCAGCAGCTATGAGATCTGAGACATTGGGTTTTTATCCATGGAAACCA 3120
CN10_Nevis_2020         ATGCCATTTACTCCAGCAGCTATGAGATCTGAGACATTGGGTTTTTATCCATGGAAGCCA 3120
CN14_Nevis_2020         ATGCCATTTACTCCAGCAGCTATGAGATCTGAGACATTGGGTTTTTATCCATGGAAACCA 3120
CN20_Nevis_2020         ATGCCATTTACTCCAGCAGCTATGAGATCTGAGACATTGGGTTTTTATCCATGGAAACCA 3120
                        ******************************************************** ***

CPV12_USA_1978          ACCATACCAACTCCATGGAGATATTATTTTCAATGGGATAGAACATTAATACCATCTCAT 3180
RVC50_St._Kitts_2016    ACCATACCAACTCCATGGAGATATTATTTTCAATGGGATAGAACATTAATACCATCTCAT 3180
CN10_Nevis_2020         ACCATACCAACTCCATGGAGATATTATTTTCAATGGGATAGAACATTAATACCATCTCAT 3180
CN14_Nevis_2020         ACCATACCAACTCCATGGAGATATTATTTTCAATGGGATAGAACATTAATACCATCTCAT 3180
CN20_Nevis_2020         ACCATACCAACTCCATGGAGATATTATTTTCAATGGGATAGAACATTAATACCATCTCAT 3180
                        ************************************************************

CPV12_USA_1978          ACTGGAACTAGTGGCACACCAACAAATATATACCATGGTACAGATCCAGATGATGTTCAA 3240
RVC50_St._Kitts_2016    ACTGGAACTAGTGGCACACCAACAAATATATACCATGGTACAGATCCAGATGATGTTCAA 3240
CN10_Nevis_2020         ACTGGAACTAGTGGCACACCAACAAATATATACCATGGTACAGATCCAGATGATGTTCAA 3240
CN14_Nevis_2020         ACTGGAACTAGTGGCACACCAACAAATATATACCATGGTACAGATCCAGATGATGTTCAA 3240
CN20_Nevis_2020         ACTGGAACTAGTGGCACACCAACAAATATATACCATGGTACAGATCCAGATGATGTTCAA 3240
                        ************************************************************

CPV12_USA_1978          TTTTATACTATTGAAAATTCTGTGCCAGTACACTTACTAAGAACAGGTGATGAATTTGCT 3300
RVC50_St._Kitts_2016    TTTTATACTATTGAAAATTCTGTGCCAGTACACTTACTAAGAACAGGTGATGAATTTGCT 3300
CN10_Nevis_2020         TTTTATACTATTGAAAATTCTGTGCCAGTACACTTACTAAGAACAGGTGATGAATTTGCT 3300
CN14_Nevis_2020         TTTTATACTATTGAAAATTCTGTGCCAGTACACTTACTAAGAACAGGTGATGAATTTGCT 3300
CN20_Nevis_2020         TTTTATACTATTGAAAATTCTGTGCCAGTACACTTACTAAGAACAGGTGATGAATTTACT 3300
                        ********************************************************* **

CPV12_USA_1978          ACAGGAACATTTTTTTTTGATTGTAAACCATGTAGACTAACACATACATGGCAAACAAAT 3360
RVC50_St._Kitts_2016    ACAGGAACATTTTTTTTTGATTGTAAACCATGTAGACTAACACATACATGGCAAACAAAT 3360
CN10_Nevis_2020         ACAGGAACATTTTTTTTTGATTGTAAACCATGTAGACTAACACATACATGGCAAACAAAT 3360
CN14_Nevis_2020         ACAGGAACATTTTTTTTTGATTGTAAACCATGTAGACTAACACATACATGGCAAACAAAT 3360
CN20_Nevis_2020         ACAGGAACATTTTTTTTTGATTGTAAACCATGTAGACTAACACATACATGGCAAACAAAT 3360
                        ************************************************************
```

```
CPV12_USA_1978            AGAGCATTGGGCTTACCACCATTTCTAAATTCTTTGCCTCAATCTGAAGGAGCTACTAAC 3420
RVC50_St._Kitts_2016      AGAGCATTGGGCTTACCACCATTTCTAAATTCTTTGCCTCAAGCTGAAGGAGGTACTAAC 3420
CN10_Nevis_2020           AGAGCATTGGGCTTACCACCATTTCTAAATTCTTTGCCTCAAGCTGAAGGAGGTACTAAC 3420
CN14_Nevis_2020           AGAGCATTGGGCTTACCACCATTTCTAAATTCTTTGCCTCAAGCTGAAGGAGGTACTAAC 3420
CN20_Nevis_2020           AGAGCATTGGGCTTACCACCATTTCTAAATTCTTTGCCTCAAGCTGAAGGAGGTACTAAC 3420
                          ****************************************** ********* *******

CPV12_USA_1978            TTTGGTGATATAGGAGTTCAACAAGATAAAAGACGTGGTGTAACTCAAATGGGAAATACA 3480
RVC50_St._Kitts_2016      TTTGGTTATATAGGAGTTCAACAAGATAAAAGACGTGGTGTAACTCAAATGGGAAATACA 3480
CN10_Nevis_2020           TTTGGTTATATAGGAGTTCAACAAGATAAAAGACGTGGTGTAACTCAAATGGGAAATACA 3480
CN14_Nevis_2020           TTTGGTTATATAGGAGTTCAACAAGATAAAAGACGTGGTGTAACTCAAATGGGAAATACA 3480
CN20_Nevis_2020           TTTGGTTATATAGGAGTTCAACAAGATAAAAGACGTGGTGTAACTCAAATGGGAAATACA 3480
                          ****** *****************************************************

CPV12_USA_1978            AACTATATTACTGAAGCTACTATTATGAGACCAGCTGAGGTTGGTTATAGTGCACCATAT 3540
RVC50_St._Kitts_2016      AACTATATTACTGAAGCTACTATTATGAGACCAGCTGAGGTTGGTTATAGTGCACCATAT 3540
CN10_Nevis_2020           AACTATATTACTGAAGCTACTATTATGAGACCAGCTGAGGTTGGTTATAGTGCACCATAT 3540
CN14_Nevis_2020           AACTATATTACTGAAGCTACTATTATGAGACCAGCTGAGGTTGGTTATAGTGCACCATAT 3540
CN20_Nevis_2020           AACTATATTACTGAAGCTACTATTATGAGACCAGCTGAGGTTGGTTATAGTGCACCATAT 3540
                          ************************************************************

CPV12_USA_1978            TATTCTTTTGAGGCGTCTACACAAGGGCCATTTAAAACACCTATTGCAGCAGGACGGGGG 3600
RVC50_St._Kitts_2016      TATTCTTTTGAGGCGTCTACACAAGGGCCATTTAAAACACCCATTGCAGCAGGACGGGGG 3600
CN10_Nevis_2020           TATTCTTTTGAGGCGTCTACACAAGGGCCATTTAAAACACCCATTGCAGCAGGACGGGGG 3600
CN14_Nevis_2020           TATTCTTTTGAGGCGTCTACACAAGGGCCATTTAAAACACCCATTGCAGCAGGACGGGGG 3600
CN20_Nevis_2020           TATTCTTTTGAGGCGTCTACACAAGGGCCATTTAAAACACCCATTGCAGCAGGACGGGGG 3600
                          ***************************************** ******************

CPV12_USA_1978            GGAGCGCAAACAGATGAAAATCAAGCAGCAGATGGTAATCCAAGATATGCATTTGGTAGA 3660
RVC50_St._Kitts_2016      GGAGCGCAAACAGATGAAAATCAAGCAGCAGATGGTGATCCAAGATATGCATTTGGTAGA 3660
CN10_Nevis_2020           GGAGCGCAAACAGATGAAAATCAAGCAGCAGATGGTGATCCAAGATATGCATTTGGTAGA 3660
CN14_Nevis_2020           GGAGCGCAAACAGATGAAAATCAAGCAGCAAATGGTGATCCAAGATATGCATTTGGTAGA 3660
CN20_Nevis_2020           GGAGCGCAAACAGATGAAAATCAAGCAGCAGATGGTGATCCAAGATATGCATTTGGTAGA 3660
                          ****************************** ***** ***********************

CPV12_USA_1978            CAACATGGTCAAAAAACTACCACAACAGGAGAAACACCTGAGAGATTTACATATATAGCA 3720
RVC50_St._Kitts_2016      CAACATGGTCAAAAAACTACCACAACAGGAGAAACACCTGAGAGATTTACATATATAGCA 3720
CN10_Nevis_2020           CAACATGGTCAAAAAACTACCACAACAGGAGAAACACCTGAGAGATTTACATATATAGCA 3720
CN14_Nevis_2020           CAACATGGTCAAAAAACTACCACAACAGGAGAAACACCTGAAAGATTTACATATATAGCA 3720
CN20_Nevis_2020           CAACATGGTCAAAAAACTACCACAACAGGAGAAACACCTGAGAGATTTACATATATAGCA 3720
                          ***************************************** ******************

CPV12_USA_1978            CATCAAGATACAGGAAGATATCCAGAAGGAGATTGGATTCAAAATATTAACTTTAACCTT 3780
RVC50_St._Kitts_2016      CATCAAGATACAGGAAGATATCCAGAAGGAGATTGGATTCAAAATATTAACTTTAACCTT 3780
CN10_Nevis_2020           CATCAAGATACAGGAAGATATCCAGAAGGAGATTGGATTCAAAATATTAACTTTAACCTT 3780
CN14_Nevis_2020           CATCAAGATACAGGAAGATATCCAGAAGGAGATTGGATTCAAAATATTAACTTTAACCTT 3780
CN20_Nevis_2020           CATCAAGATACAGGAAGATATCCAGAAGGAGATTGGATTCAAAATATTAACTTTAACCTT 3780
                          ************************************************************

CPV12_USA_1978            CCTGTAACAAATGATAATGTATTGCTACCAACAGATCCAATTGGAGGTAAAACAGGAATT 3840
RVC50_St._Kitts_2016      CCTGTAACAAATGATAATGTATTGCTACCAACAGATCCAATTGGAGGTAAAACAGGAATT 3840
CN10_Nevis_2020           CCTGTAACAAATGATAATGTATTGCTACCAACAGATCCAATTGGAGGTAAAACAGGAATT 3840
CN14_Nevis_2020           CCTGTAACAAATGATAATGTATTGCTACCAACAGATCCAATTGGAGGTAAAACAGGAATT 3840
CN20_Nevis_2020           CCTGTAACAAATGATAATGTATTGCTACCAACAGATCCAATTGGAGGTAAAACAGGAATT 3840
                          ************************************************************
```

```
CPV12_USA_1978            AACTATACTAATATATTTAATACTTATGGTCCTTTAACTGCATTAAATAATGTACCACCA 3900
RVC50_St._Kitts_2016      AACTATACTAATATATTTAATACTTATGGTCCTTTAACTGCATTAAATAATGTGCCACCA 3900
CN10_Nevis_2020           AACTATACTAATATATTTAATACTTATGGTCCTTTAACTGCATTAAATAATGTGCCACCA 3900
CN14_Nevis_2020           AACTATACTAATATATTTAATACTTATGGTCCTTTAACTGCATTAAATAATGTGCCACCA 3900
CN20_Nevis_2020           AACTATACTAATATATTTAATACTTATGGTCCTTTAACTGCATTAAATAATGTGCCACCA 3900
                          ***************************************************** ******

CPV12_USA_1978            GTTTATCCAAATGGTCAAATTTGGGATAAAGAATTTGATACTGACTTAAAACCAAGACTT 3960
RVC50_St._Kitts_2016      GTTTATCCAAATGGTCAAATTTGGGATAAAGAATTTGATACTGACTTAAAACCAAGACTT 3960
CN10_Nevis_2020           GTTTATCCAAATGGTCAAATTTGGGATAAAGAATTTGATACTGACTTAAAACCAAGACTT 3960
CN14_Nevis_2020           GTTTATCCAAATGGTCAAATTTGGGATAAAGAATTTGATACTGACTTAAAACCAAGACTT 3960
CN20_Nevis_2020           GTTTATCCAAATGGTCAAATTTGGGATAAAGAATTTGATACTGACTTAAAACCAAGACTT 3960
                          ************************************************************

CPV12_USA_1978            CATGTAAATGCACCATTTGTTTGTCAAAATAATTGTCCTGGTCAATTATTTGTAAAAGTT 4020
RVC50_St._Kitts_2016      CATGTAAATGCACCATTTGTTTGTCAAAATAATTGTCCTGGTCAATTATTTGTAAAGGTT 4020
CN10_Nevis_2020           CATGTAAATGCACCATTTGTTTGTCAAAATAATTGTCCTGGTCAATTATTTGTAAAGGTT 4020
CN14_Nevis_2020           CATGTAAATGCACCATTTGTTTGTCAAAATAATTGTCCTGGTCAATTATTTGTAAAGGTT 4020
CN20_Nevis_2020           CATGTAAATGCACCATTTGTTTGTCAAAATAATTGTCCTGGTCAATTATTTGTAAAGGTT 4020
                          ******************************************************** ***

CPV12_USA_1978            GCGCCTAATTTAACAAATGAATATGATCCTGATGCATCTGCTAATATGTCAAGAATTGTA 4080
RVC50_St._Kitts_2016      GCGCCTAATCTAACAAATGAATATGATCCTGATGCATCTGCTAATATGTCAAGAATTGTA 4080
CN10_Nevis_2020           GCGCCTAATCTAACAAATGAATATGATCCTGATGCATCTGCTAATATGTCAAGAATTGTA 4080
CN14_Nevis_2020           GCGCCTAATCTAACAAATGAATATGATCCTGATGCATCTGCTAATATGTCAAGAATTGTA 4080
CN20_Nevis_2020           GCGCCTAATCTAACAAATGAATATGATCCTGATGCATCTGCTAATATGTCAAGAATTGTA 4080
                          ********* **************************************************

CPV12_USA_1978            ACTTACTCAGATTTTTGGTGGAAAGGTAAATTAGTATTTAAAGCTAAACTAAGAGCCTCT 4140
RVC50_St._Kitts_2016      ACTTACTCAGATTTTTGGTGGAAAGGTAAATTAGTATTTAAAGCTAAACTAAGAGCCTCT 4140
CN10_Nevis_2020           ACTTACTCAGATTTTTGGTGGAAAGGTAAATTAGTATTTAAAGCTAAACTAAGAGCCTCT 4140
CN14_Nevis_2020           ACTTACTCAGATTTTTGGTGGAAAGGTAAATTAGTATTTAAAGCTAAACTAAGAGCCTCT 4140
CN20_Nevis_2020           ACTTACTCAGATTTTTGGTGGAAAGGTAAATTAGTATTTAAAGCTAAACTAAGAGCCTCT 4140
                          ************************************************************

CPV12_USA_1978            CATACTTGGAATCCAATTCAACAAATGAGTATTAATGTAGATAACCAATTTAACTATGTA 4200
RVC50_St._Kitts_2016      CATACTTGGAATCCAATTCAACAAATGAGTATTAATGTAGATAACCAATTTAACTATGTA 4200
CN10_Nevis_2020           CATACTTGGAATCCAATTCAACAAATGAGTATTAATGTAGATAACCAATTTAACTATGTA 4200
CN14_Nevis_2020           CATACTTGGAATCCAATTCAACAAATGAGTATTAATGTAGATAACCAATTTAACTATGTA 4200
CN20_Nevis_2020           CATACTTGGAATCCAATTCAACAAATGAGTATTAATGTAGATAACCAATTTAACTATGTA 4200
                          ************************************************************

CPV12_USA_1978            CCAAGTAATATTGGAGGTATGAAAATTGTATATGAAAAATCTCAACTAGCACCTAGAAAA 4260
RVC50_St._Kitts_2016      CCAAGTAATATTGGAGGTATGAAAATTGTATATGAAAAATCTCAACTAGCACCTAGAAAA 4260
CN10_Nevis_2020           CCAAGTAATATTGGAGGTATGAAAATTGTATATGAAAAATCTCAACTAGCACCTAGAAAA 4260
CN14_Nevis_2020           CCAAGTAATATTGGAGGTATGAAAATTGTATATGAAAAATCTCAACTAGCACCTAGAAAA 4260
CN20_Nevis_2020           CCAAGTAATATTGGAGGTATGAAAATTGTATATGAAAAATCTCAACTAGCACCTAGAAAA 4260
                          ************************************************************

CPV12_USA_1978            TTATATTAA 4269
RVC50_St._Kitts_2016      TTATATTAA 4269
CN10_Nevis_2020           TTATATTAA 4269
CN14_Nevis_2020           TTATATTAA 4269
CN20_Nevis_2020           TTATATTAA 4269
                          *********
```
