# Supplementary material for: Molecular Investigation of Canine Parvovirus-2 (CPV-2) Outbreak in Nevis Island: Analysis of the Nearly Complete Genomes of CPV-2 Strains from the Caribbean Region
Source: Viruses. 2021 Jun 6;13(6):1083. doi: 10.3390/v13061083 (PMC8227521; doi:10.3390/v13061083)
Supplement: Supplementary file 1 [file viruses-13-01083-s001.zip › Supplementary materials_R1/Supplementary material S6.pdf]

Supplementary material S6. Multiple alignment of the putative NS1 (A), NS2 (B), VP1 (C) and VP2 (D) proteins of canine parvovirus-2 (CPV-2) strain CPV12 (GenBank accession number MN451655, representing the earliest CPV-2 strains from the late 1970s) and CPV-2 strains detected on St. Kitts in 2016 (strain RVC50) and Nevis in 2020 (strains CN10, CN14 and CN20). A '*' denotes an identical amino acid (aa) residue. Numbers to the right indicate the positions of the aa for respective CPV-2 strains.

(A) NS1

```
CPV12_NS1       MSGNQYTEEVMEGVNWLKKHAENEAFSFVFKCDNVQLNGKDVRWNNYTKPIQNEELTSLI 60
CN10_NS1        MSGNQYTEEVMEGVNWLKKHAENEAFSFVFKCDNVQLNGKDVRWNNYTKPIQNEELTSLI 60
CN14_NS1        MSGNQYTEEVMEGVNWLKKHAENEAFSFVFKCDNVQLNGKDVRWNNYTKPIQNEELTSLI 60
CN20_NS1        MSGNQYTEEVMEGVNWLKKHAENEAFSFVFKCDNVQLNGKDVRWNNYTKPIQNEELTSLI 60
RVC50_NS1       MSGNQYTEEVMEGVNWLKKHAENEAFSFVFKCDNVQLNGKDVRWNNYTKPIQNEELTSLI 60
                ************************************************************

CPV12_NS1       RGAQTAMDQTEEEEMDWESEVDSLAKKQVQTFDALIKKCLFEVFVSKNIEPNECVWFIQH 120
CN10_NS1        RGAQTAMDQTEEEEMDWESEVDSLAKKQVQTFDALIKKCLFEVFVSKNIEPNECVWFIQH 120
CN14_NS1        RGAQTAMDQTEEEEMDWESEVDSLAKKQVQTFDALIKKCLFEVFVSKNIEPNECVWFIQH 120
CN20_NS1        RGAQTAMDQTEEEEMDWESEVDSLAKKQVQTFDALIKKCLFEVFVSKNIEPNECVWFIQH 120
RVC50_NS1       RGAQTAMDQTEEEEMDWESEVDSLAKKQVQTFDALIKKCLFEVFVSKNIEPNECVWFIQH 120
                ************************************************************

CPV12_NS1       EWGKDQGWHCHVLLHSKNLQQATGKWLRRQMNMYWSRWLVTLCSVNLTPTEKIKLREIAE 180
CN10_NS1        EWGKDQGWHCHVLLHSKNLQQATGKWLRRQMNMYWSRWLVTLCSVNLTPTEKIKLREIAE 180
CN14_NS1        EWGKDQGWHCHVLLHSKNLQQATGKWLRRQMNMYWSRWLVTLCSVNLTPTEKIKLREIAE 180
CN20_NS1        EWGKDQGWHCHVLLHSKNLQQATGKWLRRQMNMYWSRWLVTLCSVNLTPTEKIKLREIAE 180
RVC50_NS1       EWGKDQGWHCHVLLHSKNLQQATGKWLRRQMNMYWSRWLVTLCSVNLTPTEKIKLREIAE 180
                ************************************************************

CPV12_NS1       DSEWVTILTYRHKQTKKDYVKMVHFGNMIAYYFLTKKKIVHMTKESGYFLSTDSGWKFNF 240
CN10_NS1        DSEWVTILTYRHKQTKKDYVKMVHFGNMIAYYFLTKKKIVHMTKESGYFLSTDSGWKFNF 240
CN14_NS1        DSEWVTILTYRHKQTKKDYVKMVHFGNMIAYYFLTKKKIVHMTKESGYFLSTDSGWKFNF 240
CN20_NS1        DSEWVTILTYRHKQTKKDYVKMVHFGNMIAYYFLTKKKIVHMTKESGYFLSTDSGWKFNF 240
RVC50_NS1       DSEWVTILTYRHKQTKKDYVKMVHFGNMIAYYFLTKKKIVHMTKESGYFLSTDSGWKFNF 240
                ************************************************************

CPV12_NS1       MKYQDRQIVSTLYTEQMKPETVETTVTTAQETKRGRIQTKKEVSIKCTLRDLVSKRVTSP 300
CN10_NS1        MKYQDRQIVSTLYTEQMKPETVETTVTTAQETKRGRIQTKKEVSIKCTLRDLVSKRVTSP 300
CN14_NS1        MKYQDRQIVSTLYTEQMKPETVETTVTTAQETKRGRIQTKKEVSIKCTLRDLVSKRVTSP 300
CN20_NS1        MKYQDRQIVSTLYTEQMKPETVETTVTTAQETKRGRIQTKKEVSIKCTLRDLVSKRVTSP 300
RVC50_NS1       MKYQDRQIVSTLYTEQMKPETVETTVTTAQETKRGRIQTKKEVSIKCTLRDLVSKRVTSP 300
                ************************************************************

CPV12_NS1       EDWMMLQPDSYIEMMAQPGGENLLKNTLEICTLTLARTKTAFELILEKADNTKLTNFDLA 360
CN10_NS1        EDWMMLQPDSYIEMMAQPGGENLLKNTLEICTLTLARTKTAFELILEKADNTKLTNFDLA 360
CN14_NS1        EDWMMLQPDSYIEMMAQPGGENLLKNTLEICTLTLARTKTAFELILEKADNTKLTNFDLA 360
CN20_NS1        EDWMMLQPDSYIEMMAQPGGENLLKNTLEICTLTLARTKTAFELILEKADNTKLTNFDLA 360
RVC50_NS1       EDWMMLQPDSYIEMMAQPGGENLLKNTLEICTLTLARTKTAFELILEKADNTKLTNFDLA 360
                ************************************************************

CPV12 _NS1      NSRTCQIFRMHGWNWIKVCHAIACVLNRQGGKRNTVLFHGPASTGKSIIAQAIAQAVGNV 420
CN10_NS1        NSRTCQIFRMHGWNWIKVCHAIACVLNRQGGKRNTVLFHGPASTGKSIIAQAIAQAVGNV 420
CN14_NS1        NSRTCQIFRMHGWNWIKVCHAIACVLNRQGGKRNTVLFHGPASTGKSIIAQAIAQAVGNV 420
CN20_NS1        NSRTCQIFRMHGWNWIKVCHAIACVLNRQGGKRNTVLFHGPASTGKSIIAQAIAQAVGNV 420
RVC50_NS1       NSRTCQIFRMHGWNWIKVCHAIACVLNRQGGKRNTVLFHGPASTGKSIIAQAIAQAVGNV 420
                ************************************************************

CPV12_NS1       GCYNAANVNFPFNDCTNKNLIWIEEAGNFGQQVNQFKAICSGQTIRIDQKGKGSKQIEPT 480
CN10_NS1        GCYNAANVNFPFNDCTNKNLIWIEEAGNFGQQVNQFKAICSGQTIRIDQKGKGSKQIEPT 480
CN14_NS1        GCYNAANVNFPFNDCTNKNLIWIEEAGNFGQQVNQFKAICSGQTIRIDQKGKGSKQIEPT 480
CN20_NS1        GCYNAANVNFPFNDCTNKNLIWIEEAGNFGQQVNQFKAICSGQTIRIDQKGKGSKQIEPT 480
RVC50_NS1       GCYNAANVNFPFNDCTNKNLIWIEEAGNFGQQVNQFKAICSGQTIRIDQKGKGSKQIEPT 480
                ************************************************************

CPV12_NS1       PVIMTTNENITIVRIGCEERPEHTQPIRDRMLNIKLVCKLPGDFGLVDKEEWPLICAWLV 540
CN10_NS1        PVIMTTNENITIVRIGCEERPEHTQPIRDRMLNIKLVCKLPGDFGLVDKEEWPLICAWLV 540
CN14_NS1        PVIMTTNENITIVRIGCEERPEHTQPIRDRMLNIKLVCKLPGDFGLVDKEEWPLICAWLV 540
CN20_NS1        PVIMTTNENITIVRIGCEERPEHTQPIRDRMLNIKLVCKLPGDFGLVDKEEWPLICAWLV 540
RVC50_NS1       PVIMTTNENITIVRIGCEERPEHTQPIRDRMLNIKLVCKLPGDFGLVDKEEWPLICAWLV 540
                ************************************************************
```

```
CPV12_NS1       KHGYESTMANYTHHWGKVPEWDENWAEPKIQEGINSPGCKDLETQAASNPQSQDQVLTPL 600
CN10_NS1        KHGYESTMANYTHHWGKVPEWDENWAEPKIQEGINSPGCKDLETQAASNPQSQDQVLTPL 600
CN14_NS1        KHGYESTMANYTHHWGKVPEWDENWAEPKIQEGINSPGCKDLETQAASNPQSQDQVLTPL 600
CN20_NS1        KHGYESTMANYTHHWGKVPEWDENWAEPKIQEGINSPGCKDLETQAASNPQSQDQVLTPL 600
RVC50_NS1       KHGYESTMANYTHHWGKVPEWDENWAEPKIQEGINSPGCKDLETQAASNPQSQDQVLTPL 600
                ************************************************************

CPV12_NS1       TPDVVDLALEPWSTPDTPIAETANQQSNQLGVTHKDVQASPTWSEIEADLRAIFTSEQLE 660
CN10_NS1        TPDVVDLALEPWSTPDTPIAETANQQSNQLGVTHKDVQASPTWSEIEADLRAIFTSEQLE 660
CN14_NS1        TPDVVDLALEPWSTPDTPIAETANQQSNQLGVTHKDVQASPTWSEIEADLRAIFTSEQLE 660
CN20_NS1        TPDVVDLALEPWSTPDTPIAETANQQSNQLGVTHKDVQASPTWSEIEADLRAIFTSEQLE 660
RVC50_NS1       TPDVVDLALEPWSTPDTPIAETANQQSNQLGVTHKDVQASPTWSEIEADLRAIFTSEQLE 660
                ************************************************************

CPV12_NS1       EDFRDDLD 668
CN10_NS1        EDFRDDLD 668
CN14_NS1        EDFRDDLD 668
CN20_NS1        EDFRDDLD 668
RVC50_NS1       EDFRDDLD 668
                ********
```

(B) NS2

```
CPV12_NS2      MSGNQYTEEVMEGVNWLKKHAENEAFSFVFKCDNVQLNGKDVRWNNYTKPIQNEELTSLI 60
CN10_NS2       MSGNQYTEEVMEGVNWLKKHAENEAFSFVFKCDNVQLNGKDVRWNNYTKPIQNEELTSLI 60
CN14_NS2       MSGNQYTEEVMEGVNWLKKHAENEAFSFVFKCDNVQLNGKDVRWNNYTKPIQNEELTSLI 60
CN20_NS2       MSGNQYTEEVMEGVNWLKKHAENEAFSFVFKCDNVQLNGKDVRWNNYTKPIQNEELTSLI 60
RVC50_NS2      MSGNQYTEEVMEGVNWLKKHAENEAFSFVFKCDNVQLNGKDVRWNNYTKPIQNEELTSLI 60
               ************************************************************

CPV12_NS2      RGAQTAMDQTEEEEMDWESEVDSLAKKLQRLRDTSGKQSSESRPSSNSSDSGRSGPCTGT 120
CN10_NS2       RGAQTAMDQTEEEEMDWESEVDSLAKKLQRLRDASGKQSSESRPSSNSSDSGRSGPCTGT 120
CN14_NS2       RGAQTAMDQTEEEEMDWESEVDSLAKKLQRLRDASGKQSSESRPSSNSSDSGRSGPCTGT 120
CN20_NS2       RGAQTAMDQTEEEEMDWESEVDSLAKKLQRLRDASGKQSSESRPSSNSSDSGRSGPCTGT 120
RVC50_NS2      RGAQTAMDQTEEEEMDWESEVDSLAKKLQRLRDASGKQSSESRPSSNSSDSGRSGPCTGT 120
               *********************************:**************************

CPV12_NS2      VEYSRYAYCRNCKSTIKPTWRYSQRRASESDMVRNRGRPESHLYF 165
CN10_NS2       VEYSRYAYCRNCKSTIKPTWRYSQRRASESNVVRNRGRPESHLYF 165
CN14_NS2       VEYSRYAYCRNCKSTIKPTWRYSQRRASESNVVRNRGRPESHLYF 165
CN20_NS2       VEYSRYAYCRNCKSTIKPTWRYSQRRASESNVVRNRGRPESHLYF 165
RVC50_NS2      VEYSRYAYCRNCKSTIKPTWRYSQRRASESNVVRNRGRPESHLYF 165
               ******************************::*************
```

(C) VP1

```
CPV12_VP1       MAPPAKRARRGLVPPGYKYLGPGNSLDQGEPTNPSDAAAKEHDEAYAAYLRSGKNPYLYF 60
CN10_VP1        MAPPAKRARRGLVPPGYKYLGPGNSLDQGEPTNPSDAAAKEHDEAYAAYLRSGKNPYLYF 60
CN14_VP1        MAPPAKRARRGLVPPGYKYLGPGNSLDQGEPTNPSDAAAKEHDEAYAAYLRSGKNPYLYF 60
CN20_VP1        MAPPAKRARRGLVPPGYKYLGPGNSLDQGEPTNPSDAAAKEHDEAYAAYLRSGKNPYLYF 60
RVC50_VP1       MAPPAKRARRGLVPPGYKYLGPGNSLDQGEPTNPSDAAAKEHDEAYAAYLRSGKNPYLYF 60
                ************************************************************

CPV12_VP1       SPADQRFIDQTKDAKDWGGKIGHYFFRAKKAIAPVLTDTPDHPSTSRPTKPTKRSKPPPH 120
CN10_VP1        SPADQRFIDQTKDAKDWGGKIGHYFFRAKKAIAPVLTDTPDHPSTSRPTKPTKRSKPPPH 120
CN14_VP1        SPADQRFIDQTKDAKDWGGKIGHYFFRAKKAIAPVLTDTPDHPSTSRPTKPTKRSKPPPH 120
CN20_VP1        SPADQRFIDQTKDAKDWGGKIGHYFFRAKKAIAPVLTDTPDHPSTSRPTKPTKRSKPPPH 120
RVC50_VP1       SPADQRFIDQTKDAKDWGGKIGHYFFRAKKAIAPVLTDTPDHPSTSRPTKPTKRSKPPPH 120
                ************************************************************

CPV12_VP1       IFINLAKKKKAGAGQVKRDNLAPMSDGAVQPDGGQPAVRNERATGSGNGSGGGGGGGSGG 180
CN10_VP1        IFINLAKKKKAGAGQVKRDNLAPMSDGAVQPDGGQPAVRNERATGSGNGSGGGGGGGSGG 180
CN14_VP1        IFINLAKKKKAGAGQVKRDNLAPMSDGAVQPDGGQPAVRNERATGSGNGSGGGGGGGSGG 180
CN20_VP1        IFINLAKKKKAGAGQVKRDNLAPMSDGAVQPDGGQPAVRNERATGSGNGSGGGGGGGSGG 180
RVC50_VP1       IFINLAKKKKAGAGQVKRDNLAPMSDGAVQPDGGQPAVRNERATGSGNGSGGGGGGGSGG 180
                ************************************************************

CPV12_VP1       VGISTGTFNNQTEFKFLENGWVEITANSSRLVHLNMPESENYRRVVVNNMDKTAVNGNMA 240
CN10_VP1        VGISTGTFNNQTEFKFLENGWVEITANSSRLVHLNMPESENYRRVVVNNLDKTAVNGNMA 240
CN14_VP1        VGISTGTFNNQTEFKFLENGWVEITANSSRLVHLNMPESENYRRVVVNNLDKTAVNGNMA 240
CN20_VP1        VGISTGTFNNQTEFKFLENGWVEITANSSRLVHLNMPESENYRRVVVNNLDKTAVNGNMA 240
RVC50_VP1       VGISTGTFNNQTEFKFLENGWVEITANSSRLVHLNMPESENYRRVVVNNLDKTAVNGNMA 240
                *************************************************:**********

CPV12_VP1       LDDIHAQIVTPWSLVDANAWGVWFNPGDWQLIVNTMSELHLVSFEQEIFNVVLKTVSESA 300
CN10_VP1        LDDTHAQIVTPWSLVDANAWGVWFNPGDWQLIVNTMSELHLVSFEQEIFNVVLKTVSESA 300
CN14_VP1        LDDTHAQIVTPWSLVDANAWGVWFNPGDWQLIVNTMSELHLVSFEQEIFNVVLKTVSESA 300
CN20_VP1        LDDTHAQIVTPWSLVDANAWGVWFNPGDWQLIVNTMSELHLVSFEQEIFNVVLKTVSESA 300
RVC50_VP1       LDDTHAQIVTPWSLVDANAWGVWFNPGDWQLIVNTMSELHLVSFEQEIFNVVLKTVSESA 300
                *** ********************************************************

CPV12_VP1       TQPPTKVYNNDLTASLMVALDSNNTMPFTPAAMRSETLGFYPWKPTIPTPWRYYFQWDRT 360
CN10_VP1        TQPPTKVYNNDLTASLMVALDSNNTMPFTPAAMRSETLGFYPWKPTIPTPWRYYFQWDRT 360
CN14_VP1        TQPPTKVYNNDLTASLMVALDSNNTMPFTPAAMRSETLGFYPWKPTIPTPWRYYFQWDRT 360
CN20_VP1        TQPPTKVYNNDLTASLMVALDSNNTMPFTPAAMRSETLGFYPWKPTIPTPWRYYFQWDRT 360
RVC50_VP1       TQPPTKVYNNDLTASLMVALDSNNTMPFTPAAMRSETLGFYPWKPTIPTPWRYYFQWDRT 360
                ************************************************************

CPV12_VP1       LIPSHTGTSGTPTNIYHGTDPDDVQFYTIENSVPVHLLRTGDEFATGTFFFDCKPCRLTH 420
CN10_VP1        LIPSHTGTSGTPTNIYHGTDPDDVQFYTIENSVPVHLLRTGDEFATGTFFFDCKPCRLTH 420
CN14_VP1        LIPSHTGTSGTPTNIYHGTDPDDVQFYTIENSVPVHLLRTGDEFATGTFFFDCKPCRLTH 420
CN20_VP1        LIPSHTGTSGTPTNIYHGTDPDDVQFYTIENSVPVHLLRTGDEFTTGTFFFDCKPCRLTH 420
RVC50_VP1       LIPSHTGTSGTPTNIYHGTDPDDVQFYTIENSVPVHLLRTGDEFATGTFFFDCKPCRLTH 420
                ********************************************:***************

CPV12_VP1       TWQTNRALGLPPFLNSLPQSEGATNFGDIGVQQDKRRGVTQMGNTNYITEATIMRPAEVG 480
CN10_VP1        TWQTNRALGLPPFLNSLPQAEGGTNFGYIGVQQDKRRGVTQMGNTNYITEATIMRPAEVG 480
CN14_VP1        TWQTNRALGLPPFLNSLPQAEGGTNFGYIGVQQDKRRGVTQMGNTNYITEATIMRPAEVG 480
CN20_VP1        TWQTNRALGLPPFLNSLPQAEGGTNFGYIGVQQDKRRGVTQMGNTNYITEATIMRPAEVG 480
RVC50_VP1       TWQTNRALGLPPFLNSLPQAEGGTNFGYIGVQQDKRRGVTQMGNTNYITEATIMRPAEVG 480
                *******************:**.**** ********************************

CPV12_VP1       YSAPYYSFEASTQGPFKTPIAAGRGGAQTDENQAADGNPRYAFGRQHGQKTTTTGETPER 540
CN10_VP1        YSAPYYSFEASTQGPFKTPIAAGRGGAQTDENQAADGDPRYAFGRQHGQKTTTTGETPER 540
CN14_VP1        YSAPYYSFEASTQGPFKTPIAAGRGGAQTDENQAANGDPRYAFGRQHGQKTTTTGETPER 540
CN20_VP1        YSAPYYSFEASTQGPFKTPIAAGRGGAQTDENQAADGDPRYAFGRQHGQKTTTTGETPER 540
RVC50_VP1       YSAPYYSFEASTQGPFKTPIAAGRGGAQTDENQAADGDPRYAFGRQHGQKTTTTGETPER 540
                ***********************************:*:**********************

CPV12_VP1       FTYIAHQDTGRYPEGDWIQNINFNLPVTNDNVLLPTDPIGGKTGINYTNIFNTYGPLTAL 600
CN10_VP1        FTYIAHQDTGRYPEGDWIQNINFNLPVTNDNVLLPTDPIGGKTGINYTNIFNTYGPLTAL 600
CN14_VP1        FTYIAHQDTGRYPEGDWIQNINFNLPVTNDNVLLPTDPIGGKTGINYTNIFNTYGPLTAL 600
CN20_VP1        FTYIAHQDTGRYPEGDWIQNINFNLPVTNDNVLLPTDPIGGKTGINYTNIFNTYGPLTAL 600
RVC50_VP1       FTYIAHQDTGRYPEGDWIQNINFNLPVTNDNVLLPTDPIGGKTGINYTNIFNTYGPLTAL 600
                ************************************************************
```

```
CPV12_VP1       NNVPPVYPNGQIWDKEFDTDLKPRLHVNAPFVCQNNCPGQLFVKVAPNLTNEYDPDASAN 660
CN10_VP1        NNVPPVYPNGQIWDKEFDTDLKPRLHVNAPFVCQNNCPGQLFVKVAPNLTNEYDPDASAN 660
CN14_VP1        NNVPPVYPNGQIWDKEFDTDLKPRLHVNAPFVCQNNCPGQLFVKVAPNLTNEYDPDASAN 660
CN20_VP1        NNVPPVYPNGQIWDKEFDTDLKPRLHVNAPFVCQNNCPGQLFVKVAPNLTNEYDPDASAN 660
RVC50_VP1       NNVPPVYPNGQIWDKEFDTDLKPRLHVNAPFVCQNNCPGQLFVKVAPNLTNEYDPDASAN 660
                ************************************************************

CPV12_VP1       MSRIVTYSDFWWKGKLVFKAKLRASHTWNPIQQMSINVDNQFNYVPSNIGGMKIVYEKSQ 720
CN10_VP1        MSRIVTYSDFWWKGKLVFKAKLRASHTWNPIQQMSINVDNQFNYVPSNIGGMKIVYEKSQ 720
CN14_VP1        MSRIVTYSDFWWKGKLVFKAKLRASHTWNPIQQMSINVDNQFNYVPSNIGGMKIVYEKSQ 720
CN20_VP1        MSRIVTYSDFWWKGKLVFKAKLRASHTWNPIQQMSINVDNQFNYVPSNIGGMKIVYEKSQ 720
RVC50_VP1       MSRIVTYSDFWWKGKLVFKAKLRASHTWNPIQQMSINVDNQFNYVPSNIGGMKIVYEKSQ 720
                ************************************************************

CPV12_VP1       LAPRKLY 727
CN10_VP1        LAPRKLY 727
CN14_VP1        LAPRKLY 727
CN20_VP1        LAPRKLY 727
RVC50_VP1       LAPRKLY 727
                *******
```

(D) VP2

```
CPV12_VP2       MSDGAVQPDGGQPAVRNERATGSGNGSGGGGGGGGSGGVGISTGTFNNQTEFKFLENGWVE 60
RVC50_VP2       MSDGAVQPDGGQPAVRNERATGSGNGSGGGGGGGGSGGVGISTGTFNNQTEFKFLENGWVE 60
CN10_VP2        MSDGAVQPDGGQPAVRNERATGSGNGSGGGGGGGGSGGVGISTGTFNNQTEFKFLENGWVE 60
CN14_VP2        MSDGAVQPDGGQPAVRNERATGSGNGSGGGGGGGGSGGVGISTGTFNNQTEFKFLENGWVE 60
CN20_VP2        MSDGAVQPDGGQPAVRNERATGSGNGSGGGGGGGGSGGVGISTGTFNNQTEFKFLENGWVE 60
                ************************************************************

CPV12_VP2       ITANSSRLVHLNMPESENYRRVVVNNMDKTAVNGNMALDDIHAQIVTPWSLVDANAWGVW 120
RVC50_VP2       ITANSSRLVHLNMPESENYRRVVVNNLDKTAVNGNMALDDTHAQIVTPWSLVDANAWGVW 120
CN10_VP2        ITANSSRLVHLNMPESENYRRVVVNNLDKTAVNGNMALDDTHAQIVTPWSLVDANAWGVW 120
CN14_VP2        ITANSSRLVHLNMPESENYRRVVVNNLDKTAVNGNMALDDTHAQIVTPWSLVDANAWGVW 120
CN20_VP2        ITANSSRLVHLNMPESENYRRVVVNNLDKTAVNGNMALDDTHAQIVTPWSLVDANAWGVW 120
                **************************:************* *******************

CPV12_VP2       FNPGDWQLIVNTMSELHLVSFEQEIFNVVLKTVSESATQPPTKVYNNDLTASLMVALDSN 180
RVC50_VP2       FNPGDWQLIVNTMSELHLVSFEQEIFNVVLKTVSESATQPPTKVYNNDLTASLMVALDSN 180
CN10_VP2        FNPGDWQLIVNTMSELHLVSFEQEIFNVVLKTVSESATQPPTKVYNNDLTASLMVALDSN 180
CN14_VP2        FNPGDWQLIVNTMSELHLVSFEQEIFNVVLKTVSESATQPPTKVYNNDLTASLMVALDSN 180
CN20_VP2        FNPGDWQLIVNTMSELHLVSFEQEIFNVVLKTVSESATQPPTKVYNNDLTASLMVALDSN 180
                ************************************************************

CPV12_VP2       NTMPFTPAAMRSETLGFYPWKPTIPTPWRYYFQWDRTLIPSHTGTSGTPTNIYHGTDPDD 240
RVC50_VP2       NTMPFTPAAMRSETLGFYPWKPTIPTPWRYYFQWDRTLIPSHTGTSGTPTNIYHGTDPDD 240
CN10_VP2        NTMPFTPAAMRSETLGFYPWKPTIPTPWRYYFQWDRTLIPSHTGTSGTPTNIYHGTDPDD 240
CN14_VP2        NTMPFTPAAMRSETLGFYPWKPTIPTPWRYYFQWDRTLIPSHTGTSGTPTNIYHGTDPDD 240
CN20_VP2        NTMPFTPAAMRSETLGFYPWKPTIPTPWRYYFQWDRTLIPSHTGTSGTPTNIYHGTDPDD 240
                ************************************************************

CPV12_VP2       VQFYTIENSVPVHLLRTGDEFATGTFFFDCKPCRLTHTWQTNRALGLPPFLNSLPQSEGA 300
RVC50_VP2       VQFYTIENSVPVHLLRTGDEFATGTFFFDCKPCRLTHTWQTNRALGLPPFLNSLPQAEGG 300
CN10_VP2        VQFYTIENSVPVHLLRTGDEFATGTFFFDCKPCRLTHTWQTNRALGLPPFLNSLPQAEGG 300
CN14_VP2        VQFYTIENSVPVHLLRTGDEFATGTFFFDCKPCRLTHTWQTNRALGLPPFLNSLPQAEGG 300
CN20_VP2        VQFYTIENSVPVHLLRTGDEFTTGTFFFDCKPCRLTHTWQTNRALGLPPFLNSLPQAEGG 300
                *********************:**********************************:**.

CPV12_VP2       TNFGDIGVQQDKRRGVTQMGNTNYITEATIMRPAEVGYSAPYYSFEASTQGPFKTPIAAG 360
RVC50_VP2       TNFGYIGVQQDKRRGVTQMGNTNYITEATIMRPAEVGYSAPYYSFEASTQGPFKTPIAAG 360
CN10_VP2        TNFGYIGVQQDKRRGVTQMGNTNYITEATIMRPAEVGYSAPYYSFEASTQGPFKTPIAAG 360
CN14_VP2        TNFGYIGVQQDKRRGVTQMGNTNYITEATIMRPAEVGYSAPYYSFEASTQGPFKTPIAAG 360
CN20_VP2        TNFGYIGVQQDKRRGVTQMGNTNYITEATIMRPAEVGYSAPYYSFEASTQGPFKTPIAAG 360
                **** *******************************************************

CPV12_VP2       RGGAQTDENQAADGNPRYAFGRQHGQKTTTTGETPERFTYIAHQDTGRYPEGDWIQNINF 420
RVC50_VP2       RGGAQTDENQAADGDPRYAFGRQHGQKTTTTGETPERFTYIAHQDTGRYPEGDWIQNINF 420
CN10_VP2        RGGAQTDENQAADGDPRYAFGRQHGQKTTTTGETPERFTYIAHQDTGRYPEGDWIQNINF 420
CN14_VP2        RGGAQTDENQAANGDPRYAFGRQHGQKTTTTGETPERFTYIAHQDTGRYPEGDWIQNINF 420
CN20_VP2        RGGAQTDENQAADGDPRYAFGRQHGQKTTTTGETPERFTYIAHQDTGRYPEGDWIQNINF 420
                ************:*:*********************************************

CPV12_VP2       NLPVTNDNVLLPTDPIGGKTGINYTNIFNTYGPLTALNNVPPVYPNGQIWDKEFDTDLKP 480
RVC50_VP2       NLPVTNDNVLLPTDPIGGKTGINYTNIFNTYGPLTALNNVPPVYPNGQIWDKEFDTDLKP 480
CN10_VP2        NLPVTNDNVLLPTDPIGGKTGINYTNIFNTYGPLTALNNVPPVYPNGQIWDKEFDTDLKP 480
CN14_VP2        NLPVTNDNVLLPTDPIGGKTGINYTNIFNTYGPLTALNNVPPVYPNGQIWDKEFDTDLKP 480
CN20_VP2        NLPVTNDNVLLPTDPIGGKTGINYTNIFNTYGPLTALNNVPPVYPNGQIWDKEFDTDLKP 480
                ************************************************************

CPV12_VP2       RLHVNAPFVCQNNCPGQLFVKVAPNLTNEYDPDASANMSRIVTYSDFWWKGKLVFKAKLR 540
RVC50_VP2       RLHVNAPFVCQNNCPGQLFVKVAPNLTNEYDPDASANMSRIVTYSDFWWKGKLVFKAKLR 540
CN10_VP2        RLHVNAPFVCQNNCPGQLFVKVAPNLTNEYDPDASANMSRIVTYSDFWWKGKLVFKAKLR 540
CN14_VP2        RLHVNAPFVCQNNCPGQLFVKVAPNLTNEYDPDASANMSRIVTYSDFWWKGKLVFKAKLR 540
CN20_VP2        RLHVNAPFVCQNNCPGQLFVKVAPNLTNEYDPDASANMSRIVTYSDFWWKGKLVFKAKLR 540
                ************************************************************

CPV12_VP2       ASHTWNPIQQMSINVDNQFNYVPSNIGGMKIVYEKSQLAPRKLY 584
RVC50_VP2       ASHTWNPIQQMSINVDNQFNYVPSNIGGMKIVYEKSQLAPRKLY 584
CN10_VP2        ASHTWNPIQQMSINVDNQFNYVPSNIGGMKIVYEKSQLAPRKLY 584
CN14_VP2        ASHTWNPIQQMSINVDNQFNYVPSNIGGMKIVYEKSQLAPRKLY 584
CN20_VP2        ASHTWNPIQQMSINVDNQFNYVPSNIGGMKIVYEKSQLAPRKLY 584
                ********************************************
```
