# Supplementary material for: Molecular Investigation of Canine Parvovirus-2 (CPV-2) Outbreak in Nevis Island: Analysis of the Nearly Complete Genomes of CPV-2 Strains from the Caribbean Region
Source: Viruses. 2021 Jun 6;13(6):1083. doi: 10.3390/v13061083 (PMC8227521; doi:10.3390/v13061083)

Supplementary material S8. Phylogenetic analysis of the nearly complete genomes of canine parvovirus-2 (CPV-2) strains from St. Kitts and Nevis with those of other CPV-2 strains. The tree was created using the General Time Reversible model with gamma-distributed rate variation among sites and 1000 bootstrap replicates. The name of the strain/CPV-2 antigenic variant/place/year of detection are shown for the CPV-2 strains from St. Kitts and Nevis, whilst the GenBank accession number/CPV-2 antigenic variant/place/year of detection have been mentioned for the other CPV-2 strains. The two major CPV-2 clades (CPV-2 and CPV-2a) is demarcated with a brown and a light gray bar, respectively. The CPV-2 clade consists of the earliest CPV-2 strains from the late 1970s (the CPV-2 variants), whilst the 'CPV-2a clade' is composed of the CPV-2a, CPV-2b and CPV-2c antigenic variants [16]. Feline panleukopenia virus (FPV) strains were included in the analysis. Sky blue, purple, yellow, pink, orange, green and red circles indicates that the CPV-2 strain was detected in Asia, Africa, Australia, Europe, North America, South America, and St. Kitts and Nevis, respectively. Scale bar, 0.01 substitutions per nucleotide. Bootstrap values of <70 are not shown.

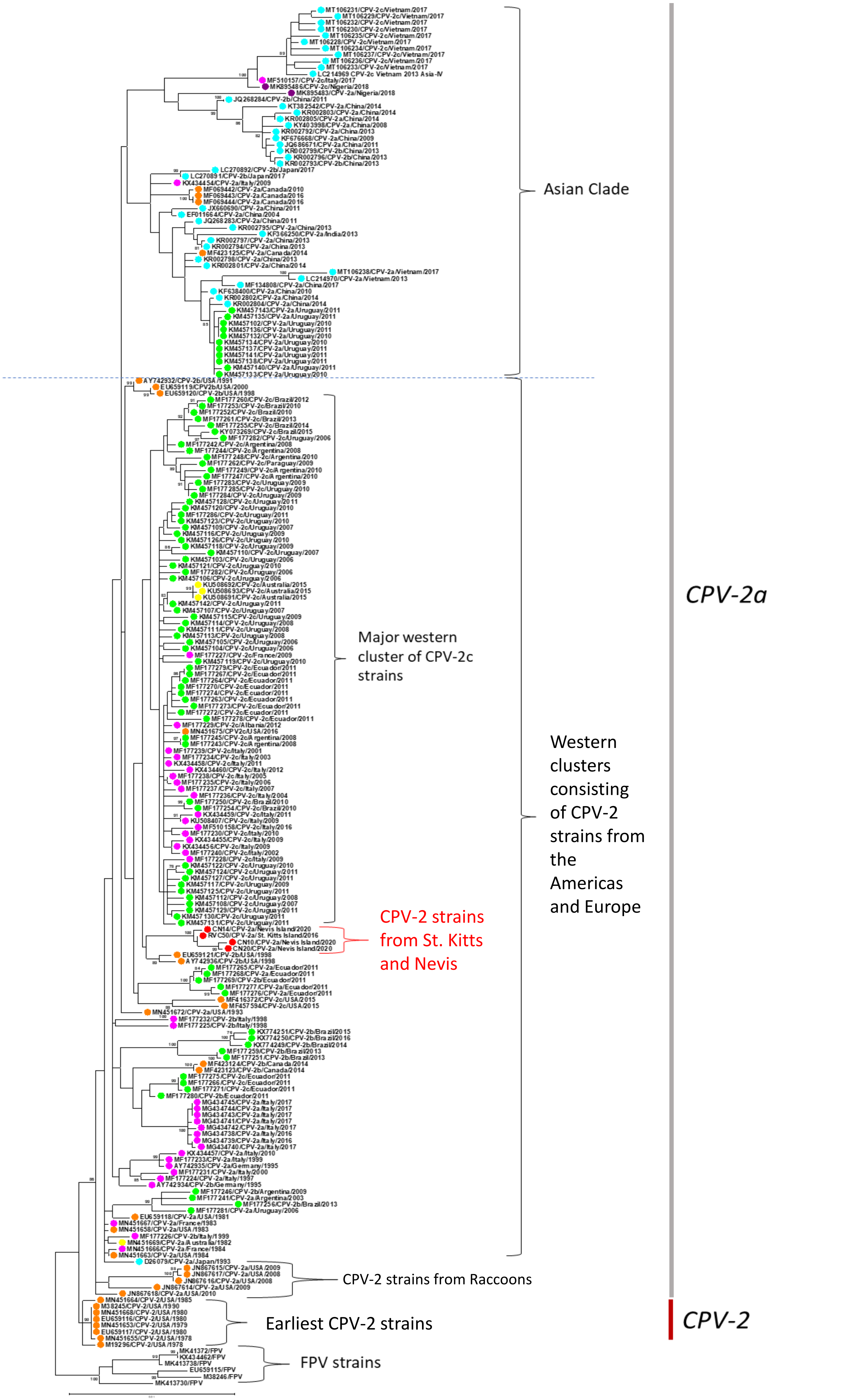

Supplement: Supplementary file 1 [file viruses-13-01083-s001.zip › Supplementary materials_R1/Supplementary material S8.pdf]
