# Supplementary material for: Molecular Investigation of Canine Parvovirus-2 (CPV-2) Outbreak in Nevis Island: Analysis of the Nearly Complete Genomes of CPV-2 Strains from the Caribbean Region
Source: Viruses. 2021 Jun 6;13(6):1083. doi: 10.3390/v13061083 (PMC8227521; doi:10.3390/v13061083)

Supplementary material S9. Phylogenetic analysis of the complete VP2 coding sequences of canine parvovirus-2 (CPV-2) strains from St. Kitts and Nevis with those of other CPV-2 strains. The Maximum likelihood tree was created using the General Time Reversible model with gamma-distributed rate variation among sites and 1000 bootstrap replicates. The name of the strain/CPV-2 antigenic variant/place/year of detection are shown for the CPV-2 strains from St. Kitts and Nevis, whilst the GenBank accession number/CPV-2 antigenic variant/place/year of detection have been mentioned for the other CPV-2 strains. Feline panleukopenia virus (FPV) strains were included in the analysis. Scale bar, 0.01 substitutions per nucleotide. Bootstrap values of <70 are not shown.

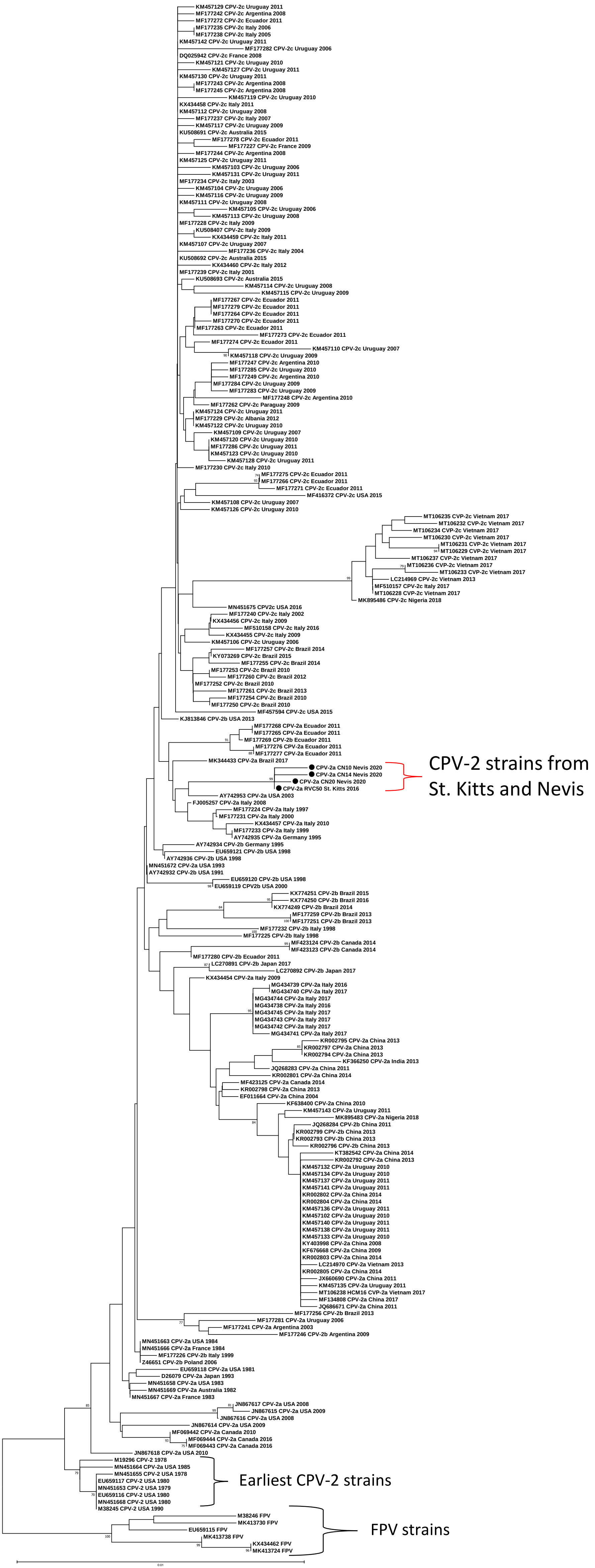

Supplement: Supplementary file 1 [file viruses-13-01083-s001.zip › Supplementary materials_R1/Supplementary material S9.pdf]
